# Three classes of hemoglobins are required for optimal vegetative and reproductive growth of *Lotus japonicus*: genetic and biochemical characterization of LjGlb2-1

**DOI:** 10.1093/jxb/erab376

**Published:** 2021-08-13

**Authors:** Irene Villar, Maria C Rubio, Laura Calvo-Begueria, Carmen Pérez-Rontomé, Estibaliz Larrainzar, Michael T Wilson, Niels Sandal, Luis A Mur, Longlong Wang, Brandon Reeder, Deqiang Duanmu, Toshiki Uchiumi, Jens Stougaard, Manuel Becana

**Affiliations:** 1 Departamento de Nutrición Vegetal, Estación Experimental de Aula Dei, Consejo Superior de Investigaciones Científicas, Apartado 13034, 50080 Zaragoza, Spain; 2 Department of Sciences, Institute for Multidisciplinary Research in Applied Biology, Campus Arrosadía, Universidad Pública de Navarra, 31006 Pamplona, Spain; 3 School of Life Sciences, Essex University, Wivenhoe Park, Colchester CO4 3SQ, UK; 4 Department of Molecular Biology and Genetics, Aarhus University, Gustav Wieds Vej 10, 8000 Aarhus C, Denmark; 5 Aberystwyth University, Institute of Biological, Environmental and Rural Sciences, Aberystwyth, SY23 3DA, Wales, UK; 6 State Key Laboratory of Agricultural Microbiology, College of Life Science and Technology, Huazhong Agricultural University, Wuhan, 430070, China; 7 Graduate School of Science and Engineering, Kagoshima University, 1-21-35 Korimoto, Kagoshima 890-0065, Japan; 8 University of Warwick, UK

**Keywords:** Hemoglobins, *Lotus japonicus*, *Medicago truncatula*, mutants, nitric oxide, symbiosis

## Abstract

Legumes express two major types of hemoglobins, namely symbiotic (leghemoglobins) and non-symbiotic (phytoglobins), with the latter being categorized into three classes according to phylogeny and biochemistry. Using knockout mutants, we show that all three phytoglobin classes are required for optimal vegetative and reproductive development of *Lotus japonicus*. The mutants of two class 1 phytoglobins showed different phenotypes: *Ljglb1-1* plants were smaller and had relatively more pods, whereas *Ljglb1-2* plants had no distinctive vegetative phenotype and produced relatively fewer pods. Non-nodulated plants lacking LjGlb2-1 showed delayed growth and alterations in the leaf metabolome linked to amino acid processing, fermentative and respiratory pathways, and hormonal balance. The leaves of mutant plants accumulated salicylic acid and contained relatively less methyl jasmonic acid, suggesting crosstalk between LjGlb2-1 and the signaling pathways of both hormones. Based on the expression of *LjGlb2-1* in leaves, the alterations of flowering and fruiting of nodulated *Ljglb2-1* plants, the developmental and biochemical phenotypes of the mutant fed on ammonium nitrate, and the heme coordination and reactivity of the protein toward nitric oxide, we conclude that LjGlb2-1 is not a leghemoglobin but an unusual class 2 phytoglobin. For comparison, we have also characterized a close relative of LjGlb2-1 in *Medicago truncatula*, MtLb3, and conclude that this is an atypical leghemoglobin.

## Teaser

Knockout mutants of *Lotus japonicus* demonstrate that three classes of phytoglobins are required for optimal vegetative and reproductive development. The most unusual hemoglobin LjGlb2-1 is genetically and biochemically characterized.

## Introduction

Legumes contain two major types of hemoglobins, namely symbiotic or leghemoglobins (Lbs) and non-symbiotic or phytoglobins (Glbs). In general, Lbs are expressed in nodules at millimolar concentrations and are present as a mixture of components, the relative proportions of which vary with nodule age and stress conditions; however, the reason for the existence of these multiple components is uncertain (see review by Larrainzar *et al.*, 2020). The major function of Lbs is to buffer O_2_ in the infected cells so that bacteroids can simultaneously sustain high respiration and N_2_ fixation rates (Appleby, 1984; Ott *et al.*, 2005; Wang *et al.*, 2019). In contrast, Glbs are present at low micromolar concentrations in most plant tissues (Hill, 2012). They are categorized into three phylogenetic classes, at least two of which are present in legumes. Class 1 Glbs have a very high O_2_ affinity and hence are unlikely to transport and deliver O_2_ for metabolic reactions. Among other functions, they are involved in plant responses and adaptations to hypoxia and in nitric oxide (NO) homeostasis (Igamberdiev and Hill, 2004). These Glbs are also crucial for the onset and functioning of the legume–*Rhizobium* symbiosis by modulating NO levels (Shimoda *et al.*, 2005; Fukudome *et al.*, 2016; Berger *et al.*, 2020). Class 2 Glbs have homology with Lbs and show moderate O_2_ affinity and NO-scavenging activity, which is compatible with a role in modulating O_2_ and/or NO levels *in vivo* (Hebelstrup and Jensen, 2008; Smagghe *et al.*, 2009). Class 3 Glbs share homology with the ‘truncated’ hemoglobins of prokaryotes and show low O_2_ affinity. They have unknown functions, although roles in the suppression of plant defense responses during rhizobial and mycorrhizal symbioses and in protection from nitrosative stress have been suggested (Vieweg *et al.*, 2005).

Among other factors, the functions of Lbs and Glbs are associated with protein structure and the heme reactivity with physiological ligands such as O_2_ and NO. Class 1 and class 2 Glbs are hexacoordinate because they have the fifth (proximal) and sixth (distal) positions of the heme iron coordinated to His or, more rarely, to other amino acid residues. In contrast, Lbs and class 3 Glbs are pentacoordinate because they have a proximal His residue but lack an amino acid residue at the distal position (Watts *et al.*, 2001; Smagghe *et al.*, 2009). The degree of hexa- or pentacoordination of the hemes has a large effect on their reactivities, as illustrated by comparing the O_2_ affinities of Lbs and class 1 Glbs. The hemes of Glbs and Lbs are involved in several reactions with NO (see review by Becana *et al.*, 2020). One of them is the oxidation of NO to NO_3_^–^ by the NO dioxygenase (NOD) activity of oxyferrous hemoglobin (2+O_2_) (Igamberdiev and Hill, 2004), and another is the reduction of nitrite to NO by the nitrite reductase (NiR) activity of deoxyferrous hemoglobin (2+) (Sturms *et al.*, 2011; Tiso *et al.*, 2012). Both yield ferric hemoglobin (3+) that needs to be reduced back to the 2+ form in order to sustain the reactions.

The genomes of the model legumes *Medicago truncatula* and *Lotus japonicus* contain multiple *Lb* and *Glb* genes. A search of the latest genome version of *M. truncatula* ecotype A17 (Mtv5.0) reveals that it may express up to twelve Lbs (MtLb1 to MtLb12), three class 1 Glbs (MtGlb1-1, MtGlb1-2, MtGlb1-3), and two class 3 Glbs (MtGlb3-1, MtGlb3-2) (Berger *et al.*, 2020; Larrainzar *et al.*, 2020). Similarly, a search of the latest genome version of *L. japonicus* ecotype Gifu (Ljv1.2; Kamal *et al.*, 2020) shows that it encodes two class 1 Glbs (LjGlb1-1, LjGlb1-2), one putative class 2 Glb (LjGlb2-1), one uncharacterized hemoglobin (provisionally designated as LjGlb2-2), and two class 3 Glbs (LjGlb3-1, LjGlb3-2) (Larrainzar *et al.*, 2020). The nodulation phenotypes of mutants deficient in LjGlb1-1 (Fukudome *et al.*, 2016) or its homolog MtGlb1-1 (Berger *et al.*, 2020) have been examined and both proteins have been found to be important for the onset of symbiosis. However, the phenotype of these mutants at the reproductive stage, as well as the phenotypes of other hemoglobin mutants, are unknown.

In the first part of this study, we provide detailed information on the symbiotic performance, from seedling to seed- producing mature plants, of *L. japonicus* knockout mutants for *LjGlb1-1*, *LjGlb1-2*, *LjGlb2-1*, and *LjGlb3-2*. The mutants, which bear insertions of the retrotransposon *LORE1*, were generated in Denmark (Urbański *et al.*, 2012; Małolepszy *et al.*, 2016) or Japan (Fukai *et al.*, 2010). Unfortunately, there are no insertional mutants available in either of the two *LORE1* collections for *LjGlb2-2* and *LjGlb3-1*. The second part of our study focuses on characterizing LjGlb2-1 because of its peculiarities. The protein shares high sequence identity with Lbs but, unlike them, it is hexacoordinate in the 3+ form (Calvo-Begueria *et al.*, 2017) and is expressed also in the roots (Bustos-Sanmamed *et al.*, 2011). Finally, because MtLb3 is phylogenetically related to LjGlb2-1 (Larrainzar *et al.*, 2020), the tissue location and biochemical properties of both proteins are compared to help determine if they are functional homologs.

## Materials and methods

### Biological materials and growth conditions

Plants of *Lotus japonicus* ecotype Gifu B-129 mutated in *Glb* genes were obtained from the *LORE1* mutant collection (Lotus Base, https://lotus.au.dk; Mun *et al.*, 2016) except for P0494, which was obtained from Legume Base (National BioResource Project, https://www.legumebase.brc.miyazaki-u.ac.jp/). *LORE1* is an endogenous 5-kb retrotransposon that inserts in the genome to create loss-of-function mutations. *LORE1* mutants are stable, the plants are not transgenic, mutations are heritable, and mutant phenotypes are maintained through generations (Madsen *et al.*, 2005; Urbański *et al.*, 2012; Małolepszy *et al.*, 2016). Plants of *L. japonicus* ecotype MG-20 mutated in the three *Lb* genes were generated using CRISPR/Cas9 (Wang *et al.*, 2016).

Seeds of *L. japonicus* were gently scarified with sand paper, disinfected for 20 min with 2% sodium hypochlorite, washed, and imbibed overnight in the dark at room temperature. They were then transferred to 0.5 % (w/v) agar plates and kept in the dark at 4 °C for 2 d. For germination, plates were placed vertically for 3 d in the dark at 23 °C and for 2 d more with a 16/8-h light/dark photoperiod (140 μmol photons m^–2^ s^–1^) at 23/21 °C. The seedlings were then transferred to plates with Jensen medium (Pajuelo and Stougaard, 2005) and grown with a 16-h photoperiod as indicated above.

Seeds of *Medicago truncatula* ecotype Jemalong A17 were scarified with sulfuric acid for 8 min, sterilized with 4% sodium hypochlorite for 2 min, washed, and imbibed for 8 h at 25 °C. Seeds were then stratified for 2 d at 4 °C on 0.5% (w/v) agar plates and germinated at 23 °C for 1 d in the dark. Nodulation and growth conditions are described below, together with the protocol for producing hairy roots.

### Plant phenotyping

The phenotypes of the *L. japonicus* Gifu mutant lines were examined under non-nodulating and nodulating conditions. To phenotype non-nodulated plants, 5-d-old seedlings were grown for 3 weeks on Jensen plates supplemented with 1.5 mM NH_4_NO_3_ and growth parameters were measured (shoot and root lengths and weights, leaf numbers and weights). To phenotype nodulated plants, 5-d-old seedlings were transferred to Jensen plates, inoculated with *Mesorhizobium loti* strain R7A (10^7^ cells per root), and grown for 4 or 5 weeks. Shoot and root lengths and weights, and leaf and nodule numbers and weights were then measured. After this first phenotyping, nodulated plants were transferred to pots (12.5 cm diameter) containing vermiculite and were watered twice a week with B&D nutrient solution (Broughton and Dilworth, 1971) supplemented with 0.25 mM NH_4_NO_3_. The growth phenotypes were examined again at 8 weeks post-germination. Finally, a set of nodulated plants was left to reach the reproductive stage in order to examine flowers and fruits. Plant material was harvested and immediately placed in liquid nitrogen, and then stored at –80 °C until use.

### Complementation of the *lb123* mutant

The genomic sequences of *LjLb2* and *LjGlb2-1* were amplified using gene-specific primers (*LjLb2*–*Bam*HI–F, 5´-AAAGGATCCATGGGTTT CACTGCACAG-3´; *LjLb2*–*Asc*I–R, 5´-GATGGCGCGCCTCAG CTCATTGCCTTCTTAA-3´; *LjGlb2-1*–*Bam*HI–F, 5´-CATGGATCCATGGCTACATTCAGTGAGGAG-3´; *LjGlb2-1*–*Asc*I–R, 5´-ACTGGCGCGCCTCAACTCATTCCCTTTTTAATCAC-3´), cloned into pC1300-sGFP coupled to the *LjLb2* promoter, and the plasmids were introduced into *Agrobacterium rhizogenes* strain LBA1334. Hairy root transformation was then performed on the *lb123* triple-mutant as described by Wang *et al.* (2019). Plants with positive transgenic roots were identified by GFP fluorescence, transferred to pots containing perlite/vermiculite (1:1), and inoculated with *Mesorhizobium loti* strain MAFF303099. Symbiotic phenotypes were examined at 5 weeks post-inoculation, which included measurement of nitrogenase activity using the acetylene reduction assay, as described by Wang *et al.* (2019).

### Cloning of the *MtLb3* promoter, expression in hairy roots, and histochemical localization

A DNA fragment encoding ~2 kb upstream of the predicted coding region of *MtLb3* was amplified by PCR using the primers 5´-CACCTCTGCCTACAACTCTATTTGTCAG-3´ and 5´-GTGTTTGTGTTTTTTGCTTTTCTT-3´. The promoter region was cloned into the pENTR/D-TOPO vector (Invitrogen), sequenced, and subcloned into the binary vector pBGWFS7 (Karimi *et al.*, 2002) using Gateway technology to make a transcriptional fusion with the *uidA* gene. The empty vector pBGWFS7 was used as a negative control for transformations. Plasmids were electroporated into *Agrobacterium rhizogenes* ARqua1, and transformed hairy roots were generated in *M. truncatula* Jemalong A17 as described by Boisson-Dernier *et al.* (2001). At 4 weeks old, transformed plantlets were transferred to pots containing a perlite/vermiculite mixture (1:1) and inoculated with *Sinorhizobium meliloti* 1021. The *MtLb3* promoter activity in the *M. truncatula* roots and nodules was localized histochemically as previously described (Villar *et al.*, 2020). Briefly, roots and nodules were pre-fixed in 0.3% (w/v) paraformaldehyde in 100 mM sodium phosphate buffer (pH 7.0) for 30 min, and then washed in buffer. Samples were then vacuum-infiltrated for 20 min in β-glucuronidase (GUS) staining solution containing 1 mg ml^−1^ X-Gluc (5-bromo-4-chloro-3-indolyl-β-D-glucuronic acid), 2.5 mM potassium ferricyanide, 2.5 mM potassium ferrocyanide, 10 mM EDTA, 0.1% (v/v) Triton X-100, and 100 mM sodium phosphate (pH 7.0), and incubated for 2–4 h at 37 °C in the dark. Finally, samples were washed in 70% ethanol and phosphate buffer, mounted on glass slides in 50% glycerol (prepared in buffer), and visualized using a Leica M165 FC stereomicroscope with transmitted light. Some nodules were embedded in 5% (w/v) agarose and 60-µm sections made using a vibratome (Leica VT1000S).

### Metabolomics

Leaf material (100 mg) was ground and extracted in 1 ml chloroform/methanol/water (1:3:1) solution. The samples were then centrifuged at 6000 *g* for 1 min at 4 °C. The supernatant was collected and dried in a vacuum-concentrator without heating. After drying, 200 μl of 50% methanol was added and 70 μl was transferred into a HPLC glass vial with a 0.2 ml flat-bottom micro-insert. Samples were run randomly using an autosampler with a tray temperature of 15 °C. The sample injected was set at a volume of 20 μl into a flow volume of 60 μl ml^–1^ water/methanol (70/30%) using a Surveyor liquid chromatography system (Thermo Scientific). Flow-infusion electrospray–high-resolution mass spectroscopy was performed using an Exactive Plus Orbitrap MS (Thermo Scientific). Mass ions (*m*/*z*) were generated in both positive and negative ionization modes over four scan ranges (15–110, 100–220, 210–510, and 500–1200) with an acquisition time of 5 min. Individual ionization peak values were normalized as a percentage of the total ion count for each sample. Values of *m*/*z* that varied significantly between the experimental classes were identified by one-way ANOVA using Benjamini–Hochberg adjustment in order to control the false-discovery rates. The high-resolution accurate mass values of statistically significant *m*/*z* were used to interrogate the Kyoto Encyclopedia of Genes and Genomes (KEGG) database (http://www.genome.jp/kegg/) and the Direct Infusion Metabolite database (https://dimedb.ibers.aber.ac.uk/). Identification was based on the ‘MS Peaks to Pathways’ algorithm (Barnes *et al.*, 2016) (tolerance = 5 ppm; reference library, *Arabidopsis thaliana*). This involved metabolites being annotated using the KEGG database considering the following possible adducts: [M]^+^, [M+H]^+^, [M+NH_4_]^+^, [M+Na]^+^, [M+K]^+^, [M-NH_2_+H]^+^, [M-CO_2_H+H]^+^, [M-H_2_O+H]^+^, [M]^−^, [M−H]^−^, [M+Na−2H]^−^, [M+Cl]^−^, [M+K−2H]^−^. Correlations between multiple adducts of a metabolite were used in the identification process.

### Expression analyses

Total RNA was extracted from nodules (20–40 mg), roots (80–100 mg), and leaves (50–60 mg) using the RNAqueous isolation kit (ThermoFisher Scientific), treated with DNaseI (Roche), and cDNA was synthesized using MMLV-RT (Promega). Quantitative reverse-transcription (qRT-)PCR analyses were performed using a 7500 Real-Time PCR System (Applied Biosystems) as previously described (Rubio *et al.*, 2019). Primer sequences and efficiencies are given in Supplementary Table S1. Normalized relative quantities (NRQs) were calculated using *LjUbiquitin*, *LjATP synthase*, and *LjeIF4A* as the reference genes. At least three biological replicates per treatment were used and reactions were carried out in triplicate. Statistical analyses were performed using log_2_(NRQ)-transformed data and one-tailed Student’s *t*-tests for comparisons of means.

### Protein expression and purification

The ORFs encoding LjGlb2-1, MtLb3, and MtLb3-C135S were cloned into the Champion pET11a expression vector (Novagene) and expressed with an N-terminal Strep-tag in *Escherichia coli* C41(DE3) cells (Lucigen, Middleton, MI, USA). Cells were pre-cultured overnight in 100 ml of LB medium with 100 µg ml^–1^ ampicillin at 37 °C with mild agitation (200 rpm). Then, 10 ml of the pre-culture was used to inoculate 1 l of Terrific Broth medium with 100 µg ml^–1^ ampicillin, and cells were incubated at the same conditions as above until an OD at 600 nm of 0.6–0.7 was reached. Isopropyl β-D-1-thiogalactopyranoside was then added to the medium up to a concentration of 0.5 mM, and the cells were grown at 28 °C for 16 h. The cells were then washed in phosphate buffer and stored at –80 ºC for no longer than 3 weeks. For the purification protocol, cells were resuspended in 20 mM Tris (pH 8.0) containing 150 mM NaCl, then sonicated (3 × 2 min), and cleared by centrifugation. The supernatant was loaded onto a StrepTactin Sepharose High Performance column (GE Healthcare), previously equilibrated with the same buffer. After loading the protein, the column was washed with at least five volumes of buffer, and the recombinant proteins were eluted with buffer containing 2.5 mM desthiobiotin (Merck). The proteins were dialysed, concentrated, oxidized with ferricyanide, desalted through a NAP-5 mini-column (GE Healthcare) equilibrated with the same buffer without NaCl, and concentrated again. Purified proteins were quantified in the ferric form using an extinction coefficient of 150 mM^–1^ cm^–1^ for the Soret band.

### UV-visible spectra

The UV-visible spectra of the 3+ and 2+ forms, as well as the spectra of their complexes, were obtained as previously described (Villar *et al.*, 2020). Spectra were recorded immediately after production of the hemoglobin complexes using a 0.1-cm cuvette with a Lambda 25 spectrophotometer (Perkin-Elmer). Hemoglobin concentrations are indicated in the legends of Supplementary Figs S1, S2, and S3 for LjGlb2-1, MtLb3, and MtLb3-C135S, respectively.

### NO binding, NO dioxygenase activity, and NiR activity

For measurement of NO binding, 2+ hemoglobins (5 μM) in 50 mM potassium phosphate buffer (pH 7.0) and 150 mM NaCl were degassed in a glass tonometer connected to a supply of argon gas and a vacuum pump. Repeated degassing cycles were performed to remove O_2_. The NO donor, proli-NONOate (Cayman Chemical), was prepared in 25 mM NaOH to a concentration of 45 mM and then purged with argon gas. The concentration of proli-NONOate was checked by UV spectroscopy using an extinction coefficient at 252 nm of 8400 M^−1^ cm^−1^ (Maragos *et al.*, 1991). Solutions containing NO were prepared by injecting into the degassed buffer using a Hamilton syringe. The degassed proteins were transferred anaerobically to a 10-ml glass Hamilton syringe and mixed with the NO donor at a 1:1 ratio using an Applied Photophysics SX20 stopped-flow instrument fitted with a diode array spectrophotometer. The final concentrations of 2+ hemoglobin and NO were 2.5 μM and 10–160 μM, respectively. Kinetics were performed at 20 °C.

To measure NOD activity, 2+ hemoglobins (5 μM) in the same buffer as above were oxygenated by passing them through Sephadex G-25 (NAP-5) mini-columns. Complete oxygenation was checked using an Agilent 8453 diode array spectrophotomer. The buffer and NO donor were prepared as for the NO-binding experiments. Finally, 2+O_2_ hemoglobins were rapidly mixed with the NO donor (1:1) at final concentrations of 2.5 μM and 5–160 μM, respectively, using the SX20 stopped-flow instrument mentioned above pre-cooled to 10 °C.

To assay NiR activity, degassed 2+ hemoglobin was treated with NaNO_2_ dissolved in buffer with sodium dithionite. The final concentrations of 2+ hemoglobin and NaNO_2_ were 2.5 μM and 0.05–1.0 mM, respectively. Reactions were performed at room temperature. Optical spectra were recorded using an Agilent 8453 diode array spectrophotometer. Rate constants were determined from time-courses (0–200 s) by following the conversion of 2+ to 2+NO hemoglobin in the Soret band and then by fitting the data to exponential functions using the least-square method in the Microsoft Excel solver program.

## Results

### *Lotus japonicus* plants deficient in Glbs display gene-specific alterations in the vegetative and reproductive stages

The first objective of this study was to examine the phenotypes at the vegetative and reproductive stages of *L. japonicus* plants lacking Glbs under nodulating conditions. To do this, we obtained homozygous seeds of mutant lines bearing exonic insertions of the *LORE1* retrotransposon. Supplementary Table S2 lists the gene names, their identification numbers in the Gifu genome (Kamal *et al.*, 2020), the line names, and the tag insertion positions. It also shows the residual *LjGlb* transcript levels of the mutant plants, which were 1–7% of those of the wild-type (WT) plants, confirming that all the lines used in our study are virtually null-mutants. The *Ljglb1-1* mutant has previously been described by Fukudome *et al.* (2016). For the *Ljglb1-2* and *Ljglb2-1* mutants, only one line was selected for each because they bear single exonic insertions; in contrast, two independent lines were required to ascertain the phenotype of the *Ljglb3-2* plants because they contain multiple exonic insertions (LotusBase; Małolepszy *et al.*, 2016).

**Fig. 1.**
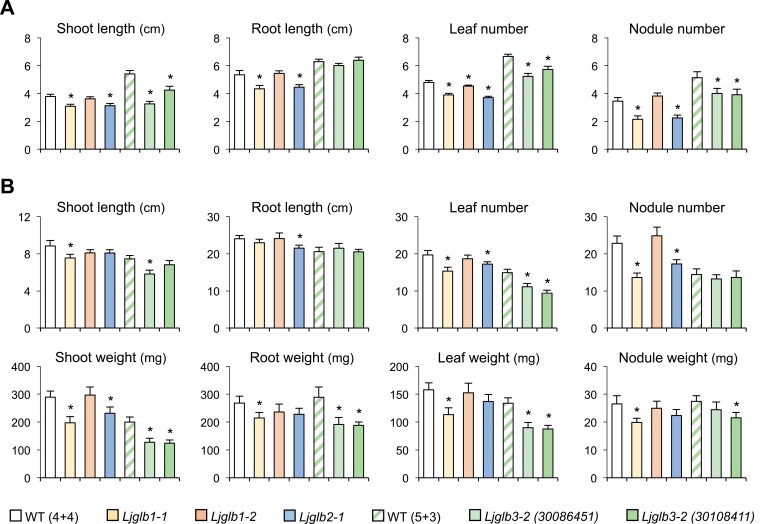
Growth phenotypes of nodulated *Lotus japonicus* mutants deficient in LjGlb1-1, LjGlb1-2, LjGlb2-1, or LjGlb3-2. Plants were phenotyped at (A) 4 weeks old except for *Ljglb3-2*, which was assessed at 5 weeks old, and (B) at 8 weeks old (all lines). Two independent lines were used to phenotype the *Ljglb3-2* plants, *30086451* and *30108411*. WT (4+4) indicates the wild-type used for *Ljglb1-1*, *Ljglb1-2*, and *Ljglb2-1* (4 weeks in plates, 4 weeks in pots); WT (5+3) indicates the wild-type used for the *Ljglb3-2* lines (5 weeks in plates, 3 weeks in pots). Data are means (±SE) of 10–25 plants representative of two or three sets of plants grown independently. Significant differences compared with the relevant WT were determined using Student’s *t*-test: *P<0.05.

The mutant lines for all four genes were phenotyped under nodulating conditions. Young plants (4 weeks old) of the *Ljglb1-1* and *Ljglb2-1* mutants had smaller shoots and roots as well as fewer leaves and nodules than the WT plants (Fig. 1A). The older plants (8 weeks old) of both mutants had lower leaf and nodule numbers and shoot weights, and the *Ljglb1-1* plants also had smaller shoots and reduced weights of roots, leaves, and nodules (Fig. 1B). In contrast, the *Ljglb1-2* mutant showed a similar phenotype to the WT plants both at 4 and 8 weeks old. Nodulated young plants (5 weeks old) of the two *Ljglb3-2* mutant lines had smaller shoots and fewer leaves than the WT (Fig. 1A), whereas the older plants (8 weeks old) had fewer leaves and reduced weights of shoots, roots, and leaves. Plants of line *30086451* also had shorter shoots (Fig. 1B). The young *Ljglb3-2* plants had fewer nodules than the WT (Fig. 1A) but this difference was no longer present in the older plants; however, the nodules of line *30108411* weighed less than those of the WT in older plants (Fig. 1B).

The phenotypes of nodulated plants were also examined at flowering and fruiting (Table 1). Compared with the WT, all the mutant lines showed a delay of 1 or 2 weeks until they reached the maximum numbers of flowers and pods, with the exception of *Ljglb1-1*, which was delayed by 1 week in reaching the flowering peak but not the pod peak. The two class 1 Glb mutants showed contrasting differences in pod number, which increased by 63% in *Ljglb1-1* and decreased by 31% in *Ljglb1-2* compared with the WT. In contrast, the *Ljglb2-1* plants showed no differences in the number of flowers and pods. The *Ljglb3-2* mutant lines had more flowers and pods than the WT plants. In addition, the *Ljglb1-1*, *Ljglb2-1*, and *Ljglb3-2* (*30086451*) mutants produced smaller pods and fewer seeds than the WT.

**Table 1.** Flowering and fruiting phenotypes of nodulated *Lotus japonicus* plants of the wild-type and *Ljglb1-1, Ljglb1-2*, *Ljglb2-1*, and *Ljglb3-2* mutants.

Parameter	WT (4 + 4)	Ljglb1-1	Ljglb1-2	Ljglb2-1	WT (5 + 3)	Ljglb3-2 (30086451)	Ljglb3-2 (30108411)
Flowering peak time^a^	16	17	17	18	16	18	18
Flower number^b^	8.8±1.6	12.4±2.0	8.2±1.4	9.8±1.5	8.2±1.6	22.1±3.8*	13.0±2.5*
Pod peak time^a^	21	21	22	23	21	23	23
Pod number^b^	19.5±2.3	31.8±4.3*	13.5±1.6*	21.5±2.9	17.0±0.5	28.3±3.6*	22.7±4.8*
Pod length (cm)^c^	2.8±0.1	2.5±0.1*	2.8±0.1	2.5±0.1*	2.8±0.1	2.5±0.0*	2.9±0.1
Seed number per pod^c^	10.8±0.4	8.5±0.4*	11.9±0.7	8.9±0.4*	10.5±0.5	9.4±0.3*	10.7±0.8

^a^ Weeks from germination.

^b^ Value at the peak time. Data are means (±SE) of 3–9 plants (*Ljglb3-2*) or 15–35 plants (all other lines).

^c^ Data are means (±SE) of 20–80 (*Ljglb3-2*) or 50-100 (all other lines) pods from one to four sets of plants grown independently.

Data were obtained from one (*Ljglb3-2 30108411*), two (*Ljglb1-2*, *Ljglb3-2 300846451*), or four *(Ljglb1-1* and *Ljglb2-1*) sets of plants grown independently. WT (4+4) indicates the wild-type used for *Ljglb1-1*, *Ljglb1-2*, and *Ljglb2-1* (4 weeks in plates, 4 weeks in pots); WT (5+3) indicates the wild-type used for the *Ljglb3-2* lines (5 weeks in plates, 3 weeks in pots). Significant differences compared with the corresponding WT were determined using Student’s *t*-test: *P<0.05.

### LjGlb2-1 is also functional in non-nodulated plants and its deficiency causes alterations in the metabolome

**Fig. 2.**
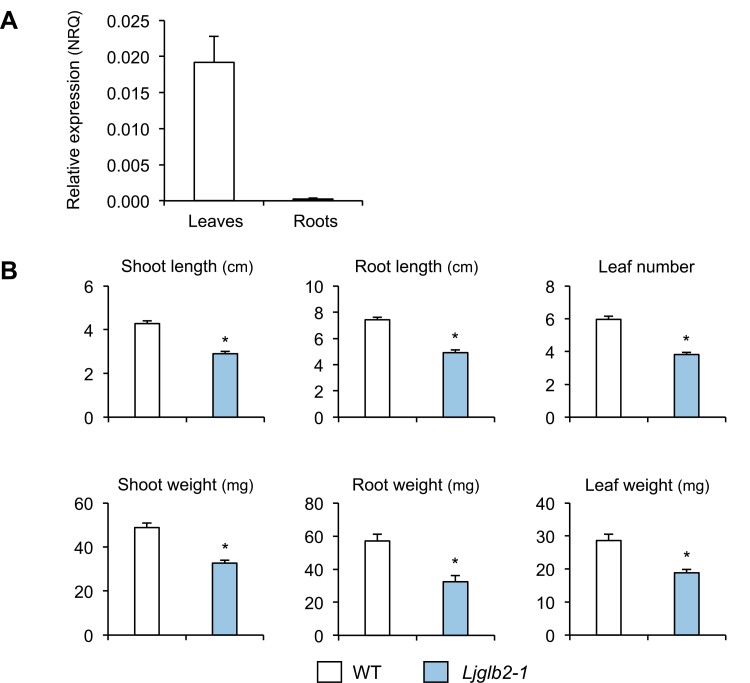
Expression of *LjGlb2-1* and growth phenotypes of non-nodulated *Lotus japonicus* plants. Measurements were taken on 21-d-old plants grown on Jensen plates supplemented with 1.5 mM NH_4_NO_3_. (A) Expression of *LjGlb2-1* in non-nodulated wild-type (WT) plants, presented as normalized relative quantity (NRQ). Transcript levels were normalized using the geometric means of *LjUbiquitin*, *LjATP synthase*, and *LjeIF4A* as reference genes. Data are means (±SE) of three biological replicates. (B) Phenotypes of the WT and mutant deficient in LjGlb2-1. Data are means (±SE) of 55–60 plants from four sets of plants grown independently. Significant differences between means were determined using Student’s *t*-test: *P<0.05.

**Fig. 3.**
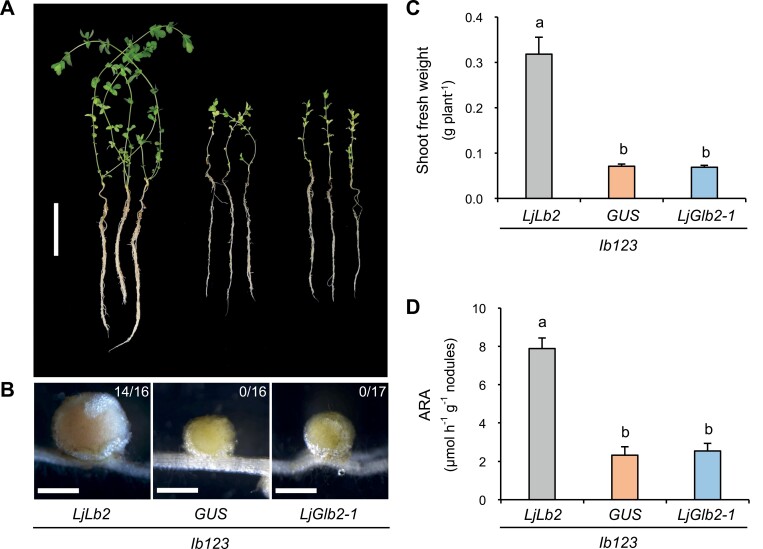
Complementation analysis of the *Lotus japonicus lb123* mutant by *LjGlb2-1*. Phenotypes of (A) whole plants and (B) nodules of hairy root transformed plants at 6 weeks post-inoculation. Roots of the *lb123* mutant were transformed with *Agrobacterium rhizogenes* strain LBA1334 harboring the *LjLb2* promoter coupled to either *LjLb2*, *GUS*, or *LjGlb2-1*. The numbers in (B) indicate the proportion of plants bearing pink nodules. Scale bars are (A) 5 cm and (B) 1 mm. (C) Shoot fresh weight per plant. (D) Acetylene reduction activity (ARA) expressed per nodule fresh weight. Data are means (±SE) of 16–17 plants. Different letters indicate significant differences between means as determined by Duncan’s multiple range test (*P*<0.05).

The hemoglobin LjGlb2-1 is particularly interesting because of its unusual features, as recently discussed by Larrainzar *et al.* (2020). LjGlb2-1 shows high homology with typical Lbs but has a hexacoordinate heme (see below) and its gene expression is localized not only in nodules but also in the apex and vascular bundles of the roots (Bustos-Sanmamed *et al.*, 2011). This prompted us to focus the rest of our study on LjGlb2-1. First, we quantified the expression of *LjGlb2-1* in WT plants grown for 21 d in Jensen plates supplemented with 1.5 mM NH_4_NO_3_. Under these non-nodulating conditions, the gene was expressed at a level ~80-fold lower in the roots than in the leaves (Fig. 2A). This finding was interesting because a previous study of nodulated plants grown in hydroponics had shown that *LjGlb2-1* expression was highest in nodules, moderately high in roots, and very low in leaves (Bustos-Sanmamed *et al.*, 2011). Next, we phenotyped the *Ljglb2-1* mutant plants under the same non-nodulating conditions that were used for transcript quantification and found that they showed decreases of 32–43% in all the measured growth parameters compared to the WT plants (Fig. 2B). We then carried out a complementation study by producing hairy roots of the *lb123* mutant, which is devoid of the three LjLbs (Wang *et al.*, 2019). The roots of this mutant were transformed via *A. rhizogenes* with constructs bearing the *LjGlb2-1* gene under the control of the *LjLb2* promoter. As controls, we used constructs in which the *LjLb2* promoter was driving the *LjLb2* and *GUS* genes. The results indicated that *LjGlb2-1* did not complement the *lb123* mutant, based on the decreases in plant length and weight, the white color of the nodules, and the very low acetylene reduction activity compared with the *lb123* roots transformed with the *LjLb2* gene (Fig. 3). Consequently, the results support a hypothesis that LjGlb2-1 is not a typical Lb but is instead a hemoglobin with at least some functions that are non-symbiotic.

**Fig. 4.**
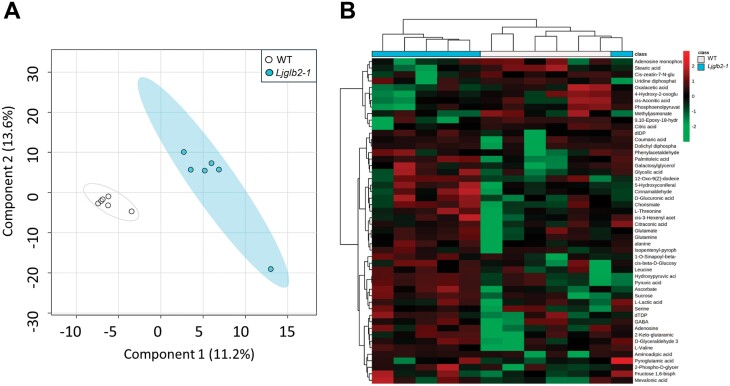
Metabolomic characterization of the *Lotus japonicus Ljglb2-1* mutant. Metabolites extracted from leaves of 28-d-old wild-type (WT) and *Ljglb2-1* plants were determined using flow-infusion electrospray–high resolution mass spectroscopy. (A) The derived matrix (Supplementary Dataset S1) was assessed by partial least-squares discriminant analysis. The major sources of variation were identified based on one-way ANOVA correcting for false-discovery rates and using a significance level of *P*<0.05. The discriminatory *m*/*z* were identified based on accurate mass (5 ppm resolution) and correlations between multiple adducts of the targeted metabolite as indicated by DIMEdb (https://dimedb.ibers.aber.ac.uk). (B) Heatmap comparing the levels of the identified metabolites in WT and *Ljglb2-1* plants.

**Fig. 5.**
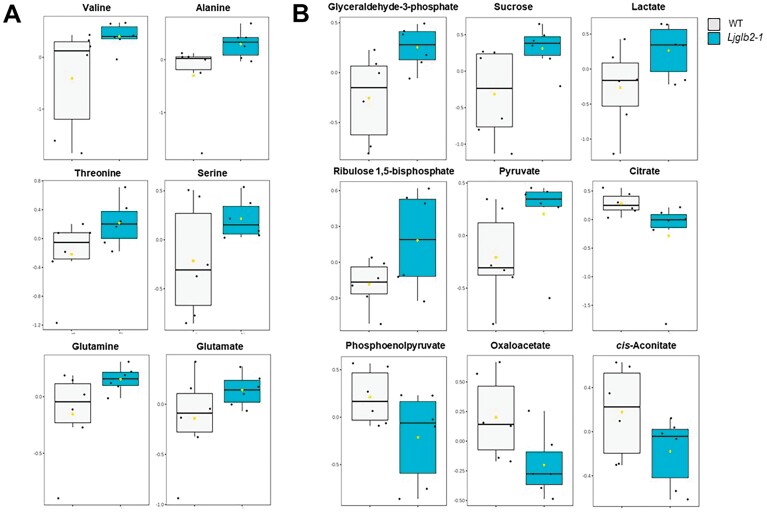
Comparison of metabolites discriminating between the *Lotus japonicus* wild-type (WT) and *Ljglb2-1* mutant plants. Box-and-whisker plots of (A) amino acids and (B) metabolites associated with bioenergy that significantly discriminated between the two genotypes (*P*<0.05).

**Fig. 6.**
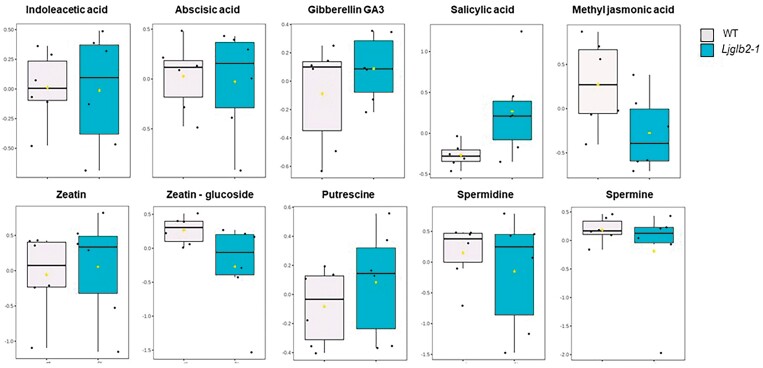
Comparison of hormone levels between the *Lotus japonicus* wild-type (WT) and *Ljglb2-1* mutant plants. The matrix derived following flow-infusion electrospray–high resolution mass spectroscopy of extracts from the two genotypes (Supplementary Dataset S1) was interrogated to identify *m*/*z* linked to hormones. The hormones were identified based on accurate mass (5 ppm resolution) and correlations between multiple adducts as indicated by DIMEdb (https://dimedb.ibers.aber.ac.uk). The data linked to each identified hormone were retrieved and compared using box-and-whisker plots. Only salicylic acid, methyl jasmonic acid, and zeatin–glucoside showed significant differences between the two genotypes, as indicated by one-way ANOVA (*P*<0.05).

To further investigate the phenotype of the *Ljglb2-1* mutant at the biochemical level, we performed a metabolomics study in leaves of non-nodulated plants, where expression of *LjGlb2-1* was maximal in the WT plants (Fig. 2A). This identified 1283 *m*/*z* features within the metabolite profile (Supplementary Dataset S1). Principal component analysis of these features indicated a clear separation between the WT and *Ljglb2-1* plant metabolomes (Fig. 4A). The major sources of variation were identified based on one-way ANOVA, correcting for false discovery rates and using a significance level of *P*<0.05. Compounds found to show the highest variation between the two genotypes were identified using metabolite databases, and the relative magnitudes of these variations are indicated in the heatmap in Fig. 4B. The results suggested that most of the discriminatory metabolites were found at higher levels in *Ljglb2-1*. Considering all of the metabolites, it is worth noting the changes in the contents of several amino acids and intermediates of the tricarboxylic acid and glycolytic pathways. Thus, the leaves of *Ljglb2-1* plants contained higher levels of valine, alanine, threonine, serine, glutamine, and glutamate compared with the WT (Fig. 5A). The mutant also had increased levels of sucrose, glyceraldehyde-3-phosphate, lactate, ribulose 1,5-bisphosphate, and pyruvate, and lower levels of citrate, *cis*-aconitate, oxaloacetate, and phosphoenolpyruvate (Fig. 5B). Metabolite profiling also revealed alterations in the hormone levels in the leaves of mutant plants, including an increase in salicylic acid (SA) and a decrease in methyl jasmonic acid (MeJA) (Fig. 6). It was also possible to identify *m/z* features linked to hormones in our metabolomic data that were not shown to be significantly different in our statistical pipeline. Thus, we were able to describe the levels of indoleacetic acid, abscisic acid, zeatin (and its glucoside), and polyamines in our plants. However, none of them appeared to change significantly in *Ljglb2-1* compared to WT plants, except the content of zeatin-glucoside, which decreased in the mutant plants (Fig. 6).

### LjGlb2-1 and MtLb3 are both located in roots and nodules and show different biochemical features

The functions of hemoglobins are associated to their locations in plant tissues and their reactivities toward physiological ligands. This prompted us to characterize LjGlb2-1. The protein shares high sequence identity with MtLb3 (Medtr1g090810), and they cluster together and clearly away from other Lbs and Glbs of the model legumes (Larrainzar *et al.*, 2020). These observations and the anomalous features of LjGlb2-1 led us to compare the expression patterns of *LjGlb2-1* and *MtLb3* in nodulated roots and the reactivities of their proteins to ascertain if they might be functional homologs. Because *LjGlb2-1* activity has previously been localized in nodules and roots using promoter–*GUS* fusions (Bustos-Sanmamed *et al.*, 2011), we used the same procedure for *MtLb3*. Our results showed that the *MtLb3* promoter was active in the apex and vascular bundles of the primary and secondary roots (Supplementary Fig. S4). In nodules at ~2 weeks post-infection, *MtLb3* activity was predominantly found in the apex (zones I + II), and after a further 2 weeks it was also detected in the vascular bundles and, to a lesser extent, in zone III. The expression pattern of *MtLb3* in the roots was therefore similar to that of *LjGlb2-1* observed by Bustos-Sanmamed *et al.* (2011).

We then set out to compare the two proteins, together with a MtLb3 mutant in which its single Cys was replaced by Ser (MtLb3–C135S). Unfortunately, we could not produce the corresponding derivative of LjGlb2-1 (LjGlb2-1–C65S) in sufficient quantities for characterization, which suggested that the Cys residue is critical for protein stability (Calvo-Begueria *et al.*, 2017). Having produced purified proteins, we first obtained the UV-visible spectra of the 3+ and 2+ forms, as well as the spectra of the proteins bound to the heme ligands O_2_, NO, CO, and cyanide (Supplementary Figs S1–S3). In this study, LjGlb2-1 had a Strep-tag instead of a His-tag (Sainz *et al.*, 2013), confirming that the protein is hexacoordinate in the 3+ form (Soret at 410 nm, band at 535 nm, and shoulder at 562 nm). The 3+ protein produced the typical cyano complex with cyanide, whereas the 2+ protein was mostly pentacoordinate (Soret at 427 nm and β band at 558 nm) and yielded the expected complexes with O_2_, CO, and NO (Supplementary Fig. S1). However, a small proportion of hexacoordinate protein was also detectable in the 2+ form according to the shoulder observed at ~534 nm. The spectra of MtLb3 (Supplementary Fig. S2) and MtLb3–C135S (Supplementary Fig. S3) were virtually identical (±2 nm), showing pentacoordination in the 3+ form (bands at 528 and 625 nm, shoulder at 564 nm) and 2+ form (β band at 559 nm).

**Fig. 7.**
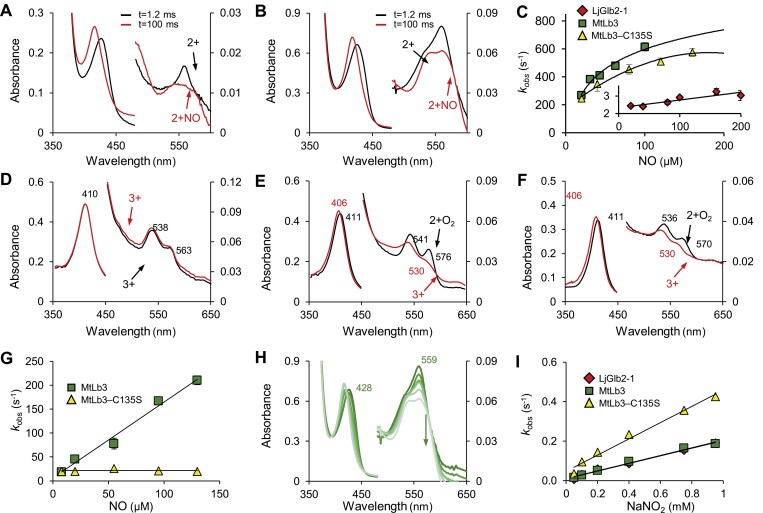
Reactivity of LjGlb2-1 and MtLb3 with NO and nitrite. (A, B) UV-visible spectra of the reaction of 20 μM NO with 2.5 μM of the 2+ forms of (A) LjGlb2-1 and (B) MtLb3. Similar spectra were obtained for MtLb3–C135S. Spectra were taken at 1.2 ms and 100 ms after mixing. (C) The panel and inset show plots of observed pseudo-first-order rate constants (*k*_obs_) at pH 7.0 and 20 °C versus NO concentration. The *k*_obs_ were calculated from fitting the time-course to single-exponential functions. The NO–binding rate constant (*k*_NO_) of MtLb3 and MtLb3–C135S were calculated by fitting to the hyperbolic curves. The *k*_NO_ of LjGlb2-1 was independent of NO concentration. (D–F) UV-visible spectra of the NOD reaction after mixing 20 μM NO with 2.5 μM of the 2+O_2_ forms of (D) LjGlb2-1, (E) MtLb3, and (F) MtLb3–C135S. Spectra were taken at 1.2 ms (black) and 100 ms (red) after mixing. Note that in (D) the reaction of LjGlb2-1 was already complete at 100 ms, producing the 3+ protein. (G) Kinetic analysis of the NOD reaction between NO and the 2+O_2_ forms of MtLb3 and MtLb3–C135S; for LjGlb2-1 the reaction was so fast that it could not be represented. The plots are the observed pseudo-first-order rate constants (*k*_obs_) at pH 7.0 and 10 °C versus NO concentration. The second-order rate constant (*k*_NOD_) was obtained from the linear fit to the data. (H) Serial spectra illustrating the NiR activity of MtLb3. Similar spectra were observed for LjGlb2-1 and MtLb3–C135S. The arrow indicates the disappearance of the 2+ form (maxima at 428 nm and 559 nm) concomitant to the appearance of the 2+NO form (maxima at 417, 542, and 567 nm; for clarity these are not indicated). (I) Kinetic analysis of the NiR reactions of LjGlb2-1, MtLb3, and MtLb3–C135S. The plots are the observed pseudo-first-order rate constants (*k*_obs_) at pH 7.0 and 25 °C versus NO_2_^–^ concentration. The second-order rate constant (*k*_NiR_) was obtained from the linear fit to the data.

Next, we compared LjGlb2-1 and MtLb3 by measuring their reactivity toward NO, because this is a key signal molecule and the kinetics may give insights into the protein functions. To study the kinetics of NO binding, the 2+ hemoglobins were mixed with proli-NONOate and the appearance of the nitrosyl complex (2+NO) was followed under anaerobic conditions by stopped-flow spectroscopy (Fig. 7A, B). The reaction was fast for all three proteins, but the 2+ form was still visible after the dead-time of the instrument (1.2 ms) and the rates of formation of the 2+NO hemoglobins could be measured. There were clear differences between the proteins. The time-course of MtLb3 fitted a double-exponential function whereas that of MtLb3–C135S fitted a single-exponential function, which suggested that a second, slower reaction occurred during the last step of the NO binding to MtLb3. The observed rate constant (*k*_obs_) exhibited a hyperbolic relationship with NO concentration (Fig. 7C). The initial change in *k*_obs_ appeared to be at least 1×10^7^ M^–1^ s^–1^ for both MtLb3 and MtLb3–C135S, and the rate constants approached maximum values of ~860 s^–1^ and ~695 s^–1^, respectively. These values represent the pseudo-first-order rate constants that reflect the limitation of the rate of access of NO to the heme. In many cases they are the His off rates from the iron, but here they probably reflect the rate of movement of distal heme-pocket residues that sterically hinder the binding of NO. The NO-binding kinetics of LjGlb2-1 was slower than that of MtLb3 and was independent of NO concentration, with an estimated NO-binding rate constant (*k*_NO_) of 2.5 s^–1^ (Fig. 7C, inset). This indicated that the entry of NO into the heme cavity of LjGlb2-1 was somewhat limited by the presence of the distal His. This finding was unexpected because, as indicated above, the 2+ protein showed a mainly pentacoordinate spectrum.

Because all three hemoglobins were able to scavenge NO *in vitro*, we measured their NOD activities, which also allowed us to determine whether the Cys residue of MtLb3 plays a role in NO scavenging. To this end, the 2+O_2_ proteins were mixed with a solution of NO and the reaction kinetics were monitored by stopped-flow spectroscopy. The first spectrum of LjGlb2-1 (taken at 1.2 ms) was almost the same as that of a 3+ protein, indicating that the reaction was already complete (Fig. 7D). It can therefore be concluded that the NOD rate constant (*k*_NOD_) must be >250 s^–1^ even at very low NO concentrations (5 μM), yielding a second-order rate constant of >5×10^7^ M^–1^ s^–1^. In sharp contrast, the initial spectra (again taken at 1.2 ms) of MtLb3 (Fig. 7E) and MtLb3–C135S (Fig. 7F) were typical of 2+O_2_ hemoglobins, whereas the spectra taken at 100 ms corresponded to 3+ pentacoordinate proteins. This meant that the reactions were slow enough to be observed even at high NO concentrations (200 µM). The two proteins showed important kinetic differences: MtLb3 showed kinetics that fitted to a second-order rate (*k*_NOD_=1.5×10^6^ M^–1^ s^–1^), whereas MtLb3–C135S showed a reaction rate ~40-fold slower at low NO concentrations (5 μM) and its NOD reaction was independent of NO concentration (*k*_NOD_=20 s^–1^) (Fig. 7G).

We also assayed NiR reactions with 0.05–1.0 mM NO_2_^–^ (at pH 7.0 and 25 °C) in the presence of excess dithionite. Under these conditions, the 2+ hemoglobin reduced NO_2_^–^ to NO and generated only the 2+NO complex because the excess dithionite avoided the production of 2+O_2_ hemoglobin and quickly reduced the resulting 3+ form (Fig. 7H). The observed pseudo-first-order rate constant (*k*_obs_) for NiR activity was plotted against NaNO_2_ concentration and the second-order rate constant (*k*_NiR_) was calculated from the slopes (Fig. 7I). The *k*_NiR_ values of LjGlb2-1 and MtLb3 were ~200 M^–1^ s^–1^, whereas that of MtLb3–C135S was 428 M^–1^ s^–1^. This indicated that the Cys residue of MtLb3 was important for NiR activity, as was also found for NOD. The observation of virtually identical *k*_NiR_ values of LjGlb2-1 and MtLb3 contrasted with the different responses of the proteins toward NO, as reflected by their *k*_NO_ and *k*_NOD_ values.

## Discussion

Hemoglobins are widespread in plant tissues and display a considerable diversity of structures and probably of functions (see reviews by Garrocho-Villegas *et al.*, 2007; Hoy and Hargrove, 2008; Hill, 2012; Becana *et al.*, 2020). However, the effects of hemoglobin deficiencies on plant development have seldom been studied, with the notable exception of Arabidopsis. In the present study, we used mutant lines of *L. japonicus* to investigate the effects of several Glb deficiencies on plants at the vegetative and reproductive stages. The use of stable knockout mutants rather than antisense lines in hairy roots allowed us to study nodulated plants until flower and pod formation, and hence to uncover phenotypic alterations at late stages in the life cycle. To date, only Lbs (Ott *et al.*, 2005; Wang *et al.*, 2019) and one class 1 Glb (Fukudome *et al.*, 2016; Berger *et al.*, 2020) of *L. japonicus* and *M. truncatula* have been examined using transgenic approaches, but these studies were focused on plant nodulation. Our results indicated that a deficiency of LjGlb1-1, LjGlb2-1, or LjGlb3-2 negatively affected both nodule and plant development, with significant reductions of shoot and root size and of leaf and nodule number compared with the WT (Fig. 1). Arabidopsis possesses a single class 1 Glb (AtGlb1), and a previous study has shown that plants lacking this display aberrant development, with twisted leaves and enlarged leaf hydathodes, although these effects are not seen in plants lacking the class 2 Glb (AtGlb2; Hebelstrup *et al.*, 2006). In our study, it was notable that the Glb deficiencies delayed flowering and affected pod-set, with the mutants showing variable effects. Compared with the WT, the flowering peak was delayed by 1 week in *Ljglb1-1* and *Ljglb1-2*, and by 2 weeks in *Ljglb2-1* and *Ljglb3-2* (Table 1). This could be related to the observation that AtGlb1-deficient Arabidopsis plants have a delay in bolting that ultimately results in formation of aerial rosettes (Hebelstrup and Jensen, 2008). It could be argued that the delay in flowering and fruiting of *L. japonicus* might be a direct consequence of retarded vegetative growth; however, this hypothesis was not supported by the differences between the mutant phenotypes, such as an increase in the number of flowers in *Ljglb3-2* and a decrease in the pod length in *Ljglb1-1* and *Ljglb2-1* (Table 1). Furthermore, the *Ljglb1-2* plants had no differences in phenotype in the vegetative period (Fig. 1) but the flowering peak was delayed and the mutant produced fewer pods than the WT (Table 1). Overall, our results indicated that Glbs were involved not only in vegetative growth but also in the flowering and fruiting of plants. Because all of the mutants showed distinct phenotypes, we conclude that the three classes of Glbs have non-redundant functions and are all needed for optimal plant development.

Another intriguing finding of our study was the detection of a distinctive growth (Fig. 2) and metabolic (Figs 4–6) phenotype in plants deficient in LjGlb2-1 that had been fed on NH_4_NO_3_. The mutant plants were smaller and their leaves contained higher levels of sucrose and intermediates of glycolysis and the tricarboxylic acid cycle, which was suggestive of mobilization of carbon assimilates, apparently tending towards a fermentative over a bioenergetic oxidative route. The *Ljglb2-1* mutant also had more SA and less MeJA in the leaves than the WT (Fig. 6). These hormones have been often considered to act antagonistically to tailor defenses against particular pathogens. Thus, Arabidopsis plants lacking AtGlb1 show an accumulation of SA and a decrease of JA in the leaves when challenged with the pathogens *Pseudomonas syringae* and *Botrytis cinerea*, respectively, and these effects are linked to a modulation of NO by AtGlb1 (Mur *et al.*, 2012). This might also be the case for LjGlb2-1 because our *in vitro* studies showed that the protein had a very high NOD activity (>250 s^–1^; Fig. 7D). Consequently, its deficiency could disturb NO homeostasis, which is likely to impact on SA and JA biosynthesis (Mur *et al.*, 2013). These hormones are also likely to influence whole-plant physiology beyond defense. High SA induces or accelerates flowering (Jin *et al.*, 2008) and low SA can increase growth (Abreu and Munné-Bosch, 2009). In addition, jasmonates decrease plant growth by suppressing the mitotic CycB1;2 (Zhang and Turner, 2008). Hence, the relative levels of SA and JA in the *Ljglb2-1* mutant plants do not explain their smaller size compared with the WT. A possible explanation to reconcile all these observations is that the growth alterations in the *Ljglb2-1* mutant reflected a general defect in redox and/or NO homeostasis, which would in turn affect the levels of both hormones. Further studies will be necessary to determine whether the mechanism involves the important regulator of pathogen defense *NONEXPRESSOR OF PATHOGENESIS-RELATED GENES 1* (*NPR1*), which mediates the crosstalk between the SA and JA/ethylene signaling pathways (Backer *et al.*, 2019). Our metabolomic study also examined other hormones, but we were unable to detect significant changes in the leaf contents of auxin, polyamines, and gibberellin GA_3_ in the mutant plants and hence they were probably not involved in the reduced growth.

The finding of significant *LjGlb2-1* expression in the leaves of non-nodulated WT plants (Fig. 2A), together with the phenotype of the mutant plants, indicated that this hemoglobin performs at least some functions that are non-symbiotic and that it is not a Lb, in contrast to previous reports (Berger *et al.*, 2020). Several pieces of evidence support this conclusion: LjGlb2-1 in the 3+ form was found to be hexacoordinate (Supplementary Fig. S1); it did not complement the *lb123* mutant (Fig. 3); and it is known to be present at much lower concentrations than Lbs because *lb123* nodules are completely white (Wang *et al.*, 2019). Nevertheless, LjGlb2-1 also shows some discrepancies with the few class 2 Glbs that have been characterized to date: class 2 Glbs show strong hexacoordination in the 2+ form (Smagghe *et al.*, 2009) and their NiR rate constants (*k*_NiR_) are ~20-fold lower than those that we found for LjGlb2-1 (Sturms *et al.*, 2011; Tiso *et al.*, 2012; Leiva Eriksson *et al.*, 2019). Because class 2 Glbs are the evolutionary precursors of most Lbs (Hoy *et al.*, 2007), our future aim is to test the intriguing hypothesis that LjGlb2-1 is an intermediate between the two types of plant hemoglobins.

The LjGlb2-1 and MtLb3 proteins share high sequence homology (66% identity and 81% similarity) and have very high and almost identical *k*_NiR_ values (~200 M^–1^ s^–1^; Fig. 7I). Indeed, these *k*_NiR_ values are only exceeded by those reported very recently for two extremely rare class 1 Glbs encoded by *MtGlb1-2* (Villar *et al.*, 2020). The two proteins therefore have the potential to generate NO under hypoxia and this biochemical property might be important in nodules in the presence of NO_3_^–^, which increases the O_2_ diffusion resistance of nodules and hence leads to more hypoxic conditions inside the N_2_-fixing zone (Minchin *et al.*, 1989). However, the LjGlb2-1 and MtLb3 proteins also showed some notable differences. MtLb3 was found to be pentacoordinate in the 3+ and 2+ forms (Supplementary Fig. S2), much like Lbs, but whereas its NO-binding rate constant (*k*_NO_) was comparable to those of known Lbs, it was faster than that of LjGlb2-1 (Fig. 7). In contrast, MtLb3 had a much slower NOD rate constant (*k*_NOD_) than that of LjGlb2-1. These features, together with its very low expression in nodules relative to typical MtLbs such as MtLb1 (Medtr1g011540) and MtLb6 (Medtr5g066070) (Gallusci *et al.*, 1991; Larrainzar *et al.*, 2020), support our suggestion that MtLb3 is an atypical Lb and is not involved *in vivo* in NO scavenging through the NOD reaction.

In conclusion, this study shows that deficiency in any of the three Glb classes in *L. japonicus* decreases vegetative growth and delays the flowering and fruiting of nodulated plants. The effects vary according to which *Glb* gene is knocked out, indicating that they have non-redundant functions and underlining the fact that all three classes (and even the two members of class 1) are required for optimal plant development. The deficiency of LjGlb2-1 in plants grown on combined nitrogen sources also results in retarded growth and changes in the leaf metabolome that are linked to amino acid processing, the fermentative and respiratory pathways, and the balance between hormones. Notably, the mutant plants showed an increase of SA and a decrease in MeJA in the leaves, indicating that LjGlb2-1 is involved in the crosstalk with the signaling pathways of these hormones during plant development. Finally, we conclude that LjGlb2-1 is not a Lb but an unusual class 2 Glb, based on the expression of *LjGlb2-1* in the leaves of non-nodulated plants, the alterations of the reproductive growth of the *Ljglb2-1* mutant grown under nodulating conditions, the growth and biochemical phenotype of the *Ljglb2-1* mutant fed on NH_4_NO_3_, and the heme coordination and reactivity of the protein toward NO.

## Supplementary data

The following supplementary data are available at *JXB* online.

Table S1. Primers used for qRT-PCR analyses.

Table S2. Details of the hemoglobin mutant lines used in the study.

Dataset S1. Mass-ions (*m*/*z*) obtained following flow-infusion electrospray–high resolution mass spectroscopy based on using an Exactive Plus Orbitrap MS, with each ion given as a percentage of the total ion count.

Fig. S1. UV-visible spectra of the 3+ and 2+ forms of LjGlb2-1 and their representative complexes.

Fig. S2. UV-visible spectra of the 3+ and 2+ forms of MtLb3 and their representative complexes.

Fig. S3. UV-visible spectra of the 3+ and 2+ forms of MtLb3–C135S and their representative complexes.

Fig. S4. Localization of promoter activity of *MtLb3* in *Medicago truncatula* using a *GUS* reporter gene.

erab376_suppl_Supplementary_Materials_S1

erab376_suppl_Supplementary_Materials_S2

## Data Availability

All data supporting the findings of this study are available within the paper and within its supplementary materials published online.
