# Supplementary material for: Three classes of hemoglobins are required for optimal vegetative and reproductive growth of Lotus japonicus: genetic and biochemical characterization of LjGlb2-1
Source: J Exp Bot. 2021 Aug 13;72(22):7778–91. doi: 10.1093/jxb/erab376 (PMC8664582; doi:10.1093/jxb/erab376)
Supplement: erab376_suppl_Supplementary_Materials_S1 [file erab376_suppl_supplementary_materials_s1.pdf]

Table S1. Primers used for qRT-PCR analyses

Gene	Forward primer (5´→3´)	Reverse primer (5´→3´)	Efficiency (%)
LjUbiquitin	TTCACCTTGTGCTCCGTCTTC	AACAACAGCACACACAGACAA	93.6
LjATP synthase	AACACCACTCTCGATCATTTCTCTG	CAATGTCGCCAAGGCCCATGGTG	92.9
LjelF4A	AGAGGGTTTAAAGATCAAAT	ATGTCAATTCATCACGTTTT	92.1
LjGlb1-1	TTGAGGTTACAAAGTTTGCACTAC	TGCATTCTTCATCTCTGGTGAC	90.3
LjGlb1-2	CAGTGCCATCATAGCTGAAA	TATTGAAACTGAGAGCAAAGGG	99.3
LjGlb2-1	CTCAGCCCTTCAACTAAGAG	CTTTAAGCACCAGGAAATGGG	97.4
LjGlb3-2	GCAACAAGCATTAGACAGTACTC	TTCTTTAGCTCATTTCCAGCC	97.6

Table S2 Details of the hemoglobin mutant lines used in the study

**Gene**	**Gene ID**[a]	**Line**	**Insertion**	**mRNA (%)**[b]
LjGlb1-1	LotjaGi3g1v0504500	*30096642*	Exon 1 (5'-UTR)	0.9
LjGlb1-2	LotjaGi3g1v0504600	*P0494*	Exon 4	7.4
LjGlb2-1	LotjaGi5g1v0253250	*30015049*	Exon 2	6.6
LjGlb3-2	LotjaGi1g1v0172000	*30086451*	Exon 1	1.7
		30108411	Exon 2	1.5

[a]Gene IDs are given according to the *Lotus japonicus* Gifu genome sequence (Kamal *et al.*, 2020).
[b]Transcript levels were normalized using *LjUbiquitin* as the reference gene and are expressed as percent of those of the wild type.

LjGlb2-1

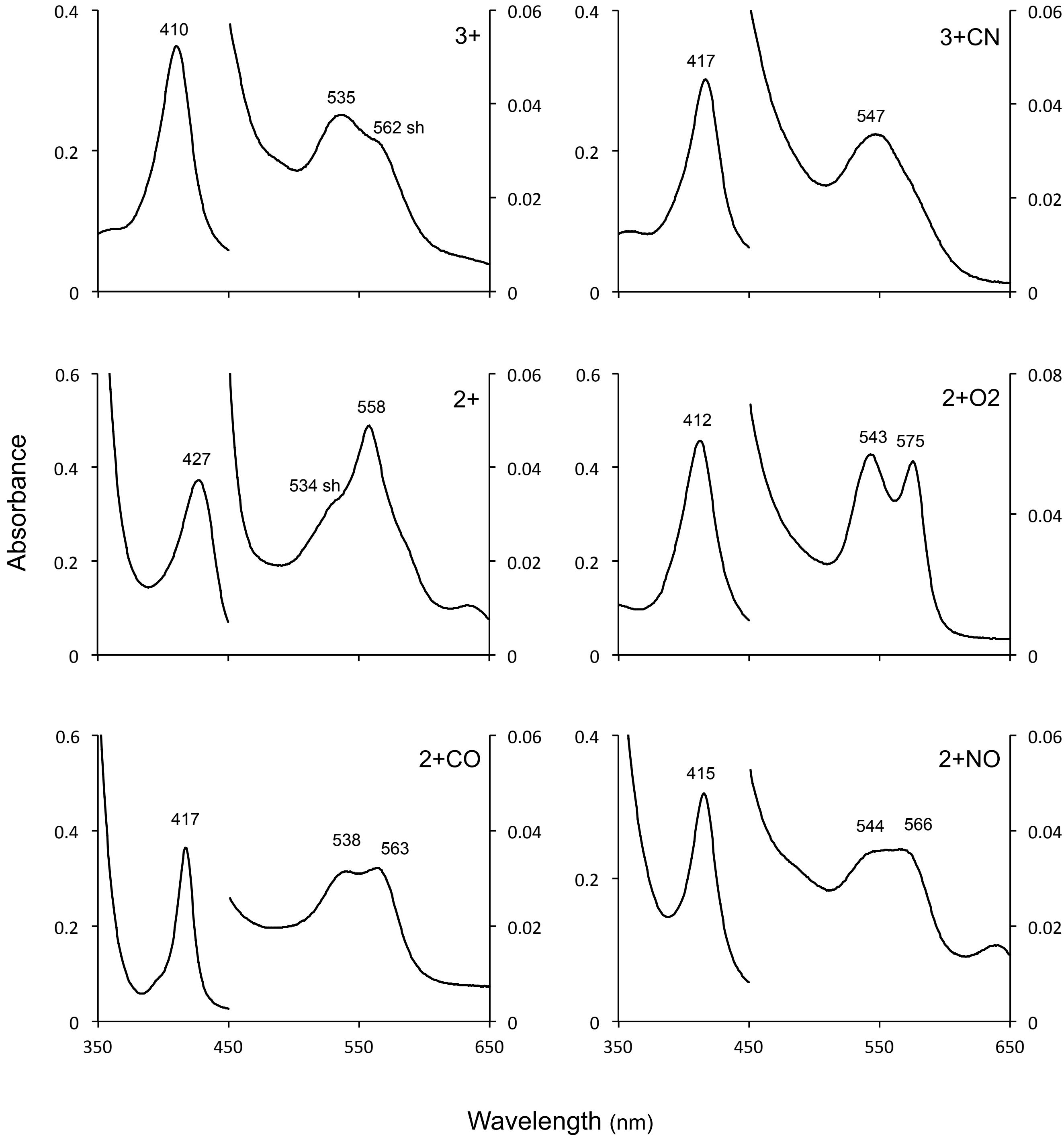

Fig. S1. UV-visible spectra of the 3+ and 2+ forms of LjGlb2-1 and their representative complexes. Spectra were taken with 24 µM protein in 50 mM potassium phosphate buffer (pH 7.0).

MtLb3

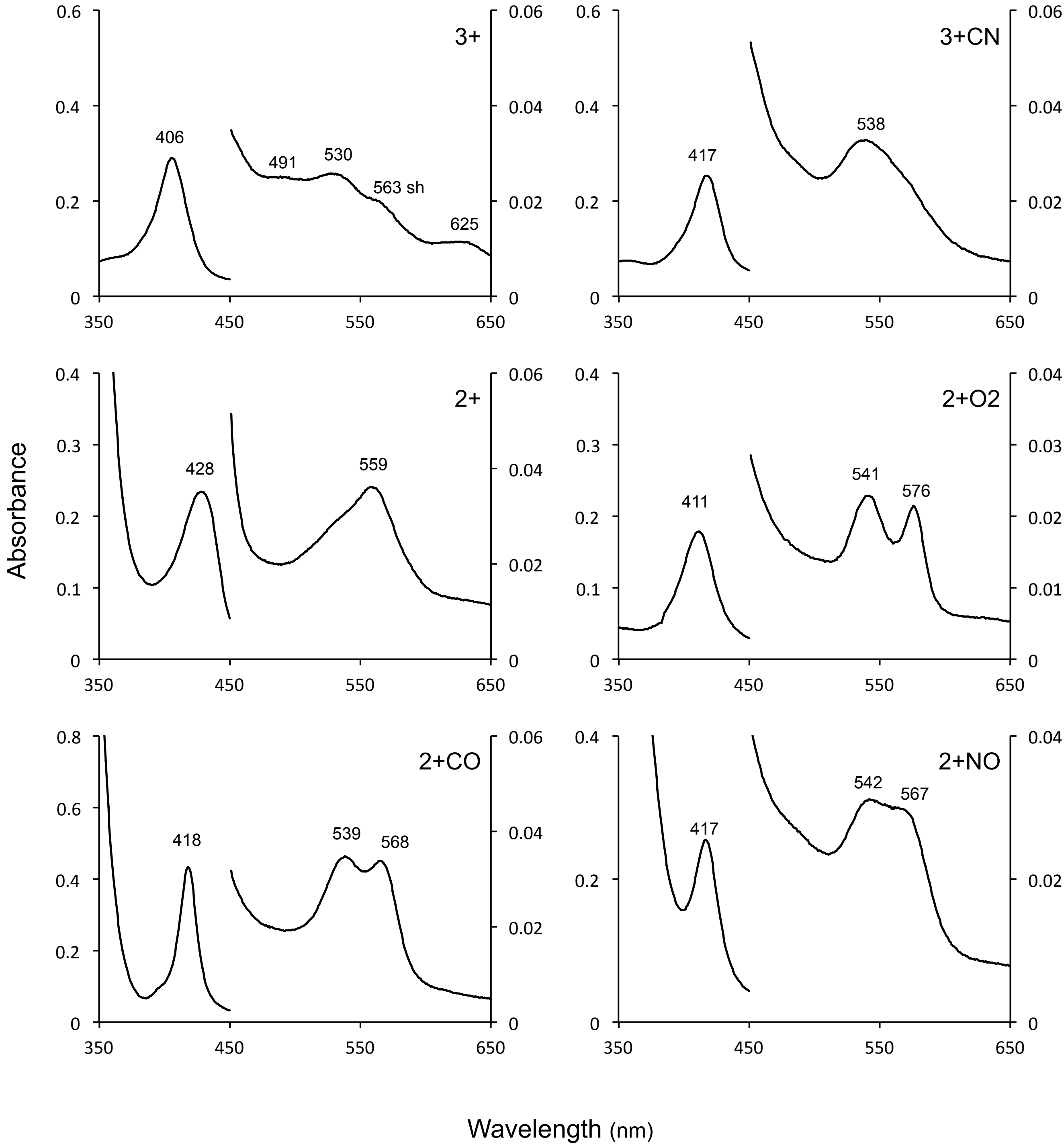

Fig. S2. UV-visible spectra of the 3+ and 2+ forms of MtLb3 and their representative complexes. Spectra were taken with 19 µM protein in 50 mM potassium phosphate buffer (pH 7.0).

MtLb3–C135S

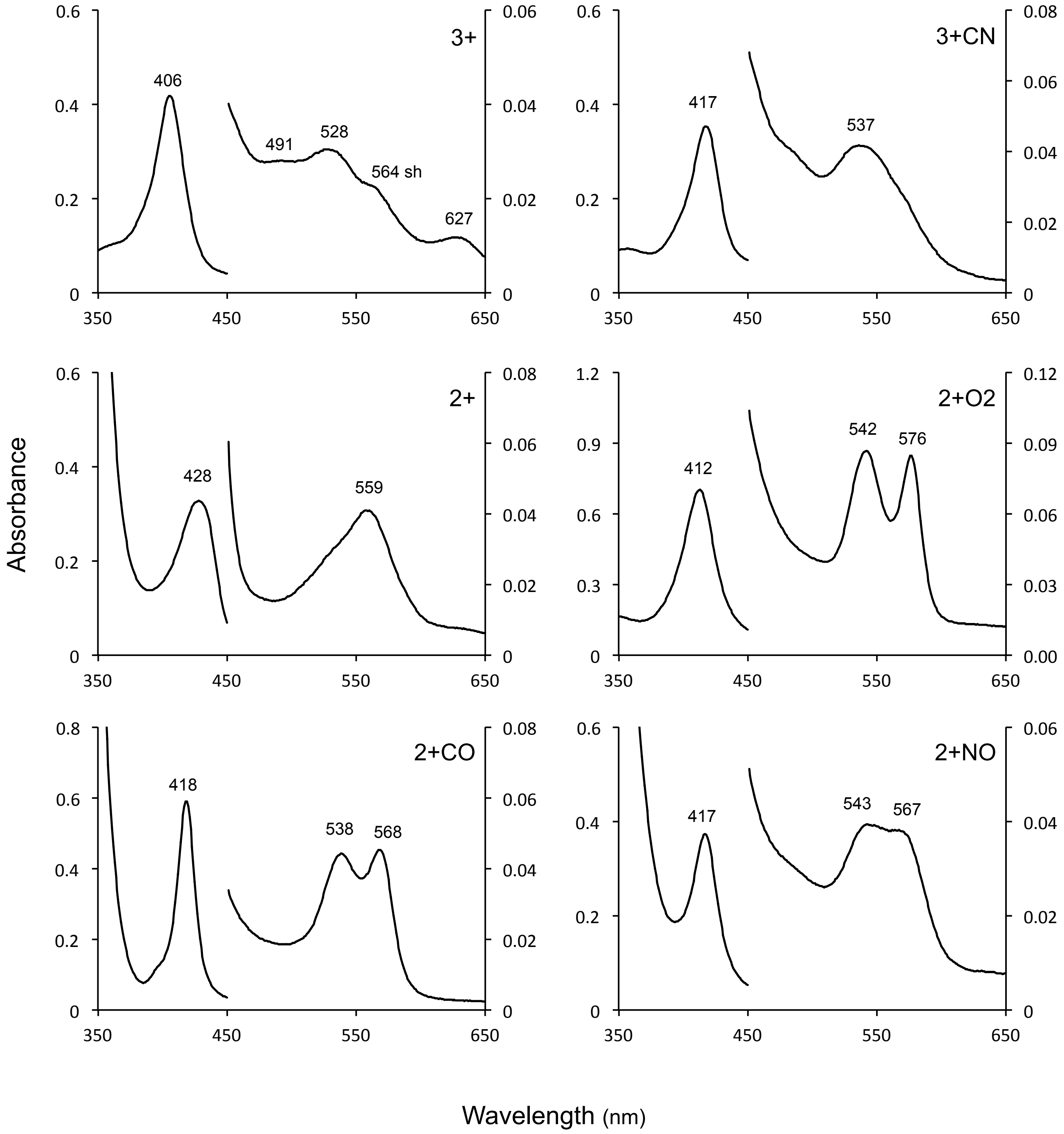

Fig. S3. UV-visible spectra of the 3+ and 2+ forms of MtLb3–C135S and their representative complexes. Spectra were taken with 28 µM protein in 50 mM potassium phosphate buffer (pH 7.0).

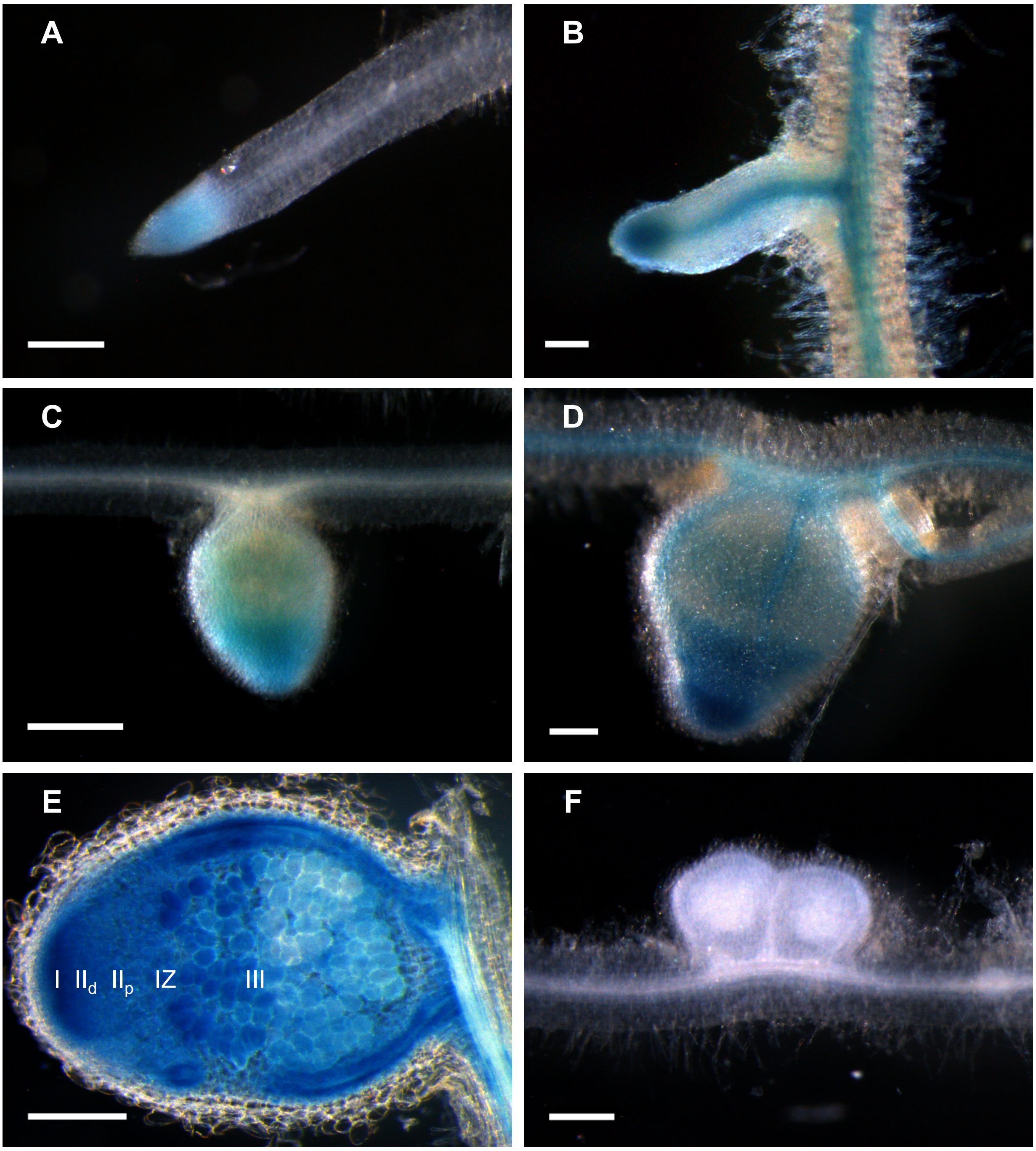

Fig. S4. Localization of promoter activity of *MtLb3* in *Medicago truncatula* using a *GUS* reporter gene. Expression was detected using X-Gluc and tissues were observed in a stereomicroscope using transmitted light. (A, B) GUS activity is observed in the apex and vascular bundles of the primary root and lateral roots. (C) In young nodules, ~2 weeks post-inoculation (wpi), the gene is predominantly expressed in the apex, encompassing zones I and II. (D, E) At later stages (4 wpi), expression is mainly visible in the apex, zones I and II, and vascular bundles, and to a lower extent, in zone III. The following zones are marked in this nodule for reference: zone I (meristem); zone II (infection), distal (d) and proximal (p); IZ (interzone); and zone III (fixation). (F) Nodulated roots of plants (2 wpi) transformed with the empty vector pBGWFS7 were used as a negative control. Images are representative of nodules from at least five plants with similar localization results. Scale bars, 200 μm.
