# Supplementary material for: Three classes of hemoglobins are required for optimal vegetative and reproductive growth of Lotus japonicus: genetic and biochemical characterization of LjGlb2-1
Source: J Exp Bot. 2021 Aug 13;72(22):7778–91. doi: 10.1093/jxb/erab376 (PMC8664582; doi:10.1093/jxb/erab376)
Supplement: erab376_suppl_Supplementary_Materials_S2 [file erab376_suppl_supplementary_materials_s2.pdf]

m.z,Ljglb2,Ljglb2,Ljglb2,Ljglb2,Ljglb2,Ljglb2,WT,WT,WT,WT,WT,WT
62.98026,1.06E+03,7.53E+02,6.85E+02,7.70E+02,5.91E+01,3.77E+02,1.43E+03,7.01E+02,1.68E+02,1.1
62.98395,1.34E+02,4.83E+02,6.85E+02,9.13E+02,5.91E+01,1.23E+03,1.35E+03,8.04E+02,8.58E+01,9.7
62.98478,3.17E+04,2.60E+04,3.22E+04,2.65E+04,1.25E+04,1.54E+04,2.92E+04,2.21E+04,1.84E+04,1.7
62.99194,8.87E+03,7.68E+03,1.13E+04,8.82E+03,3.79E+03,4.82E+03,9.35E+03,6.60E+03,6.87E+03,5.6
62.99291,7.58E+02,9.69E+02,7.63E+02,6.19E+02,2.00E+02,6.54E+01,4.98E+02,6.81E+02,7.44E+02,1.1
62.99677,7.45E+02,4.72E+02,1.05E+02,8.93E+02,2.00E+02,6.54E+01,1.04E+02,8.64E+02,4.30E+02,2.2
63.01103,1.16E+03,5.33E+02,1.45E+03,9.46E+02,2.00E+02,6.43E+02,1.14E+03,6.83E+02,8.30E+02,9.7
63.01841,9.93E+02,1.06E+03,1.23E+02,9.46E+02,2.00E+02,4.09E+02,1.12E+02,6.56E+02,1.11E+03,8.4
63.01875,9.93E+02,6.50E+02,1.23E+02,9.46E+02,2.00E+02,4.09E+02,1.12E+02,8.00E+02,4.03E+02,3.2
63.01891,9.93E+02,6.50E+02,1.23E+02,7.08E+02,2.00E+02,4.09E+02,1.12E+02,9.35E+02,3.58E+02,3.2
63.02043,1.10E+03,7.11E+02,1.17E+03,9.31E+02,2.00E+02,8.90E+02,9.20E+02,1.03E+03,6.84E+02,3.2
63.02228,8.76E+02,9.98E+02,1.31E+03,1.08E+03,2.00E+02,3.86E+02,9.62E+02,7.18E+02,7.89E+02,3.2
63.03519,8.91E+02,5.99E+02,9.43E+02,7.87E+02,2.00E+02,5.51E+02,1.03E+02,4.58E+02,3.61E+02,4.9
63.04325,9.38E+02,6.78E+02,1.23E+03,7.46E+02,4.11E+02,4.71E+02,8.83E+02,4.84E+02,5.18E+02,6.9
63.04375,9.38E+02,5.56E+02,1.28E+03,1.14E+03,4.11E+02,4.71E+02,8.83E+02,4.84E+02,5.18E+02,1.9
63.04996,6.00E+02,1.09E+03,1.37E+03,5.82E+02,4.11E+02,9.80E+02,9.32E+02,5.56E+02,7.68E+02,7.1
63.05265,1.23E+03,8.64E+02,1.21E+02,8.42E+02,4.11E+02,6.88E+02,7.29E+02,7.66E+02,5.24E+02,2.0
63.05828,9.12E+02,6.47E+02,8.82E+02,8.29E+02,4.11E+02,8.27E+02,1.02E+03,4.96E+02,1.05E+03,2.7
63.06491,9.58E+02,1.02E+03,9.65E+02,7.58E+02,4.11E+02,4.73E+02,1.02E+03,4.62E+02,1.13E+03,2.7
63.06567,9.58E+02,6.90E+02,9.65E+02,7.58E+02,4.11E+02,4.73E+02,9.46E+02,1.03E+03,6.36E+02,2.7
63.06609,9.58E+02,6.90E+02,9.65E+02,7.58E+02,4.11E+02,4.73E+02,9.09E+02,2.01E+02,6.83E+02,2.7
63.06676,1.04E+03,9.25E+02,9.65E+02,9.79E+02,4.11E+02,1.02E+03,1.04E+03,2.01E+02,8.27E+02,1.8
63.07105,9.27E+02,6.56E+02,1.32E+03,1.06E+03,4.11E+02,1.09E+03,7.60E+02,6.29E+02,7.52E+02,1.0
63.07407,1.41E+03,8.17E+02,1.10E+02,1.10E+03,4.11E+02,6.05E+02,7.60E+02,4.58E+02,5.73E+02,3.8
63.07549,9.57E+02,1.13E+03,1.46E+03,7.81E+02,4.11E+02,6.05E+02,7.60E+02,8.13E+02,9.74E+02,3.8
63.10087,7.34E+02,8.64E+02,7.98E+02,6.50E+02,4.11E+02,8.48E+02,9.36E+02,6.83E+02,4.87E+02,9.6
63.10423,8.09E+02,1.02E+03,8.22E+02,9.47E+02,4.11E+02,6.49E+02,7.47E+02,3.41E+02,2.21E+02,6.0
63.10591,1.21E+03,1.12E+03,8.68E+02,1.02E+03,4.11E+02,6.49E+02,7.47E+02,9.20E+02,3.21E+02,6.0
63.10827,6.84E+02,3.27E+02,8.05E+02,4.47E+02,4.11E+02,5.83E+02,9.60E+02,1.47E+03,5.79E+02,8.9
63.11449,8.45E+02,4.63E+02,1.25E+03,7.72E+02,4.11E+02,8.25E+02,1.11E+03,6.03E+02,1.01E+03,1.7
63.11768,8.11E+02,2.83E+02,1.01E+03,8.97E+02,4.11E+02,8.25E+02,1.02E+03,2.72E+02,3.63E+01,1.7
63.12021,9.51E+02,7.14E+02,8.33E+02,9.11E+02,4.11E+02,1.16E+03,1.07E+02,9.68E+02,3.10E+02,1.0
63.12054,5.07E+02,7.96E+02,8.33E+02,9.78E+02,4.11E+02,1.29E+02,8.68E+02,4.80E+02,3.10E+02,3.1
63.12206,1.16E+03,4.64E+02,8.33E+02,7.92E+02,4.11E+02,1.00E+03,1.17E+03,8.27E+02,3.10E+02,3.1
63.13021,8.78E+02,8.93E+02,1.19E+02,5.61E+02,4.11E+02,5.12E+02,7.88E+02,1.03E+03,6.42E+02,2.7
63.13081,8.78E+02,6.66E+02,1.19E+02,9.11E+02,4.11E+02,5.12E+02,7.88E+02,1.62E+02,6.42E+02,2.7
63.13231,1.11E+03,7.40E+02,1.19E+02,9.75E+02,4.11E+02,8.11E+02,7.88E+02,1.14E+03,6.42E+02,2.7
63.13551,6.37E+02,4.81E+02,8.03E+02,9.84E+02,4.11E+02,2.62E+02,1.08E+03,4.71E+02,7.37E+02,2.7
63.13585,7.06E+02,2.98E+02,8.03E+02,8.59E+02,4.11E+02,2.62E+02,9.62E+02,4.71E+02,8.23E+02,2.7
63.13619,1.05E+03,4.08E+02,8.03E+02,6.35E+02,4.11E+02,2.62E+02,8.71E+02,4.71E+02,7.29E+02,2.7
63.14275,7.52E+02,7.54E+02,1.26E+02,6.70E+02,4.11E+02,8.40E+02,7.46E+02,6.52E+02,7.29E+02,4.6
63.14292,7.52E+02,7.54E+02,1.26E+02,6.70E+02,4.11E+02,3.31E+02,7.46E+02,6.52E+02,4.89E+02,4.6
63.14333,7.52E+02,7.54E+02,1.19E+03,6.70E+02,4.11E+02,8.04E+02,7.46E+02,6.52E+02,2.78E+02,4.6

63.15285,6.01E+02,7.97E+02,8.08E+02,7.22E+02,4.11E+02,3.80E+02,1.06E+02,3.52E+02,8.24E+02,8.7
63.15319,6.01E+02,1.17E+03,8.08E+02,5.30E+02,4.11E+02,3.80E+02,1.06E+02,3.52E+02,1.23E+02,6.9
63.15335,6.01E+02,1.17E+03,8.08E+02,8.24E+02,4.11E+02,3.80E+02,1.06E+02,3.52E+02,1.23E+02,5.1
63.1542,6.01E+02,7.09E+02,8.08E+02,7.85E+02,4.11E+02,3.80E+02,1.06E+02,8.50E+02,1.23E+02,6.39
63.16261,8.33E+02,9.11E+02,1.05E+03,1.09E+03,4.11E+02,3.51E+02,1.32E+03,3.15E+02,9.79E+02,1.3
63.16631,7.82E+02,9.11E+02,1.09E+03,9.38E+02,4.11E+02,2.91E+02,8.11E+02,9.32E+02,1.90E+02,1.3
63.17541,1.16E+03,1.06E+03,1.43E+03,9.54E+02,4.11E+02,7.86E+02,9.92E+02,1.28E+03,3.69E+02,3.4
63.17717,6.59E+02,6.66E+02,1.09E+03,7.81E+02,4.11E+02,4.33E+02,1.14E+03,1.02E+03,3.69E+02,3.4
63.17844,1.16E+03,9.20E+02,6.66E+02,4.75E+02,4.11E+02,4.33E+02,7.93E+02,5.03E+02,3.69E+02,3.4
63.17912,1.23E+03,5.13E+02,6.66E+02,4.75E+02,4.11E+02,8.26E+02,7.93E+02,9.59E+02,7.61E+02,3.4
63.18004,7.00E+02,5.41E+02,1.13E+03,8.76E+02,4.11E+02,9.31E+02,7.93E+02,7.63E+02,1.51E+02,8.0
63.21121,1.30E+03,5.88E+02,1.19E+03,4.06E+02,4.11E+02,6.10E+01,1.18E+03,8.69E+02,2.43E+02,3.9
63.2193,7.64E+02,7.94E+02,9.85E+02,9.13E+02,4.11E+02,6.19E+02,9.29E+02,2.32E+02,4.46E+02,4.90
63.2204,7.64E+02,1.11E+03,1.36E+03,9.82E+02,4.11E+02,6.19E+02,8.86E+02,1.10E+03,9.09E+02,4.90
63.24248,6.74E+02,2.05E+02,7.07E+02,5.30E+02,4.11E+02,7.10E+01,7.79E+02,4.75E+02,2.50E+02,4.9
63.2764,1.05E+03,5.89E+02,1.20E+03,9.00E+02,4.11E+02,2.32E+02,9.60E+02,1.10E+03,3.96E+02,3.32
63.2976,9.07E+02,5.50E+02,1.22E+03,5.86E+02,4.11E+02,2.32E+02,9.72E+02,2.71E+02,3.40E+01,3.32
63.31027,3.45E+02,4.43E+02,1.37E+03,4.69E+02,4.11E+02,7.80E+01,7.46E+02,4.25E+01,5.02E+02,3.3
63.992,6.99E+04,4.63E+04,7.35E+04,5.14E+04,2.42E+04,2.78E+04,6.59E+04,4.88E+04,4.39E+04,3.44E
66.03438,1.13E+03,1.46E+03,3.77E+02,4.25E+02,3.32E+03,2.15E+03,4.06E+02,1.08E+03,1.15E+03,9.8
68.99518,1.66E+02,9.56E+02,3.76E+02,9.90E+01,5.46E+02,1.18E+02,1.27E+02,1.54E+02,9.70E+01,1.9
69.03403,1.33E+03,1.24E+03,8.50E+00,4.73E+02,1.24E+03,1.02E+03,1.02E+03,1.02E+03,9.66E+02,1.1
70.02927,1.61E+02,5.88E+01,8.50E+00,9.90E+01,1.91E+03,9.21E+02,7.40E+02,1.82E+01,8.92E+01,2.2
71.0133,1.16E+05,1.48E+05,1.64E+05,9.87E+04,3.55E+05,1.92E+05,8.15E+04,2.07E+05,1.34E+05,2.20
72.00852,2.40E+03,2.33E+03,2.96E+03,1.45E+03,2.52E+04,1.98E+03,9.45E+02,1.66E+03,1.77E+03,3.3
72.01662,2.13E+03,2.09E+03,2.64E+03,1.55E+03,5.26E+03,2.55E+03,1.53E+03,2.75E+03,1.70E+03,3.1
72.99257,8.04E+03,1.34E+04,9.91E+03,8.40E+03,3.32E+04,2.91E+04,6.91E+03,7.24E+03,4.29E+03,2.1
73.02894,1.73E+04,7.21E+03,6.81E+03,1.08E+04,1.92E+04,1.53E+04,1.06E+04,1.02E+04,8.37E+03,1.7
74.02419,7.74E+03,1.38E+04,1.08E+04,9.91E+03,7.25E+04,2.34E+04,7.88E+03,3.43E+04,1.74E+04,2.5
75.00823,9.01E+04,1.31E+05,1.35E+05,8.32E+04,1.31E+05,1.17E+05,7.49E+04,9.30E+04,8.75E+04,1.2
76.01155,1.76E+03,2.17E+03,3.28E+03,1.10E+03,3.04E+03,1.29E+03,1.05E+03,1.51E+03,1.53E+03,1.5
76.9695,2.10E+02,4.45E+02,8.79E+02,4.52E+02,1.40E+02,1.67E+02,2.28E+02,3.02E+02,4.81E+02,2.12
77.01248,9.87E+02,1.24E+03,1.46E+03,7.26E+02,8.15E+02,9.12E+02,4.60E+01,1.04E+03,6.50E+00,1.1
78.95853,7.89E+04,4.37E+04,9.32E+04,1.14E+05,2.25E+05,6.11E+04,1.22E+05,7.40E+04,5.92E+04,1.3
79.95685,1.04E+04,1.27E+04,9.62E+03,6.13E+03,1.09E+04,3.37E+03,1.60E+04,1.49E+04,1.13E+04,1.3
81.03408,1.50E+01,2.45E+03,1.16E+01,6.97E+02,1.33E+03,1.31E+03,3.78E+02,1.37E+03,1.95E+01,1.0
82.94557,1.05E+03,1.31E+03,1.40E+03,1.04E+03,7.31E+02,7.95E+02,5.56E+02,8.76E+02,9.72E+02,1.0
83.01335,2.56E+03,2.23E+03,1.74E+03,1.62E+03,2.66E+03,2.29E+03,1.43E+03,2.88E+03,2.15E+03,2.5
83.04975,1.55E+03,1.64E+03,2.81E+02,1.84E+03,2.47E+03,2.16E+03,1.14E+03,2.52E+03,1.74E+03,2.6
84.94262,1.22E+03,3.65E+02,7.86E+02,6.92E+02,1.01E+03,5.44E+02,6.24E+02,6.44E+02,9.69E+02,9.9
85.02657,3.10E+02,2.66E+02,4.00E+02,2.74E+02,5.04E+02,2.37E+02,2.89E+02,2.12E+02,3.69E+02,3.6
85.02896,4.02E+04,3.49E+04,3.37E+04,3.08E+04,6.02E+04,5.17E+04,1.82E+04,7.55E+04,3.91E+04,4.6
86.02425,1.28E+02,1.94E+01,3.07E+02,4.95E+02,1.36E+03,1.27E+03,4.88E+01,2.03E+02,2.30E+01,5.0
86.03237,2.43E+03,1.91E+03,3.07E+02,1.41E+03,1.76E+03,1.38E+03,4.88E+01,2.00E+03,1.21E+03,1.1

86.51624,1.20E+02,1.87E+03,2.14E+03,1.73E+02,7.33E+01,1.77E+03,4.88E+01,1.99E+03,7.29E+02,7.2
87.0083,5.61E+04,7.12E+04,4.50E+04,4.80E+04,1.43E+05,9.71E+04,3.35E+04,1.18E+05,7.25E+04,1.07
87.04469,1.29E+03,1.83E+03,1.77E+03,1.86E+03,2.78E+03,2.66E+03,1.16E+03,2.75E+03,1.86E+03,3.5
88.01163,2.04E+03,1.19E+03,1.75E+03,1.18E+03,2.34E+03,1.70E+03,1.71E+03,1.68E+03,1.36E+03,1.7
88.03993,2.10E+04,1.81E+04,1.61E+04,9.87E+03,4.70E+04,1.82E+04,7.88E+03,1.07E+04,8.36E+03,2.9
88.97707,5.49E+01,9.40E+01,1.55E+02,2.38E+01,5.34E+02,1.35E+02,5.81E+01,2.06E+02,4.34E+02,6.3
88.98755,4.55E+03,3.09E+03,4.36E+02,1.20E+03,2.81E+03,2.67E+03,2.13E+03,8.48E+02,4.34E+02,1.1
89.00898,1.63E+02,2.54E+02,2.67E+02,2.13E+02,1.20E+02,2.16E+02,2.50E+02,2.91E+02,2.58E+02,2.0
89.02393,7.60E+04,1.08E+05,7.36E+04,8.82E+04,1.41E+05,1.66E+05,5.37E+04,1.03E+05,7.06E+04,1.0
89.03518,1.31E+04,8.00E+03,1.41E+04,2.20E+03,1.72E+05,8.10E+03,5.34E+03,9.05E+03,5.41E+03,1.7
90.02735,2.97E+03,1.91E+03,1.77E+03,1.31E+03,2.32E+03,2.28E+03,2.01E+03,2.35E+03,1.54E+03,1.8
91.02165,8.27E+02,8.12E+02,1.97E+02,9.80E+01,1.21E+03,6.64E+02,1.11E+02,5.34E+02,2.96E+02,1.2
91.03961,1.91E+03,2.06E+03,2.56E+03,9.09E+02,1.52E+03,1.06E+03,1.27E+03,1.43E+03,1.69E+03,1.4
91.98877,1.74E+03,3.28E+03,2.83E+03,8.43E+03,7.06E+03,3.26E+03,4.61E+03,2.49E+03,1.29E+03,2.8
92.92754,1.40E+03,7.75E+02,9.30E+01,2.03E+03,1.22E+02,5.62E+02,1.15E+03,1.26E+03,9.68E+02,1.0
93.03411,4.28E+02,6.86E+02,1.07E+03,1.73E+03,1.49E+03,7.80E+02,1.01E+03,1.09E+03,1.73E+03,1.1
94.92459,9.66E+02,1.35E+02,1.01E+02,1.26E+03,2.05E+02,8.40E+01,6.50E+02,1.10E+03,7.60E+01,8.8
94.98019,1.70E+03,1.14E+03,1.82E+03,9.98E+02,1.27E+03,1.13E+03,7.96E+02,1.09E+03,7.79E+02,1.5
95.00581,4.93E+03,2.60E+03,3.66E+03,2.59E+03,3.06E+03,2.12E+03,1.33E+03,3.38E+03,2.21E+03,2.9
95.01343,1.78E+03,9.21E+02,1.23E+03,9.39E+02,2.24E+03,1.76E+03,1.30E+03,1.64E+03,1.31E+03,1.4
95.02464,2.66E+03,2.23E+03,2.37E+03,2.86E+03,1.05E+04,1.69E+03,1.53E+03,1.20E+03,1.83E+03,6.7
95.0498,1.42E+03,1.48E+03,1.18E+02,1.48E+03,1.43E+03,1.49E+03,1.29E+03,1.94E+03,1.24E+03,1.66
96.04507,2.18E+03,3.12E+02,8.48E+02,1.33E+03,3.01E+03,1.57E+03,1.06E+03,1.99E+03,1.19E+03,2.1
96.95964,1.14E+06,1.37E+06,1.15E+06,7.49E+05,1.04E+06,4.16E+05,1.43E+06,1.29E+06,1.19E+06,1.2
96.96911,2.86E+05,1.43E+05,4.59E+05,4.61E+05,7.75E+05,2.58E+05,5.34E+05,2.92E+05,2.13E+05,5.3
97.02909,3.46E+03,2.23E+03,2.09E+03,1.83E+03,2.66E+03,2.45E+03,1.83E+03,2.71E+03,2.02E+03,2.8
97.06541,4.89E+03,6.75E+03,1.16E+03,7.69E+03,1.11E+04,8.59E+03,4.79E+03,1.13E+04,5.58E+03,1.3
97.95903,4.94E+03,6.00E+03,5.65E+03,3.10E+03,5.07E+03,1.98E+03,8.10E+03,5.50E+03,6.97E+03,5.3
97.96387,1.88E+03,1.49E+03,4.55E+01,1.00E+03,8.71E+02,2.44E+00,1.43E+03,1.61E+03,1.08E+03,1.8
98.9485,5.71E+02,1.30E+01,4.55E+01,5.55E+01,3.76E+02,1.87E+02,6.00E+01,4.78E+02,3.79E+02,2.65
98.95543,4.58E+04,5.49E+04,4.24E+04,2.55E+04,4.62E+04,1.23E+04,6.20E+04,5.04E+04,4.79E+04,4.9
98.96393,5.18E+03,6.54E+03,4.69E+03,3.07E+03,5.02E+03,2.68E+03,7.92E+03,6.60E+03,5.67E+03,5.6
98.97343,4.94E+03,1.65E+03,7.06E+03,3.53E+03,5.52E+03,2.82E+03,7.88E+03,3.35E+03,2.94E+03,5.7
99.0059,1.18E+02,1.27E+02,7.60E+01,1.91E+02,1.63E+02,1.03E+02,2.02E+02,1.08E+02,1.22E+02,1.23
99.00835,1.57E+04,1.27E+04,1.37E+04,1.19E+04,1.92E+04,1.36E+04,1.02E+04,2.15E+04,1.35E+04,2.2
99.04473,5.90E+03,6.31E+03,1.86E+03,6.97E+03,1.11E+04,8.60E+03,4.13E+03,1.11E+04,6.88E+03,1.3
101.02397,2.99E+04,2.82E+04,2.91E+04,2.81E+04,5.51E+04,5.03E+04,1.79E+04,4.60E+04,2.74E+04,3.
101.06039,1.61E+03,2.16E+03,1.42E+03,2.12E+03,3.40E+03,2.51E+03,1.80E+03,3.02E+03,2.23E+03,1.
102.01925,7.07E+01,4.90E+00,2.11E+03,5.10E+00,3.93E+03,1.05E+01,1.59E+02,1.41E+03,1.28E+03,1.
102.02737,2.15E+03,1.87E+03,1.73E+03,1.75E+03,1.95E+03,1.55E+03,7.67E+02,1.21E+03,1.40E+03,8.
102.05566,2.70E+04,1.39E+04,2.02E+04,1.06E+04,1.83E+04,1.78E+04,9.47E+03,1.52E+04,1.07E+04,1.
103.00326,1.32E+06,2.33E+06,1.86E+06,1.77E+06,1.88E+06,3.78E+06,1.14E+06,1.20E+06,2.11E+06,6.
103.03961,4.93E+03,6.59E+03,1.21E+03,2.85E+03,3.58E+03,6.45E+03,3.68E+03,1.16E+04,4.04E+03,4.
104.00663,4.36E+04,7.57E+04,5.92E+04,5.75E+04,6.29E+04,1.31E+05,3.20E+04,3.54E+04,6.75E+04,1.

104.01108,1.17E+05,8.36E+04,8.00E+04,1.04E+05,1.44E+05,1.07E+05,6.47E+04,7.22E+04,3.95E+04,8.
104.0349,1.58E+04,2.13E+04,1.71E+04,7.73E+03,1.23E+04,1.96E+04,5.60E+03,1.08E+04,2.47E+04,6.1
104.5127,1.94E+04,8.90E+03,8.73E+03,1.15E+04,1.94E+04,9.28E+03,8.03E+03,8.09E+03,4.37E+03,1.0
105.00746,9.26E+03,1.72E+04,8.92E+03,1.02E+04,1.13E+04,2.72E+04,8.24E+03,5.48E+03,1.31E+04,3.
105.01312,3.29E+03,4.49E+03,4.95E+03,4.93E+03,3.83E+03,2.95E+03,2.50E+03,3.74E+03,1.67E+03,2.
105.01885,9.53E+03,5.59E+04,1.63E+04,2.72E+04,7.58E+04,2.69E+04,9.34E+03,3.53E+04,3.44E+04,1.
106.02219,4.68E+01,1.76E+03,8.19E+01,2.60E+01,1.66E+03,3.68E+02,1.02E+01,2.09E+03,1.42E+03,1.
107.04984,3.57E+03,2.35E+03,1.90E+03,2.59E+03,6.90E+03,4.85E+03,1.25E+03,2.74E+03,2.39E+03,2.
107.94432,2.04E+03,2.96E+03,2.50E+03,1.45E+03,8.64E+01,1.25E+01,3.51E+03,1.84E+03,2.35E+03,1.
108.90152,1.04E+04,5.67E+03,3.40E+03,1.12E+04,2.71E+03,5.01E+03,7.81E+03,5.32E+03,4.97E+03,4.
108.99583,2.36E+03,3.18E+03,3.34E+03,2.70E+03,2.61E+03,2.69E+03,1.51E+03,1.91E+03,1.76E+03,3.
109.06547,1.35E+03,1.35E+03,7.38E+00,2.03E+03,2.84E+03,2.15E+03,8.67E+02,4.39E+03,2.28E+03,1.
110.02433,2.36E+03,2.56E+03,8.00E+00,2.21E+03,7.79E+03,5.81E+03,1.60E+02,2.31E+03,1.94E+03,2.
110.89858,6.53E+03,3.99E+03,2.51E+03,5.73E+03,3.06E+03,3.72E+03,3.33E+03,3.39E+03,3.32E+03,3.
110.9662,1.21E+03,2.32E+03,3.58E+03,1.53E+03,2.00E+03,1.49E+03,3.14E+03,2.58E+03,3.26E+03,1.5
110.97537,7.50E+02,3.49E+02,2.81E+02,3.14E+02,1.69E+02,4.08E+02,2.66E+02,1.11E+03,2.45E+02,1.
110.98487,2.90E+04,2.73E+04,9.56E+03,2.48E+04,1.33E+05,2.02E+04,1.69E+04,2.43E+04,3.37E+04,2.
111.00837,5.49E+04,4.34E+04,3.42E+04,2.90E+04,1.19E+05,4.54E+04,2.53E+04,4.80E+04,3.56E+04,7.
111.01894,1.89E+04,1.32E+04,1.29E+04,9.75E+03,7.57E+03,1.23E+04,7.03E+03,9.03E+03,4.82E+03,8.
111.04478,2.15E+03,2.14E+03,1.40E+01,1.90E+03,2.34E+03,2.35E+03,1.91E+03,2.71E+03,1.88E+03,2.
111.52059,2.49E+03,2.43E+03,1.40E+01,9.94E+02,1.52E+03,1.65E+03,1.15E+03,3.60E+01,4.66E+02,2.
112.01172,3.27E+03,2.40E+03,1.87E+03,1.59E+03,4.36E+03,1.64E+03,1.64E+03,1.74E+03,1.53E+03,2.
112.03998,1.64E+03,1.06E+02,1.92E+02,1.75E+03,1.30E+03,2.53E+03,1.27E+03,1.20E+01,4.30E+02,4.
112.8956,2.56E+03,1.06E+02,1.92E+02,1.57E+03,6.87E+02,1.68E+03,2.08E+03,1.24E+03,1.78E+02,1.3
112.98514,1.92E+03,1.70E+03,2.98E+03,2.38E+03,2.37E+03,9.52E+02,1.80E+03,1.61E+03,1.59E+03,3.
112.98766,6.04E+03,6.70E+03,6.18E+03,5.27E+03,5.53E+03,3.41E+03,5.54E+03,1.12E+04,7.11E+03,1.
113.024,1.60E+04,1.32E+04,1.69E+04,1.33E+04,2.94E+04,2.52E+04,9.13E+03,2.53E+04,1.20E+04,1.36
113.03525,1.11E+04,1.40E+04,2.39E+04,5.53E+03,1.55E+05,1.61E+04,8.18E+03,3.18E+03,1.39E+04,8.
113.06041,2.78E+04,2.63E+04,2.08E+04,1.55E+04,2.73E+04,2.38E+04,9.83E+03,2.81E+04,3.73E+04,3.
113.98795,7.84E+01,2.38E+03,2.30E+01,5.80E+00,2.92E+02,2.52E+03,1.07E+03,1.35E+03,2.69E+03,1.
114.01923,6.01E+03,6.70E+03,8.63E+03,3.16E+03,5.27E+04,8.43E+03,3.26E+03,2.15E+03,4.62E+03,2.
114.02736,1.72E+03,4.26E+02,8.66E+02,4.96E+02,4.13E+02,8.76E+02,9.00E+00,1.46E+03,9.63E+02,6.
114.0386,2.26E+03,1.77E+03,1.75E+03,1.07E+02,4.10E+03,2.00E+03,9.00E+00,7.13E+01,2.69E+03,2.3
114.05568,4.07E+03,2.94E+03,3.46E+03,2.65E+03,4.87E+03,4.16E+03,2.49E+03,2.70E+03,2.61E+03,4.
114.06371,2.33E+03,7.56E+02,1.76E+03,1.56E+03,1.96E+03,1.50E+03,1.20E+03,1.65E+03,1.95E+03,1.
114.93592,1.32E+03,1.24E+03,1.90E+03,1.66E+03,2.52E+03,2.65E+03,3.25E+02,1.26E+03,8.50E+00,8.
115.00331,6.10E+04,1.57E+05,1.61E+05,4.08E+04,3.12E+05,2.16E+05,4.80E+04,8.58E+04,7.46E+04,2.
115.01452,1.83E+03,1.20E+03,1.58E+03,1.22E+03,1.39E+04,1.71E+03,1.12E+03,8.94E+02,6.59E+02,1.
115.0397,3.50E+03,3.32E+03,2.29E+03,3.23E+03,8.63E+03,4.23E+03,2.58E+03,5.33E+03,3.64E+03,7.2
115.07602,2.63E+03,2.58E+03,1.64E+03,3.28E+03,4.99E+03,3.10E+03,2.14E+03,4.11E+03,2.72E+03,3.
116.00666,2.21E+03,5.89E+03,4.54E+03,1.71E+03,1.05E+04,6.68E+03,2.73E+03,2.41E+03,2.16E+03,9.
116.07137,2.84E+03,1.89E+03,1.46E+03,1.34E+03,2.68E+03,1.40E+03,1.24E+03,1.08E+03,1.23E+03,1.
116.90667,1.20E+05,1.70E+05,1.34E+05,1.07E+05,1.36E+05,1.47E+05,1.32E+05,1.18E+05,1.20E+05,1.
117.00402,1.19E+02,1.15E+02,1.85E+02,3.08E+02,1.26E+02,2.10E+01,7.10E+01,1.49E+02,1.42E+02,8.

117.01888,1.65E+05,6.26E+04,7.30E+04,1.19E+05,1.58E+05,1.08E+05,1.17E+05,8.19E+04,6.04E+04,1.
117.02651,6.25E+01,1.03E+03,3.21E+03,1.05E+03,8.12E+02,5.61E+02,4.01E+01,6.55E+02,8.06E+02,1.
117.03418,6.97E+02,9.79E+02,1.35E+03,4.24E+02,7.67E+02,8.17E+02,1.21E+03,1.21E+03,1.49E+03,9.
117.04044,1.06E+02,1.69E+02,3.43E+02,1.86E+02,2.20E+02,2.86E+02,3.00E+02,8.90E+01,1.95E+02,2.
117.05532,9.59E+02,1.68E+03,1.50E+01,1.40E+03,3.02E+03,1.73E+03,1.23E+03,1.45E+03,1.31E+03,2.
117.9101,1.81E+03,2.06E+03,1.79E+03,1.25E+03,2.39E+03,1.94E+03,1.64E+03,1.91E+03,2.87E+03,1.4
118.02236,3.91E+03,1.85E+03,1.91E+03,3.33E+03,4.07E+03,2.72E+03,3.08E+03,2.62E+03,1.89E+03,3.
118.0506,2.20E+04,1.03E+04,1.86E+04,3.24E+03,3.03E+04,8.89E+03,3.60E+03,1.56E+04,8.98E+03,1.3
118.9037,1.19E+05,1.59E+05,1.33E+05,1.03E+05,1.34E+05,1.42E+05,1.29E+05,1.11E+05,1.20E+05,1.2
118.94167,2.50E+03,2.36E+03,2.46E+03,2.34E+03,9.31E+02,1.04E+03,5.78E+03,3.75E+03,1.95E+03,3.
118.95125,1.62E+02,2.24E+02,1.59E+02,1.29E+02,2.20E+02,1.21E+02,1.49E+02,1.16E+02,2.28E+02,4.
119.03459,9.52E+03,1.04E+04,7.36E+03,9.61E+03,2.32E+04,2.16E+04,6.74E+03,1.53E+04,1.02E+04,1.
119.04983,5.97E+04,6.05E+04,7.26E+04,2.74E+04,5.66E+04,5.84E+04,3.99E+04,5.98E+04,6.14E+04,7.
119.90717,1.59E+03,2.58E+03,3.08E+03,1.42E+03,2.88E+02,2.10E+03,1.46E+03,1.68E+03,1.65E+03,1.
119.94656,7.86E+03,5.02E+03,2.67E+03,7.61E+03,1.02E+03,2.39E+03,7.04E+03,3.77E+03,2.52E+03,3.
120.0121,2.03E+03,1.98E+03,9.43E+01,1.18E+03,1.39E+02,2.48E+03,1.46E+03,1.71E+03,1.11E+02,5.0
120.05325,3.39E+03,3.06E+03,3.95E+03,1.95E+03,2.76E+03,2.73E+03,2.66E+03,3.44E+03,3.08E+03,3.
120.90078,3.47E+04,4.87E+04,3.61E+04,3.08E+04,3.87E+04,4.24E+04,3.75E+04,3.43E+04,3.19E+04,3.
120.99612,2.36E+03,2.49E+03,2.07E+03,1.43E+03,3.78E+03,2.42E+03,1.75E+03,2.27E+03,1.70E+03,1.
121.02907,3.66E+03,3.01E+03,3.51E+03,3.61E+03,5.66E+03,5.02E+03,3.72E+03,3.79E+03,3.98E+03,3.
121.06547,1.47E+03,2.78E+00,1.13E+00,1.37E+03,1.80E+03,1.08E+03,8.33E+02,5.38E+02,1.25E+03,1.
121.94357,2.67E+03,1.81E+03,2.68E+03,2.05E+03,5.89E+02,2.95E+02,2.46E+03,1.00E+00,1.41E+02,1.
122.00366,3.93E+03,5.76E+03,3.60E+03,1.49E+03,2.58E+03,2.46E+03,2.42E+03,3.14E+03,2.77E+03,2.
122.02438,5.84E+02,4.75E+02,5.54E+01,7.55E+01,1.90E+03,2.45E+03,5.96E+02,7.68E+01,1.20E+03,4.
122.89787,5.82E+03,4.82E+03,3.91E+03,3.80E+03,3.68E+03,3.99E+03,3.45E+03,3.34E+03,4.19E+03,3.
123.01154,1.10E+03,1.27E+03,1.20E+03,3.94E+02,9.11E+02,1.11E+03,4.16E+02,6.32E+02,3.83E+02,1.
123.02455,2.11E+03,1.64E+03,2.67E+03,1.19E+03,2.87E+03,1.55E+03,2.47E+03,1.24E+03,1.95E+03,2.
123.0448,9.58E+02,1.91E+03,2.32E+02,1.28E+03,1.92E+03,1.49E+03,4.69E+02,1.51E+03,1.17E+03,1.1
123.0812,1.21E+03,1.49E+03,2.32E+02,8.38E+02,1.00E+03,2.27E+01,1.41E+03,4.97E+02,1.95E+03,1.0
123.90164,1.97E+03,2.56E+03,2.32E+02,1.94E+03,2.33E+03,1.74E+03,2.82E+03,1.84E+03,2.97E+03,5.
123.97883,1.51E+02,7.88E+02,7.08E+02,1.61E+03,4.99E+02,4.30E+01,3.19E+02,1.45E+03,1.31E+03,1.
124.98532,4.53E+02,1.27E+03,1.17E+03,2.02E+03,3.39E+02,2.41E+03,1.85E+03,1.80E+03,7.92E+02,8.
125.01933,9.00E+01,7.20E+01,8.80E+01,5.60E+01,8.50E+01,1.41E+02,1.35E+02,1.27E+02,1.73E+02,1.
125.02405,2.28E+03,2.84E+03,2.13E+03,1.69E+03,2.56E+03,3.42E+03,2.25E+03,3.05E+03,1.83E+03,2.
125.06042,2.60E+03,1.22E+03,2.63E+01,1.74E+03,2.77E+03,2.12E+03,1.07E+03,2.08E+03,1.61E+03,2.
125.09679,1.82E+03,1.43E+03,5.49E+02,9.15E+02,2.28E+03,1.52E+03,1.87E+03,1.76E+03,1.53E+03,1.
125.89985,1.42E+03,3.66E+01,1.58E+03,1.53E+03,1.43E+03,1.30E+01,3.30E+01,1.16E+03,1.40E+01,1.
125.97148,5.94E+02,2.05E+03,1.73E+03,7.30E+01,2.10E+02,1.30E+01,3.30E+01,3.56E+02,1.14E+03,1.
126.01934,2.30E+02,8.08E+02,1.96E+03,1.03E+02,1.85E+03,1.57E+03,3.30E+01,3.52E+02,3.27E+01,8.
126.93553,2.92E+03,2.93E+03,3.40E+03,3.36E+03,4.20E+03,2.96E+03,2.58E+03,3.69E+03,2.95E+03,3.
126.97047,2.96E+03,1.97E+03,2.46E+03,1.36E+03,2.28E+03,1.35E+03,9.59E+02,2.00E+03,2.05E+03,2.
126.98844,8.34E+02,1.33E+03,1.07E+03,2.13E+01,8.50E+01,2.13E+03,5.90E+02,1.69E+03,3.49E+02,8.
127.03976,6.15E+03,6.67E+03,1.15E+02,8.00E+03,1.05E+04,8.53E+03,4.90E+03,1.02E+04,8.01E+03,1.
127.0761,3.43E+03,2.19E+03,1.90E+03,4.33E+03,6.21E+03,4.28E+03,3.58E+03,1.04E+04,3.12E+03,7.6

127.96886,2.42E+03,1.30E+00,1.48E+01,1.67E+03,7.57E+02,7.59E+02,1.87E+03,1.59E+03,1.35E+03,1.
128.0349,5.25E+04,3.06E+04,3.95E+04,2.30E+04,3.18E+04,3.47E+04,1.65E+04,2.88E+04,2.24E+04,3.1
128.93269,2.59E+03,2.36E+03,3.10E+03,2.15E+03,2.86E+03,1.82E+03,1.77E+03,3.07E+03,1.96E+03,2.
128.96835,2.94E+03,9.82E+02,2.30E+03,7.08E+02,2.40E+03,1.45E+03,1.88E+00,7.01E+02,1.20E+03,9.
128.99338,5.33E+00,2.79E+03,2.27E+03,2.10E+03,2.65E+02,3.33E+03,9.24E+02,4.07E+02,2.06E+03,1.
129.01898,2.98E+04,3.00E+04,2.57E+04,2.14E+04,4.30E+04,3.77E+04,1.53E+04,5.61E+04,3.07E+04,4.
129.03836,3.26E+03,1.43E+03,1.55E+03,1.45E+03,1.98E+03,1.51E+03,1.55E+03,1.66E+03,1.67E+03,1.
129.05536,7.60E+03,8.18E+03,3.84E+03,9.50E+03,4.08E+04,1.33E+04,6.70E+03,1.45E+04,9.93E+03,2.
129.09175,1.65E+03,1.79E+03,1.12E+03,1.97E+03,2.45E+03,2.35E+03,1.14E+03,3.04E+03,1.80E+03,2.
130.02228,1.84E+03,1.94E+03,1.89E+03,1.16E+03,2.64E+03,1.88E+03,8.00E+02,2.18E+03,1.45E+03,1.
130.05057,1.46E+03,1.29E+03,2.64E+03,1.07E+03,4.89E+03,2.82E+03,9.96E+02,3.32E+03,1.85E+03,2.
130.08703,8.53E+03,4.69E+03,1.34E+04,4.72E+03,2.55E+04,5.86E+03,1.94E+03,8.15E+03,6.41E+03,1.
130.92966,1.04E+02,2.87E+01,3.38E+01,6.81E+02,1.05E+03,1.81E+02,6.93E+02,7.03E+02,1.12E+03,2.
130.99169,1.50E+04,9.90E+03,1.49E+04,3.73E+03,5.00E+00,1.42E+03,2.40E+04,4.89E+03,6.79E+03,1.
131.0196,1.92E+02,1.62E+02,7.60E+01,5.16E+01,1.65E+02,9.10E+01,6.60E+01,2.41E+02,8.70E+01,1.2
131.03467,7.70E+03,2.35E+04,6.04E+03,9.71E+03,1.86E+04,2.37E+04,9.75E+03,1.10E+04,1.46E+04,1.
131.0459,8.06E+04,1.15E+05,1.79E+05,4.95E+04,8.79E+05,1.30E+05,6.24E+04,2.71E+04,9.71E+04,5.2
131.09043,2.08E+02,1.24E+03,2.07E+03,2.53E+02,5.74E+02,8.10E+01,1.74E+02,2.22E+03,1.86E+03,3.
132.0298,2.30E+05,1.82E+05,2.29E+05,1.55E+05,3.02E+05,1.40E+05,8.77E+04,1.18E+05,1.14E+05,1.9
132.0411,4.46E+04,2.72E+04,5.17E+04,8.13E+03,3.69E+05,2.64E+04,1.61E+04,2.77E+04,1.79E+04,5.1
132.04913,3.62E+03,3.47E+03,8.09E+03,2.22E+03,3.40E+04,3.91E+03,3.17E+03,2.37E+03,3.66E+03,1.
132.06627,3.96E+01,1.23E+03,1.85E+03,1.35E+03,1.42E+03,1.86E+03,3.75E+01,8.99E+02,2.36E+01,1.
132.946,2.03E+03,1.25E+03,4.34E+03,1.83E+03,2.05E+03,1.25E+03,3.37E+03,1.30E+03,2.19E+03,1.16
133.01384,3.05E+05,1.07E+06,1.06E+06,2.57E+05,1.89E+06,1.41E+06,2.69E+05,5.38E+05,4.55E+05,1.
133.03326,9.15E+03,3.38E+02,2.52E+03,4.53E+03,4.04E+03,1.75E+02,3.30E+03,2.90E+03,6.96E+02,7.
133.05026,4.31E+03,3.68E+03,4.23E+03,3.44E+03,1.97E+04,5.01E+03,3.87E+03,6.78E+03,2.63E+03,8.
133.96005,2.28E+03,3.64E+03,2.00E+03,3.73E+03,4.29E+03,4.86E+03,2.07E+03,1.77E+03,4.85E+03,1.
134.01722,1.19E+04,4.61E+04,4.17E+04,9.33E+03,8.01E+04,5.99E+04,8.04E+03,2.01E+04,1.89E+04,8.
134.03411,2.22E+03,3.13E+03,2.15E+03,1.43E+03,3.13E+03,7.27E+01,8.82E+02,1.97E+03,1.85E+03,2.
134.03696,4.02E+03,2.56E+03,4.22E+03,1.71E+03,2.30E+03,3.81E+03,3.45E+03,2.90E+03,5.42E+03,1.
134.04685,2.64E+03,3.42E+03,3.43E+03,1.83E+03,4.51E+03,3.85E+03,2.01E+03,1.98E+03,2.53E+03,3.
134.87486,1.37E+02,1.48E+02,1.64E+02,5.91E+02,3.85E+02,1.97E+02,8.60E+01,1.83E+02,4.80E+02,2.
134.91557,2.72E+04,3.42E+04,2.55E+04,1.39E+04,7.24E+03,5.46E+03,5.69E+04,1.30E+04,1.32E+04,1.
134.95846,1.55E+03,3.56E+03,1.83E+03,2.71E+03,3.00E+01,3.58E+03,2.97E+03,2.70E+01,2.56E+03,4.
134.96786,2.30E+04,1.41E+04,1.05E+04,7.87E+03,1.45E+04,9.57E+03,7.80E+03,1.17E+04,9.29E+03,1.
135.01812,3.50E+03,7.78E+03,8.88E+03,2.17E+03,1.59E+04,1.13E+04,2.50E+03,4.55E+03,3.14E+03,1.
135.02948,1.97E+04,4.23E+04,2.62E+04,1.18E+04,6.98E+04,6.01E+04,7.66E+03,1.29E+04,3.04E+04,3.
135.04474,1.40E+03,8.28E+02,1.86E+03,1.22E+03,2.08E+03,1.69E+03,1.43E+03,1.29E+03,1.61E+03,1.
135.46961,3.24E+03,2.36E+03,6.05E+02,1.18E+03,1.40E+03,1.69E+03,5.40E+02,1.13E+03,1.60E+03,1.
135.92047,5.00E+04,3.13E+04,1.99E+04,2.89E+04,3.74E+03,9.10E+03,4.95E+04,1.25E+04,1.34E+04,1.
135.95793,2.96E+03,2.20E+03,1.19E+02,2.19E+03,3.58E+01,1.93E+03,3.99E+02,7.12E+01,1.63E+03,6.
135.96638,1.32E+04,7.58E+03,7.51E+03,6.70E+03,7.97E+03,4.95E+03,5.37E+03,5.25E+03,6.08E+03,7.
136.00109,1.87E+03,1.95E+03,3.26E+03,6.92E+02,3.63E+02,1.90E+03,7.67E+00,3.22E+03,1.73E+03,1.
136.03281,4.67E+02,2.07E+03,1.47E+03,8.63E+02,2.10E+03,1.61E+03,7.67E+00,1.21E+03,1.70E+03,2.

136.03998,1.32E+03,1.53E+03,1.74E+03,3.08E+02,1.32E+03,3.10E+03,7.67E+00,1.44E+03,1.32E+03,1.
136.46693,3.93E+03,2.42E+03,1.70E+03,1.04E+03,1.65E+03,9.92E+02,1.05E+03,2.18E+03,2.26E+03,2.
136.8718,2.56E+02,1.94E+02,1.65E+02,1.47E+02,3.76E+02,3.36E+02,2.42E+02,1.79E+02,2.00E+02,1.6
136.91141,1.24E+03,1.69E+03,2.40E+03,1.65E+03,1.20E+01,1.70E+01,2.39E+03,8.80E+02,8.00E+00,1.
136.91371,1.60E+03,2.71E+03,4.18E+03,2.13E+03,1.20E+01,1.70E+01,2.96E+03,4.66E+02,1.51E+03,1.
136.96579,7.24E+03,6.28E+03,4.33E+03,2.52E+03,6.19E+03,5.30E+03,4.16E+03,3.80E+03,5.17E+03,5.
137.0241,1.08E+03,1.31E+03,2.02E+03,2.61E+03,6.31E+03,1.99E+03,1.07E+03,1.53E+03,1.29E+03,1.1
137.04029,1.61E+03,2.06E+03,5.25E+02,1.84E+01,1.57E+03,1.95E+03,2.15E+03,9.40E+02,5.17E+00,1.
137.06046,1.30E+03,9.37E+02,2.51E+02,1.46E+03,1.42E+03,2.14E+03,1.78E+03,1.48E+03,1.71E+03,2.
137.09694,4.75E+00,5.36E+02,2.51E+02,9.51E+02,1.26E+03,9.27E+01,1.79E+02,1.03E+03,1.15E+03,1.
137.91762,1.65E+04,7.34E+03,7.45E+03,8.89E+03,1.30E+03,4.55E+03,1.21E+04,4.46E+03,5.31E+03,4.
138.05569,2.06E+03,1.44E+03,2.53E+03,1.65E+03,2.02E+03,1.72E+03,1.34E+03,2.26E+03,1.28E+03,1.
138.86877,1.15E+02,7.96E+01,3.17E+02,6.90E+01,9.30E+01,9.90E+01,1.01E+03,1.24E+02,1.30E+01,1.
138.93747,2.61E+03,7.96E+01,3.17E+02,1.26E+03,6.00E+00,2.40E+03,1.42E+03,1.59E+03,1.64E+03,7.
139.02147,2.38E+04,3.07E+04,5.03E+04,9.04E+03,1.97E+04,1.53E+04,1.58E+04,2.45E+04,1.89E+04,3.
139.03965,1.06E+03,3.27E+01,7.84E+01,1.82E+02,1.66E+03,1.66E+03,6.86E+02,1.13E+03,5.52E+02,9.
139.0761,1.83E+03,1.39E+03,7.84E+01,2.06E+03,2.55E+03,1.67E+03,1.47E+03,2.77E+03,1.72E+03,3.1
139.52326,5.28E+03,4.77E+03,9.21E+03,1.45E+03,4.07E+03,2.52E+03,2.92E+03,4.09E+03,3.84E+03,5.
140.02374,1.93E+03,1.37E+03,3.27E+01,1.06E+02,7.38E+02,5.19E+02,2.06E+03,3.07E+02,7.96E+02,5.
140.95914,4.33E+03,9.35E+03,6.42E+03,5.43E+03,2.69E+03,9.45E+03,5.45E+03,2.84E+03,4.69E+03,1.
140.97619,3.96E+03,6.98E+01,1.20E+04,4.63E+03,1.04E+04,1.05E+03,5.37E+03,2.47E+03,6.75E+03,3.
141.01891,1.30E+03,1.75E+03,1.27E+02,1.26E+03,2.18E+03,1.18E+03,1.42E+03,2.44E+03,1.09E+03,2.
141.05534,1.66E+03,1.32E+03,1.27E+02,1.64E+03,1.83E+03,1.54E+03,1.33E+03,1.75E+03,1.52E+03,1.
141.09168,1.59E+03,8.74E+02,1.27E+02,1.22E+03,2.02E+03,1.24E+03,1.37E+03,1.25E+03,1.22E+03,1.
141.95439,1.57E+03,2.12E+03,2.09E+03,1.74E+03,7.88E+01,1.01E+02,1.80E+03,1.08E+02,1.34E+03,1.
141.97584,3.84E+03,3.39E+03,3.27E+03,2.71E+03,2.45E+03,2.52E+03,2.58E+03,2.50E+03,2.36E+03,3.
142.01423,1.57E+03,2.56E+03,2.80E+03,1.67E+03,1.67E+04,1.99E+04,3.88E+01,1.80E+01,1.39E+03,7.
142.05074,2.29E+03,2.22E+03,2.62E+03,2.24E+03,3.61E+03,6.08E+03,1.83E+03,1.57E+03,1.54E+03,3.
142.97423,3.65E+03,2.39E+03,3.62E+01,1.74E+03,1.71E+03,2.20E+03,2.41E+03,3.31E+03,1.10E+03,1.
142.98339,1.44E+03,2.53E+03,8.45E+02,1.09E+03,1.51E+02,1.99E+03,1.96E+03,5.90E+01,1.11E+03,9.
142.99829,5.98E+03,5.65E+03,4.14E+03,4.68E+03,4.22E+03,2.66E+03,6.44E+03,1.04E+04,6.75E+03,9.
143.0347,9.29E+03,1.03E+04,7.98E+03,8.35E+03,1.61E+04,1.72E+04,6.47E+03,1.41E+04,7.55E+03,9.9
143.0711,3.48E+03,3.98E+03,4.44E+02,4.97E+03,6.77E+03,5.28E+03,3.29E+03,5.63E+03,3.24E+03,8.0
143.1074,2.42E+03,2.89E+03,2.81E+03,3.74E+03,2.68E+03,3.91E+03,2.02E+03,3.58E+03,3.20E+03,3.4
143.95096,3.09E+03,3.78E+03,4.24E+03,3.23E+03,4.83E+03,3.95E+03,1.89E+03,4.82E+03,3.69E+03,6.
143.97354,1.97E+03,2.31E+03,6.10E+01,1.47E+03,1.69E+03,8.07E+02,1.28E+02,3.85E+02,7.04E+01,1.
144.02984,1.91E+03,1.99E+03,9.85E+02,1.05E+03,2.74E+03,2.37E+03,1.38E+03,9.40E+02,1.80E+03,1.
144.03796,9.03E+02,9.83E+01,1.38E+03,5.90E+01,2.16E+03,1.36E+03,2.12E+02,4.47E+02,1.77E+02,7.
144.06626,1.43E+03,9.83E+01,1.50E+03,5.90E+01,2.61E+03,7.86E+02,2.12E+02,1.57E+03,1.17E+03,1.
144.94945,3.23E+03,2.92E+03,3.06E+03,3.09E+03,4.00E+03,3.00E+03,1.17E+03,3.88E+03,2.98E+03,5.
145.01393,9.79E+03,1.31E+04,1.33E+04,1.08E+04,3.41E+04,2.16E+04,7.33E+03,1.54E+04,1.32E+04,1.
145.02916,2.69E+03,2.87E+03,2.99E+03,1.98E+03,1.98E+03,2.30E+03,1.65E+03,2.02E+03,2.11E+03,2.
145.05028,4.66E+03,5.62E+03,2.16E+03,6.86E+03,1.25E+04,7.05E+03,3.25E+03,9.29E+03,4.71E+03,1.
145.06159,3.26E+03,6.42E+03,6.78E+03,1.47E+03,1.11E+04,6.19E+03,2.02E+03,4.38E+03,3.79E+03,1.

145.08675,2.29E+03,1.64E+01,4.77E+01,9.77E+02,1.33E+03,7.26E+02,1.23E+03,1.86E+03,6.26E+01,1.
145.94869,1.80E+03,1.53E+03,2.12E+03,6.36E+02,1.89E+03,3.07E+03,1.14E+03,1.93E+03,1.78E+03,2.
145.97378,6.43E+03,1.89E+03,7.16E+03,3.07E+03,3.25E+03,2.11E+03,4.84E+03,4.93E+03,5.09E+03,3.
146.04558,6.75E+05,4.38E+05,6.17E+05,3.61E+05,7.38E+05,4.94E+05,2.59E+05,5.63E+05,4.13E+05,4.
146.96557,5.29E+04,1.92E+04,4.07E+04,2.86E+04,1.35E+03,4.15E+03,6.22E+04,8.78E+03,1.28E+04,7.
147.02954,5.42E+05,5.47E+05,5.95E+05,3.20E+05,2.76E+05,6.53E+05,2.22E+05,1.40E+06,7.52E+05,4.
147.04879,2.63E+04,1.77E+04,2.32E+04,1.30E+04,2.90E+04,1.98E+04,9.37E+03,2.33E+04,1.56E+04,1.
147.06583,1.45E+03,1.46E+03,1.15E+03,3.15E+03,8.44E+03,2.97E+03,2.13E+03,1.03E+03,7.00E-01,3.[illegible]
148.03291,2.29E+04,2.38E+04,2.54E+04,1.24E+04,9.51E+03,3.05E+04,8.82E+03,7.21E+04,3.30E+04,1.
148.04979,3.77E+03,2.03E+03,3.50E+03,1.62E+03,3.30E+03,2.55E+03,1.62E+03,2.62E+03,2.16E+03,2.
148.96532,2.46E+03,2.96E+03,4.48E+03,4.96E+01,2.71E+03,4.19E+03,1.29E+03,2.02E+03,1.80E+03,2.
149.03373,4.53E+03,1.07E+02,5.88E+03,1.78E+03,9.14E+02,5.34E+02,4.50E+00,9.80E+03,1.92E+03,2.
149.0452,1.18E+05,8.15E+05,1.55E+05,1.82E+05,1.55E+05,6.49E+05,3.51E+05,7.30E+04,4.65E+05,8.3
149.06033,4.17E+03,6.35E+02,2.89E+03,4.45E+03,7.38E+03,5.39E+03,6.98E+02,3.20E+03,4.22E+03,3.
149.96378,1.99E+03,2.43E+03,2.06E+03,1.35E+03,6.31E+03,2.14E+03,3.39E+02,2.70E+00,1.51E+03,2.
150.04852,4.77E+03,3.02E+04,5.03E+03,5.33E+03,5.34E+03,2.43E+04,1.28E+04,2.69E+03,1.85E+04,3.
150.05566,1.72E+02,1.59E+03,1.37E+03,1.79E+02,2.39E+03,1.04E+03,3.03E+01,4.06E+02,2.49E+01,1.
150.86968,2.09E+03,1.91E+03,1.37E+03,1.99E+03,2.59E+02,4.72E+03,1.34E+02,1.06E+03,1.93E+03,1.
150.96322,6.78E+01,1.22E+03,6.88E+02,6.56E+02,2.61E+03,1.36E+03,1.92E+02,1.03E+02,8.04E+02,1.
151.03973,7.55E+02,1.25E+02,1.58E+02,4.37E+02,9.94E+02,9.70E+02,3.96E+02,9.86E+02,2.05E+02,1.
151.04943,1.30E+01,4.63E+03,2.13E+03,8.38E+02,9.20E+02,3.37E+03,2.23E+03,8.10E+01,2.44E+03,8.
151.06087,1.30E+01,1.34E+03,3.26E+03,7.67E+01,7.00E+01,2.56E+03,1.29E+03,1.04E+03,1.55E+03,1.
151.07613,7.43E+02,1.02E+03,8.15E+01,1.22E+03,1.37E+03,1.91E+03,1.68E+03,1.14E+03,1.33E+03,1.
152.86668,1.17E+03,2.67E+03,1.64E+03,2.17E+03,1.21E+03,4.06E+03,1.86E+03,1.40E+03,2.04E+03,1.
152.99548,1.18E+03,3.40E+01,1.68E+03,1.54E+03,2.95E+03,2.32E+03,1.60E+03,1.67E+03,4.77E+02,1.
153.05535,1.42E+03,1.60E+03,1.60E+03,1.72E+03,2.01E+03,1.14E+03,1.30E+03,2.40E+03,1.54E+03,2.
153.09169,1.82E+03,2.28E+03,2.86E+02,1.64E+03,2.12E+03,1.40E+03,1.39E+03,1.95E+03,1.45E+03,2.
154.02685,1.47E+03,1.58E+03,2.03E+03,1.00E+03,1.27E+03,1.17E+03,2.47E+03,1.57E+03,1.35E+03,8.
154.86385,4.70E+01,1.24E+03,5.20E+01,7.27E+02,2.96E+02,1.57E+03,1.86E+03,6.80E+01,1.92E+03,6.
154.86548,4.70E+01,1.73E+03,5.20E+01,6.50E+00,2.96E+02,1.23E+03,5.89E+02,4.00E+01,2.66E+02,9.
154.90585,2.07E+03,1.96E+03,5.20E+01,1.43E+03,1.16E+03,3.10E+02,2.28E+03,1.32E+03,1.81E+03,1.
154.97897,3.71E+03,3.24E+03,2.46E+03,3.34E+03,2.21E+03,2.04E+03,4.14E+03,2.98E+03,4.70E+03,4.
154.99582,1.91E+02,1.71E+03,1.34E+02,1.88E+02,2.53E+03,3.10E+03,1.39E+02,5.47E+02,1.16E+02,2.
155.03462,1.14E+03,1.03E+03,1.80E+01,1.36E+03,1.16E+03,1.69E+03,1.26E+03,9.50E+02,1.49E+03,1.
155.07096,2.07E+03,3.02E+03,1.80E+01,2.19E+03,2.56E+03,2.68E+03,1.89E+03,3.53E+03,2.11E+03,3.
155.10742,1.22E+03,3.32E+03,1.80E+01,1.40E+03,1.27E+03,1.16E+03,1.03E+03,1.56E+03,1.06E+03,1.
155.91068,4.50E+03,1.92E+03,2.13E+03,4.23E+03,1.56E+02,4.50E+00,4.44E+03,2.43E+03,2.51E+03,2.
155.97312,2.18E+03,1.84E+03,4.77E+03,2.03E+03,2.82E+03,1.97E+03,2.31E+03,3.04E+03,3.21E+03,1.
155.97785,1.84E+02,1.47E+03,2.66E+03,3.13E+01,1.50E+03,5.70E+01,1.14E+02,2.94E+01,1.34E+01,1.
156.06621,1.74E+03,2.97E+02,8.68E+02,2.38E+03,3.81E+03,1.98E+03,2.33E+03,1.45E+03,1.69E+03,2.
156.90361,2.68E+03,1.57E+03,2.20E+03,2.62E+03,3.92E+03,4.39E+03,1.49E+03,1.25E+03,2.67E+03,1.
156.9717,1.83E+03,2.05E+03,2.11E+03,1.46E+03,1.45E+03,2.14E+03,8.50E+02,1.70E+03,2.18E+03,2.3
157.03643,1.62E+03,4.67E+01,5.95E+01,2.27E+02,6.12E+03,1.57E+03,6.80E+01,1.44E+03,1.40E+01,1.
157.05029,1.04E+05,1.01E+05,9.65E+04,4.42E+04,7.71E+04,7.31E+04,2.61E+04,9.10E+04,1.28E+05,1.

157.08668,9.37E+02,1.55E+03,8.91E+01,1.06E+03,1.94E+03,9.21E+02,1.25E+03,1.77E+03,1.33E+03,1.
157.12317,2.00E+04,1.46E+04,1.32E+04,2.65E+04,2.95E+04,1.90E+04,1.01E+04,2.82E+04,1.49E+04,3.
157.90782,3.13E+03,2.69E+03,2.64E+03,1.42E+03,3.82E+00,1.67E+01,2.26E+03,2.12E+03,2.48E+03,1.
157.99608,2.70E+03,2.06E+03,2.00E+02,1.63E+03,4.54E+02,1.48E+03,1.29E+03,1.05E+02,1.65E+03,4.
158.0204,3.10E+01,2.15E+02,1.05E+03,2.35E+02,4.15E+03,3.86E+02,1.74E+02,1.05E+02,3.40E+02,4.4
158.0456,3.10E+01,1.00E+03,2.69E+02,1.08E+03,1.70E+03,2.32E+03,5.02E+02,1.05E+02,3.40E+02,4.4
158.05367,4.76E+03,4.22E+03,4.51E+03,2.50E+03,5.02E+03,3.70E+03,2.49E+03,5.19E+03,5.54E+03,6.
158.12639,2.53E+03,1.49E+03,1.71E+03,1.89E+03,2.34E+03,1.68E+03,1.73E+03,1.99E+03,1.62E+03,2.
158.90063,1.30E+03,1.72E+03,1.53E+03,2.43E+03,1.88E+03,3.66E+03,1.99E+03,1.76E+03,2.15E+03,1.
159.02947,3.76E+03,2.05E+03,4.56E+03,2.30E+03,4.50E+03,3.99E+03,1.53E+03,3.25E+03,2.16E+03,2.
159.0428,2.01E+03,2.37E+02,2.22E+03,8.90E+02,3.72E+02,1.42E+03,1.29E+01,8.70E+00,6.55E+01,4.4
159.06583,4.39E+03,4.33E+03,1.59E+03,6.51E+03,1.09E+04,7.05E+03,3.31E+03,7.63E+03,4.97E+03,8.
159.07715,1.20E+00,6.52E+01,2.28E+02,7.92E+02,1.66E+03,1.49E+03,1.75E+01,3.95E+01,4.82E+00,1.
159.54215,2.70E+03,9.00E+00,4.58E+03,2.50E+02,1.30E+03,3.89E+02,1.10E+03,4.82E+02,7.37E+02,1.
159.96503,1.88E+03,2.64E+03,2.47E+03,1.86E+03,1.49E+02,2.36E+03,2.06E+03,1.62E+03,1.70E+03,1.
160.06132,1.69E+04,1.55E+04,1.69E+04,2.89E+04,5.94E+04,2.73E+04,1.56E+04,1.84E+04,8.44E+03,4.
160.89659,5.79E+03,8.71E+03,8.61E+03,4.53E+03,6.38E+03,7.25E+03,6.89E+03,5.53E+03,6.81E+03,5.
160.89967,1.20E+01,1.16E+03,5.04E+01,1.27E+03,1.45E+03,1.91E+03,9.01E+01,9.53E+01,1.19E+03,3.
160.94936,1.99E+03,3.90E+03,2.89E+03,3.43E+03,3.71E+01,6.79E+03,1.59E+03,2.78E+03,6.67E+03,1.
161.04521,8.39E+04,5.16E+04,1.28E+05,4.46E+04,1.02E+05,9.01E+04,2.92E+04,9.99E+04,4.36E+04,5.
161.0646,3.48E+03,1.23E+03,1.00E+03,1.41E+03,2.17E+03,1.75E+03,1.56E+03,7.00E+00,2.15E+03,2.0
161.08157,2.89E+03,2.41E+03,2.66E+03,2.25E+03,1.15E+04,3.58E+03,1.64E+03,3.94E+03,2.20E+03,4.
162.04848,4.20E+03,2.20E+03,5.25E+03,1.94E+03,3.82E+03,3.20E+03,2.40E+03,3.72E+03,1.90E+03,2.
162.89368,6.26E+03,7.36E+03,6.85E+03,5.05E+03,5.91E+03,5.18E+03,6.43E+03,5.89E+03,5.60E+03,6.
162.93939,1.72E+05,6.52E+04,1.07E+05,4.37E+04,2.63E+03,8.60E+03,1.97E+05,1.13E+04,1.87E+04,9.
162.94654,1.04E+02,1.27E+03,1.01E+02,1.58E+03,1.33E+00,2.62E+03,1.56E+01,7.50E+01,1.54E+03,1.
163.02471,4.40E+03,2.01E+03,1.01E+02,2.95E+03,5.46E+03,1.95E+03,1.25E+03,1.84E+03,4.32E+02,8.
163.03976,6.38E+05,6.65E+05,7.96E+05,3.17E+05,6.51E+05,6.22E+05,4.31E+05,6.25E+05,6.45E+05,7.
163.06097,4.18E+03,5.09E+03,3.59E+03,5.51E+03,1.82E+04,9.44E+03,1.14E+03,2.07E+03,1.86E+03,5.
163.97065,8.40E+01,2.87E+03,3.18E+03,6.18E+02,3.80E+03,2.90E+03,1.87E+02,1.77E+02,3.24E+01,4.
164.04311,4.74E+04,5.39E+04,6.21E+04,1.75E+04,4.79E+04,4.85E+04,3.06E+04,4.99E+04,4.41E+04,6.
164.07134,2.70E+03,4.06E+03,3.74E+03,2.63E+03,6.91E+03,2.59E+03,1.96E+03,2.79E+03,4.12E+03,4.
164.89076,2.62E+03,2.93E+03,3.67E+03,2.89E+03,3.47E+03,2.92E+03,3.28E+03,3.32E+03,2.82E+03,3.
164.93769,9.30E+03,3.35E+03,5.31E+03,2.67E+03,2.27E+02,3.12E+02,1.19E+04,1.81E+03,2.16E+03,1.
164.95328,1.93E+03,1.61E+03,1.62E+03,9.03E+02,2.17E+03,2.00E+03,1.12E+03,1.58E+03,1.61E+03,2.
164.96362,3.64E+03,4.00E+03,2.22E+03,1.91E+03,8.00E+00,1.48E+03,8.16E+03,8.85E+01,1.70E+03,2.
165.0401,2.45E+04,4.33E+04,3.55E+04,1.70E+04,1.09E+05,6.61E+04,1.62E+04,2.61E+04,2.90E+04,5.8
165.04399,1.84E+03,2.68E+03,2.62E+03,1.04E+03,1.95E+03,1.75E+03,1.33E+03,1.67E+03,2.05E+03,2.
165.04672,1.91E+03,1.73E+03,2.00E+03,7.26E+02,1.25E+03,7.35E+02,6.18E+02,1.39E+03,1.57E+03,1.
165.94576,3.83E+03,2.27E+03,3.79E+03,3.52E+03,7.22E+03,2.17E+03,2.68E+03,3.66E+03,3.40E+03,3.
165.97727,8.85E+01,7.47E+02,4.26E+02,1.47E+02,3.23E+02,3.15E+02,1.73E+01,8.57E+01,1.48E+02,2.
165.97868,8.85E+01,7.47E+02,4.26E+02,1.47E+02,3.23E+02,3.15E+02,1.73E+01,8.57E+01,1.48E+02,2.
166.04354,2.33E+03,1.76E+03,1.61E+03,1.61E+03,3.02E+03,1.90E+03,1.43E+03,1.60E+03,2.09E+03,2.
166.93259,4.61E+03,4.41E+03,3.35E+03,3.37E+03,5.79E+03,4.83E+03,2.37E+03,5.00E+03,3.59E+03,6.

166.94177,2.18E+03,3.50E+03,2.53E+03,1.14E+03,2.55E+01,6.38E+01,2.41E+03,9.20E+02,1.90E+03,2.
166.9445,2.64E+03,3.96E+02,2.15E+03,2.78E+03,3.24E+03,1.95E+03,1.84E+03,3.96E+03,1.38E+03,2.0
166.95027,1.07E+03,2.68E+03,1.63E+03,3.60E+02,1.32E+03,8.79E+02,1.14E+03,9.64E+02,1.00E+03,1.
166.97489,2.70E+03,2.08E+03,5.91E+03,5.62E+03,5.05E+03,1.91E+03,3.77E+03,3.59E+03,3.23E+03,3.
167.02263,1.47E+02,1.63E+03,4.54E+02,1.20E+03,4.06E+03,1.51E+03,4.49E+02,1.29E+02,2.80E+01,1.
167.07115,2.44E+03,1.24E+03,3.50E+01,9.51E+02,1.61E+03,1.56E+03,9.30E+02,1.64E+03,1.32E+03,1.
167.94367,1.34E+03,1.89E+03,3.50E+01,1.90E+03,3.98E+03,1.85E+02,2.38E+03,8.52E+02,1.64E+03,2.
168.04118,8.00E+00,1.14E+03,1.38E+03,2.10E+01,2.88E+03,1.01E+03,1.24E+03,1.01E+02,6.44E+02,1.
168.83587,1.12E+05,8.64E+04,4.43E+04,9.90E+04,1.35E+04,3.23E+04,8.42E+04,8.66E+04,8.86E+04,6.
168.92933,3.44E+03,2.42E+03,2.50E+03,2.10E+03,2.62E+03,2.60E+03,1.76E+03,1.86E+03,2.59E+03,3.
168.99041,5.92E+02,2.10E+03,2.88E+03,2.32E+03,4.38E+03,2.18E+03,1.56E+03,1.50E+03,1.43E+03,2.
168.99892,2.80E+03,2.99E+03,2.21E+03,1.35E+03,1.93E+03,1.61E+03,1.28E+03,2.60E+03,2.93E+03,2.
169.0502,1.92E+03,2.04E+03,9.10E+01,1.20E+03,2.26E+03,1.80E+03,1.76E+03,1.72E+03,1.08E+03,2.2
169.08664,1.71E+03,2.12E+03,1.00E+01,1.81E+03,2.42E+03,2.02E+03,1.47E+03,2.35E+03,2.15E+03,2.
170.03245,1.61E+03,1.43E+03,2.86E+03,1.22E+03,4.96E+02,2.06E+03,3.53E+02,2.66E+03,1.04E+02,2.
170.83285,1.54E+05,1.44E+05,6.90E+04,1.56E+05,2.41E+04,6.11E+04,1.34E+05,1.50E+05,1.49E+05,1.
170.89235,4.08E+03,6.84E+03,4.21E+03,2.27E+03,2.32E+03,2.03E+03,8.57E+03,2.75E+03,3.50E+03,2.
170.92829,2.01E+03,2.28E+03,1.73E+03,1.45E+03,1.86E+03,1.90E+03,1.08E+03,1.60E+03,2.10E+03,1.
170.96988,2.73E+03,9.47E+03,7.24E+03,1.58E+03,4.95E+03,8.36E+03,2.21E+03,3.27E+03,3.27E+03,6.
170.9785,5.58E+01,1.33E+03,2.43E+03,2.40E+02,7.89E+01,1.75E+03,1.83E+02,2.18E+03,2.27E+03,1.9
171.00619,3.06E+03,3.45E+03,2.02E+03,2.15E+03,4.27E+03,3.20E+03,2.67E+03,1.62E+03,2.28E+03,4.
171.06596,2.39E+04,3.16E+03,3.31E+04,1.55E+04,1.08E+04,1.21E+04,1.30E+04,3.90E+04,1.84E+04,2.
171.10234,3.34E+03,3.38E+03,1.21E+03,5.27E+03,1.88E+04,3.99E+03,2.81E+03,9.03E+03,3.55E+03,6.
171.13873,2.65E+03,3.04E+03,3.66E+03,3.16E+03,2.91E+03,1.94E+03,2.37E+03,3.20E+03,2.41E+03,3.
171.83385,7.02E+03,4.61E+03,2.86E+03,5.57E+03,1.65E+03,2.59E+03,4.83E+03,5.52E+03,6.22E+03,3.
172.06126,2.21E+03,1.99E+03,2.03E+03,1.30E+03,2.67E+03,2.27E+03,2.11E+03,1.61E+03,1.68E+03,1.
172.50945,2.21E+02,2.90E+03,5.50E+00,1.17E+03,2.48E+03,3.20E+01,1.18E+02,2.19E+03,1.60E+01,1.
172.82993,8.99E+04,6.73E+04,3.18E+04,7.70E+04,9.77E+03,2.20E+04,6.86E+04,6.73E+04,7.50E+04,5.
172.88934,1.79E+03,3.57E+03,2.77E+03,2.80E+01,5.74E+01,5.13E+00,2.32E+03,1.54E+03,1.59E+03,1.
173.00145,1.48E+03,2.81E+03,1.06E+03,8.00E+00,5.91E+02,1.34E+03,2.40E+01,1.90E+03,4.79E+02,1.
173.00881,2.33E+04,2.28E+04,1.71E+04,1.76E+04,3.91E+04,1.43E+04,1.59E+04,3.13E+04,2.14E+04,3.
173.04532,3.61E+03,2.70E+03,2.09E+03,6.44E+03,7.28E+03,4.37E+03,3.87E+03,5.50E+03,3.14E+03,6.
173.08166,1.83E+03,1.37E+03,5.63E+00,1.80E+03,2.42E+03,1.44E+03,1.37E+03,2.58E+03,1.28E+03,2.
173.118,4.34E+02,3.06E+02,5.63E+00,1.32E+03,1.81E+03,1.40E+03,4.63E+01,1.29E+03,1.48E+02,1.08
173.83091,4.85E+03,4.32E+03,3.07E+03,5.27E+03,1.26E+03,2.98E+03,4.53E+03,5.25E+03,5.88E+03,4.
174.01236,2.02E+03,1.27E+03,1.91E+03,7.73E+02,2.22E+03,9.46E+02,9.34E+02,1.85E+03,1.35E+03,1.
174.82859,2.40E+04,1.83E+04,1.31E+04,2.52E+04,4.71E+03,7.04E+03,1.67E+04,1.69E+04,2.20E+04,1.
174.95117,5.73E+03,2.45E+03,5.32E+03,5.21E+03,8.02E+03,4.81E+03,5.38E+03,7.65E+03,4.20E+03,6.
175.02455,1.50E+03,3.02E+03,2.10E+03,1.06E+03,3.89E+02,2.95E+03,1.42E+03,2.55E+03,2.25E+03,2.
175.04705,3.26E+04,1.84E+04,3.74E+04,5.09E+03,3.76E+05,1.88E+04,9.88E+03,1.90E+04,1.23E+04,3.
175.06096,1.50E+04,1.12E+04,1.65E+04,8.75E+03,5.30E+04,8.00E+03,6.95E+03,2.46E+04,2.32E+04,2.
175.82797,2.07E+03,1.81E+03,1.18E+01,1.53E+03,1.52E+02,7.14E+02,2.63E+03,2.34E+03,1.78E+03,1.
175.94951,2.68E+03,2.89E+03,3.48E+03,2.36E+03,5.40E+03,8.61E+02,3.26E+03,2.84E+03,4.66E+03,3.
176.04392,1.39E+02,6.50E+00,1.24E+02,2.21E+02,2.86E+03,1.05E+02,4.53E+01,3.10E+02,2.76E+02,1.

176.05023,1.33E+03,6.13E+02,1.56E+03,2.21E+02,7.29E+03,1.81E+03,5.62E+02,1.50E+03,2.76E+02,1.
176.06422,2.05E+03,2.06E+03,2.50E+03,1.37E+03,2.70E+03,2.23E+03,1.17E+03,2.00E+03,1.95E+03,1.
176.82553,7.56E+03,5.49E+03,4.02E+03,5.99E+03,1.54E+03,3.76E+03,5.83E+03,5.00E+03,5.95E+03,5.
176.9359,1.36E+03,1.59E+03,2.73E+03,1.39E+03,5.03E+03,1.75E+03,1.83E+03,8.07E+01,8.32E+01,2.8
176.94923,1.94E+03,1.95E+03,3.79E+03,2.68E+03,3.34E+03,2.16E+01,2.15E+03,2.48E+03,2.54E+03,1.
177.04023,2.25E+03,2.28E+03,2.41E+03,3.46E+02,1.36E+03,1.81E+03,1.48E+03,1.52E+03,1.07E+03,1.
177.05554,3.71E+03,2.72E+03,3.54E+03,3.46E+03,7.72E+03,6.60E+03,2.25E+03,3.26E+03,3.35E+03,3.
177.07658,2.34E+03,1.15E+03,2.30E+03,1.63E+03,4.54E+03,2.84E+03,1.50E+03,1.19E+03,1.51E+03,1.
177.96835,2.05E+03,1.82E+03,2.78E+02,2.80E+01,2.32E+02,3.18E+03,2.23E+02,1.75E+03,1.89E+03,2.
178.96664,1.41E+02,3.54E+03,5.35E+02,1.00E+02,6.69E+02,1.69E+03,5.18E+02,1.35E+03,1.69E+03,1.
178.97942,3.25E+03,2.84E+03,3.77E+03,2.65E+03,6.69E+02,1.67E+02,7.30E+03,2.76E+03,2.37E+03,1.
179.03482,4.25E+03,3.44E+03,3.40E+03,4.50E+03,1.35E+04,7.41E+03,2.98E+03,4.56E+03,4.34E+03,3.
179.05583,8.81E+04,9.64E+04,8.08E+04,7.13E+04,1.18E+05,1.44E+05,6.74E+04,1.06E+05,7.34E+04,9.
179.51715,2.13E+03,2.60E+03,3.10E+03,1.11E+03,1.60E+03,1.24E+03,8.15E+02,1.56E+03,1.90E+03,1.
179.99148,2.03E+03,9.32E+02,5.12E+01,1.42E+03,2.19E+03,2.23E+03,1.48E+03,1.42E+03,1.88E+03,1.
180.05917,3.54E+03,2.95E+03,3.17E+03,2.43E+03,4.22E+03,3.83E+03,1.97E+03,3.38E+03,2.82E+03,3.
180.0664,4.40E+01,1.95E+03,2.03E+03,3.56E+01,1.04E+03,2.96E+01,2.29E+03,1.06E+03,1.13E+03,8.0
180.99013,1.37E+03,2.49E+03,8.21E+02,2.43E+03,1.36E+03,1.57E+03,1.10E+03,6.80E+01,1.63E+03,1.
181.91969,2.58E+02,2.61E+03,2.10E+03,1.31E+03,1.81E+03,2.19E+03,4.74E+02,2.14E+01,1.56E+03,6.
181.92492,2.91E+03,4.05E+03,3.79E+03,9.94E+02,4.36E+02,1.46E+03,6.06E+03,2.16E+03,1.83E+03,1.
182.02246,1.31E+03,1.14E+03,8.13E+02,1.34E+03,2.56E+03,1.53E+03,1.51E+03,2.23E+03,1.58E+03,2.
182.92776,7.88E+02,1.70E+03,1.01E+03,1.18E+03,1.33E+03,3.45E+03,1.11E+03,1.12E+03,1.42E+03,2.
182.92984,1.30E+04,4.36E+03,6.36E+03,5.63E+03,6.00E+02,2.07E+03,1.95E+04,3.66E+03,1.77E+03,1.
183.06604,4.09E+02,5.17E+02,1.48E+02,9.49E+02,1.57E+03,1.22E+03,1.61E+03,1.51E+03,1.31E+03,1.
183.10246,1.23E+03,5.17E+02,1.48E+02,7.37E+02,1.78E+03,1.05E+03,1.27E+03,1.02E+03,1.13E+03,1.
183.92255,1.40E+03,3.36E+03,2.32E+03,1.66E+03,2.37E+02,6.17E+01,1.85E+03,3.65E+01,1.55E+03,1.
184.89871,1.82E+03,1.19E+03,5.98E+02,1.39E+03,2.44E+03,1.97E+03,1.18E+03,2.20E+03,1.57E+03,2.
184.92696,6.76E+03,2.11E+03,2.47E+03,2.71E+03,6.70E+01,1.51E+03,6.75E+03,2.00E+03,1.42E+03,1.
184.98562,1.13E+04,9.27E+03,9.81E+03,4.44E+04,1.81E+04,7.24E+03,1.34E+04,1.06E+04,7.92E+03,1.
185.02191,3.64E+02,1.99E+03,8.03E+02,1.11E+03,3.48E+03,1.71E+03,1.10E+03,8.76E+02,1.19E+03,1.
185.08181,2.35E+03,2.34E+03,1.18E+03,2.18E+03,2.81E+03,1.93E+03,1.45E+03,2.75E+03,1.87E+03,3.
185.11819,2.13E+03,1.05E+03,8.00E+00,1.90E+03,2.41E+03,1.29E+03,9.95E+02,2.50E+03,1.34E+03,1.
185.15456,1.08E+02,7.15E+02,1.47E+02,1.22E+02,2.00E+02,1.67E+03,6.90E+02,7.36E+02,1.60E+03,1.
186.89574,1.56E+03,2.00E+03,1.09E+03,1.84E+03,1.94E+03,1.99E+03,6.10E+01,1.98E+03,1.93E+03,2.
187.0609,1.47E+03,1.59E+03,6.50E+01,1.99E+03,2.08E+03,2.23E+03,2.05E+03,2.11E+03,1.59E+03,2.4
187.09733,1.59E+03,1.85E+03,8.02E+02,1.41E+03,2.26E+03,1.63E+03,1.02E+03,1.67E+03,1.22E+03,1.
187.96842,5.07E+03,7.81E+03,7.60E+03,4.13E+03,1.19E+03,5.21E+03,5.09E+03,2.99E+03,5.76E+03,1.
187.98098,1.76E+03,1.24E+03,2.02E+03,1.60E+03,2.78E+02,1.51E+03,2.40E+03,4.51E+02,7.73E+01,1.
188.0562,1.07E+04,1.99E+03,3.34E+04,2.30E+03,1.01E+04,2.52E+03,1.65E+03,5.47E+03,1.53E+03,4.8
188.89434,1.83E+03,1.71E+03,3.55E+01,8.89E+02,3.90E+01,2.61E+01,1.03E+02,1.69E+03,1.18E+03,6.
189.04034,1.39E+03,8.75E+02,1.52E+03,1.60E+03,2.33E+03,2.80E+03,1.99E+03,9.98E+02,1.72E+03,1.
189.05736,1.82E+03,1.47E+03,1.41E+03,1.24E+02,1.19E+03,2.38E+03,4.40E+01,1.03E+02,5.49E+03,3.
189.05949,1.82E+03,5.30E+02,2.64E+03,1.24E+02,1.97E+03,7.01E+02,4.40E+01,1.03E+02,4.69E+02,3.
189.07684,1.89E+03,1.17E+03,2.11E+03,1.22E+03,1.09E+04,1.93E+03,4.40E+01,3.08E+03,1.20E+03,1.

190.96017,1.46E+02,1.36E+03,1.14E+03,3.70E+02,8.70E+01,1.32E+03,3.48E+02,1.88E+02,1.15E+02,1.
191.01951,3.13E+05,2.64E+05,2.21E+05,1.85E+05,6.88E+05,2.76E+05,1.30E+05,2.92E+05,2.12E+05,3.
191.05609,6.62E+02,8.60E+02,1.58E+03,3.25E+02,7.78E+02,1.38E+03,9.70E+02,1.26E+03,3.77E+01,9.
192.02297,1.03E+04,7.77E+03,8.24E+03,6.40E+03,2.38E+04,7.56E+03,4.75E+03,9.52E+03,7.12E+03,1.
193.02406,3.23E+03,2.99E+03,3.20E+03,2.73E+03,4.74E+03,2.86E+03,1.98E+03,3.34E+03,2.20E+03,2.
193.03537,8.50E+02,1.30E+03,1.18E+03,9.26E+02,1.78E+03,1.64E+03,1.25E+03,1.68E+03,6.38E+01,1.
193.0506,7.55E+04,7.20E+04,1.12E+05,4.26E+04,5.08E+04,7.81E+04,8.55E+04,6.90E+04,1.08E+05,4.1
193.07175,8.50E+04,1.15E+05,7.87E+04,5.51E+04,1.24E+05,1.72E+05,9.88E+04,1.11E+05,1.16E+05,7.
194.05394,4.23E+03,4.91E+03,6.44E+03,2.66E+03,4.21E+03,4.55E+03,4.49E+03,4.34E+03,7.28E+03,3.
194.07493,4.72E+03,5.43E+03,3.10E+03,2.27E+03,4.79E+03,4.86E+03,4.41E+03,4.67E+03,4.03E+03,3.
194.92762,1.12E+03,1.87E+03,1.08E+02,1.32E+03,1.98E+03,1.88E+03,7.51E+02,2.75E+03,3.99E+01,2.
194.93691,1.65E+03,1.99E+03,1.08E+02,1.37E+03,1.21E+02,1.00E+00,1.59E+03,1.03E+03,1.51E+03,5.
194.94637,1.82E+03,7.52E+01,3.46E+03,3.32E+03,9.40E+03,9.73E+02,3.91E+03,5.61E+02,1.91E+03,4.
195.0509,2.47E+04,3.83E+04,2.99E+04,2.63E+04,3.14E+04,6.63E+04,2.81E+04,1.89E+04,3.10E+04,2.1
195.06605,1.53E+03,2.01E+03,1.70E+02,1.26E+03,3.43E+03,2.13E+03,1.25E+03,1.49E+03,1.45E+03,1.
195.0759,2.35E+03,2.05E+01,1.70E+02,5.00E+01,1.99E+03,1.44E+03,1.60E+03,2.09E+03,9.52E+02,9.7
195.85502,9.78E+04,5.62E+04,9.06E+04,4.25E+04,3.93E+03,8.66E+03,8.14E+04,2.14E+04,5.04E+04,1.
196.05418,1.90E+03,2.80E+03,2.38E+03,1.52E+03,1.50E+03,3.52E+03,1.06E+03,1.69E+03,2.22E+03,8.
197.08186,6.49E+02,1.67E+03,1.09E+02,1.86E+03,1.59E+03,1.25E+03,8.95E+02,1.26E+03,1.27E+03,1.
197.11821,8.89E+02,1.91E+03,1.09E+02,1.81E+03,2.21E+03,1.09E+03,1.12E+03,1.45E+03,1.70E+03,1.
197.8519,1.28E+05,6.79E+04,1.08E+05,4.71E+04,6.40E+03,1.15E+04,1.08E+05,2.89E+04,5.79E+04,1.3
197.9116,2.64E+03,2.58E+03,1.82E+03,3.19E+02,2.31E+02,6.68E+01,2.51E+03,6.50E+00,1.65E+03,1.3
198.85301,4.88E+03,3.87E+03,4.75E+03,3.29E+03,6.65E+02,1.74E+03,4.50E+03,1.84E+03,3.48E+03,1.
198.90751,3.28E+03,1.75E+03,1.05E+03,2.60E+03,6.65E+02,1.70E+02,2.68E+03,1.45E+03,1.09E+03,9.
199.09756,1.85E+03,2.12E+03,8.20E+01,2.56E+03,2.70E+03,1.79E+03,1.72E+03,3.83E+03,2.20E+03,2.
199.13392,4.80E+01,1.10E+03,1.47E+03,8.10E+01,1.48E+03,1.01E+03,8.49E+02,1.63E+03,1.03E+03,1.
199.17024,1.32E+03,1.33E+03,1.00E+03,1.16E+03,1.35E+03,1.17E+03,1.22E+03,1.32E+03,1.51E+03,1.
199.84891,3.14E+04,1.52E+04,3.03E+04,1.23E+04,1.57E+03,3.42E+03,2.40E+04,1.03E+04,1.65E+04,4.
199.85068,2.08E+04,1.16E+04,2.05E+04,8.47E+03,1.53E+03,2.80E+03,1.72E+04,6.86E+03,1.06E+04,4.
200.8501,2.59E+03,1.84E+03,3.76E+03,2.41E+03,4.11E+02,1.27E+03,4.22E+03,1.54E+03,1.85E+03,1.2
200.89365,3.67E+03,4.83E+03,3.65E+03,6.87E+03,5.88E+03,9.39E+03,2.65E+03,2.89E+03,5.77E+03,2.
201.07669,1.69E+03,1.95E+03,1.08E+02,2.34E+03,2.44E+03,1.97E+03,1.69E+03,2.53E+03,1.68E+03,2.
201.1133,1.56E+03,1.31E+03,7.50E+01,1.97E+03,2.77E+03,2.07E+03,1.14E+03,2.91E+03,1.66E+03,2.6
201.84768,1.49E+04,6.45E+03,1.17E+04,6.40E+03,6.61E+01,2.66E+03,1.25E+04,6.41E+03,8.37E+03,3.
201.92611,1.28E+03,8.22E+01,8.30E+01,1.16E+02,2.37E+03,1.47E+03,1.24E+02,7.77E+02,1.11E+03,1.
201.98669,1.82E+03,2.73E+03,2.21E+03,2.44E+03,2.18E+03,3.65E+03,9.38E+02,2.83E+03,2.35E+03,1.
202.89077,3.33E+03,4.21E+03,3.11E+03,5.01E+03,5.41E+03,7.72E+03,2.75E+03,2.65E+03,4.18E+03,1.
202.98518,1.76E+03,1.98E+03,1.72E+03,1.11E+03,1.98E+03,2.04E+03,9.51E+02,2.10E+02,2.67E+03,1.
203.05609,1.54E+03,1.55E+03,3.46E+02,1.44E+03,1.33E+03,1.59E+03,1.21E+03,1.92E+03,1.90E+03,1.
203.08268,2.01E+03,8.90E+01,3.46E+02,1.52E+03,3.42E+01,1.48E+03,3.20E+01,5.44E+01,1.40E+03,1.
203.09248,1.33E+03,2.02E+03,3.46E+02,1.18E+03,2.32E+03,1.72E+03,1.20E+03,1.51E+03,1.15E+03,1.
203.84487,3.40E+03,1.89E+03,3.75E+03,2.30E+03,3.90E+02,1.56E+03,1.86E+03,1.62E+03,1.51E+03,1.
203.95513,2.92E+03,2.79E+03,2.60E+03,2.77E+03,4.16E+02,2.92E+03,2.93E+03,1.90E+02,1.62E+03,9.
203.98444,8.98E+01,1.80E+03,8.57E+01,9.87E+02,1.59E+03,2.94E+03,1.48E+03,1.95E+03,1.01E+03,5.

204.88935,1.65E+03,2.08E+03,2.22E+03,1.80E+03,1.97E+03,3.84E+03,1.22E+03,2.05E+03,2.36E+03,1.
204.97587,3.20E+02,1.02E+03,1.19E+03,9.92E+01,2.02E+02,2.69E+02,1.01E+03,1.65E+03,1.82E+03,1.
204.97871,3.20E+02,4.62E+02,3.00E+02,9.92E+01,2.20E+03,2.69E+02,7.08E+02,3.04E+02,5.26E+02,1.
205.03535,5.60E+03,5.76E+03,5.67E+03,4.82E+03,6.41E+03,3.96E+03,5.03E+03,8.49E+03,5.42E+03,7.
205.15987,5.74E+02,5.73E+02,5.94E+02,8.66E+02,5.91E+02,1.01E+03,8.13E+02,6.35E+02,8.06E+02,8.
206.03853,1.15E+03,7.00E+02,1.00E+03,2.80E+01,2.09E+02,4.37E+02,1.44E+03,5.24E+02,2.50E+00,3.
206.08245,3.98E+03,7.00E+02,1.94E+03,1.23E+03,2.72E+03,1.60E+03,1.55E+03,1.68E+03,6.10E+02,3.
207.0512,1.97E+03,3.28E+03,9.61E+02,1.08E+03,3.18E+03,1.09E+04,1.31E+03,1.24E+03,7.08E+02,1.6
207.0874,2.67E+03,2.54E+03,2.81E+03,1.21E+03,4.15E+03,2.11E+03,1.30E+03,2.45E+03,1.45E+03,2.3
207.10274,2.01E+03,1.22E+03,1.90E+02,4.34E+03,3.28E+03,1.76E+03,1.96E+03,8.01E+03,1.68E+03,1.
207.93547,4.60E+03,2.94E+03,1.76E+03,1.38E+03,1.70E+03,1.63E+03,1.34E+03,1.44E+03,1.69E+03,1.
208.84841,2.33E+03,1.64E+03,6.30E+02,1.71E+03,1.14E+02,5.84E+02,2.14E+03,1.53E+02,1.62E+02,3.
208.94343,9.26E+03,9.56E+03,7.42E+03,5.86E+03,1.33E+04,9.62E+03,4.92E+03,1.03E+04,8.85E+03,1.
208.98566,5.02E+03,6.18E+03,4.02E+03,5.34E+03,5.03E+03,2.54E+03,5.93E+03,9.64E+03,7.45E+03,9.
209.03017,1.95E+05,1.61E+05,1.34E+05,2.69E+05,4.32E+05,3.17E+05,1.09E+05,2.01E+05,8.26E+04,1.
209.04136,3.66E+02,1.83E+03,3.72E+03,1.31E+03,2.59E+02,1.11E+02,2.71E+03,2.68E+03,2.82E+03,1.
209.06662,1.03E+04,1.27E+04,1.41E+04,9.63E+03,1.57E+04,2.20E+04,8.04E+03,7.94E+03,8.21E+03,1.
209.09463,6.25E+04,4.04E+03,5.96E+04,5.56E+04,6.69E+03,2.14E+04,2.03E+04,6.98E+04,4.59E+04,9.
209.11829,2.08E+03,1.87E+03,1.02E+02,3.50E+03,7.67E+03,2.28E+03,1.35E+03,7.71E+03,3.51E+03,1.
210.03368,1.59E+04,1.42E+04,9.27E+03,1.03E+04,1.72E+04,1.21E+04,7.16E+03,1.57E+04,7.34E+03,8.
210.07024,2.95E+02,1.00E+03,9.29E+02,1.05E+03,1.47E+03,1.60E+03,9.85E+02,1.05E+03,1.32E+03,1.
210.94039,4.97E+03,5.88E+03,3.82E+03,4.01E+03,6.35E+03,3.96E+03,2.90E+03,5.60E+03,4.72E+03,6.
210.94959,2.58E+04,8.07E+03,3.27E+04,1.18E+04,8.10E+00,7.30E+00,9.75E+04,3.85E+03,3.64E+03,1.
210.98295,1.84E+03,1.71E+03,1.49E+03,1.95E+03,3.79E+03,7.30E+00,1.87E+03,2.71E+03,3.46E+03,2.
211.00979,2.40E+03,6.28E+01,5.45E+01,5.79E+02,1.72E+03,7.30E+00,1.32E+02,2.39E+03,4.88E+01,1.
211.03455,4.08E+03,4.22E+03,4.14E+03,4.04E+03,5.17E+03,4.76E+03,3.36E+03,4.02E+03,1.31E+03,2.
211.09764,1.85E+03,1.68E+03,1.76E+03,9.74E+02,1.91E+03,1.31E+03,1.49E+03,1.72E+03,1.76E+03,1.
211.13414,3.80E+03,1.99E+03,2.19E+03,7.02E+03,2.66E+04,1.94E+03,1.85E+03,1.71E+04,3.32E+03,1.
211.94123,2.19E+03,9.67E+02,1.88E+02,5.15E+02,6.92E+02,9.90E+02,6.92E+02,1.27E+03,9.49E+02,1.
211.94633,3.49E+04,1.21E+04,3.80E+04,1.42E+04,3.85E+02,2.92E+03,1.11E+05,3.69E+03,4.52E+03,2.
211.95386,1.95E+03,1.76E+03,3.11E+03,1.56E+03,1.93E+03,1.13E+03,5.26E+03,1.22E+03,1.20E+03,1.
212.07509,4.61E+03,5.57E+03,5.39E+03,5.06E+03,5.60E+03,5.32E+03,3.89E+03,4.88E+03,4.91E+03,4.
212.13735,6.86E+00,3.50E+00,2.56E+00,1.26E+03,2.65E+03,8.25E+00,1.44E+00,1.96E+03,2.20E+00,1.
212.93893,3.53E+03,4.26E+03,1.79E+03,2.06E+03,5.38E+03,3.28E+03,2.23E+03,3.17E+03,2.72E+03,5.
212.98902,1.37E+04,2.75E+04,1.78E+04,7.07E+03,1.03E+04,1.15E+04,8.10E+03,2.30E+04,1.58E+04,1.
213.00152,2.19E+03,1.29E+03,1.19E+03,1.03E+03,1.39E+02,2.07E+03,1.20E+03,1.86E+03,1.71E+03,1.
213.07668,1.81E+03,1.97E+03,1.43E+03,1.44E+03,1.89E+03,1.03E+03,1.12E+03,1.48E+03,1.19E+03,1.
213.11313,1.88E+03,2.00E+03,1.59E+03,2.19E+03,3.54E+03,2.03E+03,2.04E+03,3.33E+03,1.81E+03,1.
213.14957,2.20E+03,2.44E+03,1.64E+02,1.81E+03,4.13E+03,3.15E+03,1.43E+03,3.28E+03,2.01E+03,3.
213.18578,5.73E+01,5.72E+02,2.30E+01,1.21E+03,1.03E+03,6.13E+02,7.20E+02,6.70E+01,7.90E+01,6.
213.99004,2.16E+03,2.66E+03,2.20E+03,1.04E+03,1.73E+03,1.43E+03,1.04E+03,2.00E+03,2.39E+03,1.
214.07139,1.44E+02,2.83E+02,2.76E+02,5.80E+02,3.42E+02,1.44E+02,9.51E+02,2.62E+02,3.46E+02,3.
214.93541,6.07E+02,1.65E+03,1.20E+01,2.57E+02,1.69E+03,1.50E+03,5.50E+01,2.20E+01,1.02E+03,1.
214.98671,2.22E+03,3.21E+03,1.75E+03,1.42E+03,2.67E+03,1.44E+03,1.47E+03,2.15E+03,2.39E+03,2.

215.03269,5.06E+04,7.52E+04,2.07E+04,1.32E+05,1.38E+05,1.38E+05,3.34E+04,1.19E+05,7.64E+04,1.
215.09276,3.64E+03,4.61E+03,2.85E+02,4.99E+03,6.39E+03,4.43E+03,2.75E+03,6.65E+03,4.21E+03,7.
215.12894,2.33E+03,1.74E+03,1.50E+01,1.41E+03,2.45E+03,1.19E+03,6.06E+02,1.60E+03,1.30E+03,1.
215.9948,1.21E+02,1.31E+03,3.16E+03,8.20E+01,1.67E+03,1.53E+03,7.53E+02,1.56E+03,1.15E+03,1.2
216.03635,2.40E+03,2.84E+03,1.46E+03,4.80E+03,4.05E+03,4.25E+03,2.21E+03,3.76E+03,2.77E+03,4.
216.99177,3.40E+02,2.06E+03,1.33E+03,3.51E+02,1.86E+03,1.82E+03,1.80E+02,1.29E+03,1.40E+03,1.
217.02978,1.18E+04,1.55E+04,5.71E+03,2.41E+04,3.13E+04,3.05E+04,1.03E+04,2.51E+04,1.65E+04,2.
217.03664,2.18E+03,1.20E+03,1.19E+03,1.25E+03,1.47E+03,1.04E+03,7.88E+02,1.05E+03,1.19E+03,1.
217.10833,1.91E+03,1.89E+03,7.67E+02,1.34E+03,2.14E+03,1.62E+03,1.87E+03,1.40E+03,1.91E+03,2.
217.97939,2.06E+03,2.36E+03,3.35E+03,4.49E+02,1.34E+03,1.02E+03,1.74E+02,3.03E+01,1.80E+01,1.
217.9904,6.86E+02,1.64E+03,2.30E+03,4.49E+02,2.65E+03,4.63E+02,1.74E+02,3.03E+01,9.50E+02,1.0
218.03302,1.38E+03,1.35E+03,5.51E+02,1.34E+03,1.95E+03,1.87E+03,1.74E+02,1.74E+03,1.61E+03,2.
218.04918,1.93E+03,1.71E+03,1.87E+03,4.80E+01,3.93E+03,1.85E+03,1.53E+03,1.79E+03,1.16E+03,2.
218.10329,1.31E+02,1.10E+03,1.55E+03,3.30E+02,2.40E+03,1.26E+03,1.33E+03,1.42E+02,5.04E+02,1.
219.97846,1.54E+03,2.02E+03,2.60E+03,1.30E+03,1.63E+03,1.91E+03,2.52E+03,2.40E+03,1.12E+03,1.
221.06682,1.88E+03,2.02E+03,1.46E+02,1.45E+03,1.16E+03,2.10E+03,4.50E+01,1.62E+03,2.03E+03,1.
221.10322,2.07E+03,2.31E+03,2.28E+03,4.78E+02,1.57E+03,1.53E+03,1.45E+03,1.45E+03,2.10E+03,1.
222.87407,9.69E+03,4.89E+03,1.43E+04,3.08E+03,3.51E+02,1.31E+03,1.10E+04,1.71E+03,3.97E+03,9.
222.95919,1.57E+03,2.01E+03,5.40E+02,1.39E+03,1.41E+03,1.72E+03,2.15E+02,1.32E+03,6.53E+02,1.
223.00244,2.17E+03,2.30E+03,5.00E+00,9.98E+02,2.79E+03,2.95E+03,1.95E+03,1.83E+03,2.98E+03,2.
223.04591,1.44E+04,1.64E+04,1.38E+04,1.29E+04,2.09E+04,2.52E+04,1.37E+04,1.05E+04,1.27E+04,1.
223.06107,1.61E+03,1.88E+03,2.18E+03,1.30E+03,4.02E+03,2.11E+03,4.20E+01,1.32E+03,2.00E+03,1.
223.13404,4.46E+01,1.09E+03,7.55E+02,1.17E+03,2.27E+03,2.01E+03,1.90E+03,2.50E+03,1.49E+03,9.
223.93052,1.49E+03,2.30E+03,3.00E+03,1.75E+03,1.12E+03,3.26E+03,1.50E+03,1.24E+02,2.97E+03,1.
224.00076,1.91E+03,1.14E+03,5.30E+02,1.16E+03,1.94E+03,1.47E+03,7.14E+02,1.43E+03,1.67E+03,2.
224.04952,2.27E+03,2.16E+03,2.24E+03,1.54E+03,2.77E+03,3.02E+03,1.48E+03,2.17E+03,1.64E+03,2.
224.87114,6.21E+03,5.62E+03,1.20E+04,1.98E+02,3.53E+02,2.72E+02,9.88E+03,2.00E+03,3.75E+03,1.
224.93854,1.77E+03,1.23E+03,1.89E+03,1.08E+03,1.82E+03,1.99E+03,1.70E+03,4.64E+02,1.63E+03,3.
225.00063,1.15E+03,1.48E+03,6.46E+02,2.24E+02,1.08E+03,1.61E+03,1.25E+03,1.10E+03,1.39E+03,2.
225.06181,6.50E+02,1.60E+02,1.54E+03,4.19E+02,7.26E+02,1.35E+03,1.15E+02,5.48E+02,2.47E+02,1.
225.11344,2.55E+03,2.50E+03,1.12E+03,5.40E+03,5.08E+03,2.93E+03,2.22E+03,1.12E+04,3.96E+03,2.
225.92668,7.23E+03,3.51E+03,4.84E+03,2.64E+03,1.84E+03,2.12E+03,1.27E+04,1.57E+03,2.59E+03,7.
225.9703,2.31E+03,1.59E+03,3.57E+03,6.31E+02,1.30E+03,1.30E+03,5.63E+01,1.23E+03,1.67E+03,1.3
226.87017,3.85E+03,2.45E+03,4.44E+03,1.34E+03,3.81E+02,5.98E+02,2.61E+03,7.41E+02,6.31E+02,1.
226.95403,2.68E+03,3.33E+03,2.03E+03,1.84E+03,2.72E+03,2.22E+03,1.92E+03,3.10E+03,2.71E+03,2.
226.99676,6.21E+03,6.34E+03,4.16E+03,4.83E+03,3.50E+03,2.36E+03,5.64E+03,9.07E+03,6.94E+03,9.
227.09267,1.61E+03,1.50E+03,1.01E+02,1.80E+03,2.62E+03,1.56E+03,9.86E+02,1.90E+03,2.08E+03,2.
227.12893,5.41E+03,3.52E+03,5.83E+03,6.92E+03,1.63E+04,7.21E+03,3.24E+03,2.33E+04,6.18E+03,5.
227.20178,2.63E+03,2.01E+03,2.24E+03,1.98E+03,2.37E+03,5.10E+01,1.79E+03,2.99E+03,1.09E+03,2.
227.91274,2.30E+03,1.89E+03,2.63E+03,1.79E+03,6.44E+00,1.85E+03,2.30E+03,3.87E+01,2.56E+03,9.
227.96689,2.25E+03,1.90E+03,5.59E+02,2.98E+02,1.44E+03,1.01E+03,1.49E+02,1.75E+03,1.26E+03,1.
228.08765,1.56E+03,8.98E+02,4.36E+02,1.27E+02,7.25E+01,1.79E+03,3.02E+02,1.35E+02,1.89E+03,1.
228.13243,2.06E+03,7.47E+01,4.36E+02,1.27E+02,2.38E+03,1.88E+03,1.12E+03,2.87E+03,1.53E+03,1.
228.93613,7.20E+01,7.47E+01,4.36E+02,2.04E+03,2.32E+03,1.35E+03,1.83E+02,4.26E+02,1.15E+03,2.

228.95107,5.27E+02,1.60E+03,8.10E+02,1.19E+03,1.40E+03,1.52E+03,9.25E+02,2.17E+03,1.15E+03,1.
228.96682,3.61E+02,1.58E+03,1.37E+03,2.25E+02,7.64E+02,1.16E+03,1.45E+03,1.53E+03,1.62E+03,1.
228.97565,6.31E+03,5.58E+03,5.58E+03,3.57E+03,5.58E+03,3.60E+03,3.29E+03,5.80E+03,4.02E+03,3.
228.99351,2.26E+03,1.88E+03,2.46E+03,1.53E+03,1.50E+03,2.00E+03,2.40E+03,3.44E+03,2.09E+03,2.
229.0121,2.09E+03,2.09E+03,2.36E+03,1.74E+03,4.82E+03,2.38E+03,1.22E+03,2.46E+03,1.14E+03,2.7
229.02326,2.85E+03,2.30E+03,6.15E+03,2.15E+03,1.78E+04,2.35E+03,3.60E+03,1.20E+03,3.13E+03,1.
229.04834,4.32E+04,4.09E+04,1.45E+04,3.58E+04,8.68E+04,6.52E+04,2.78E+04,5.54E+04,5.23E+04,4.
229.07162,2.09E+03,1.52E+03,6.24E+01,1.86E+03,1.76E+03,1.81E+03,1.54E+03,1.68E+03,1.12E+03,1.
229.10818,1.47E+03,2.23E+03,6.24E+01,2.34E+03,2.75E+03,1.43E+03,1.56E+03,2.35E+03,1.38E+03,2.
229.14467,1.56E+02,1.76E+03,6.24E+01,1.53E+03,2.20E+03,1.62E+03,1.72E+03,2.52E+03,1.78E+03,2.
229.90969,1.41E+03,1.51E+03,1.84E+03,2.31E+03,3.56E+02,2.78E+03,1.83E+03,1.38E+01,1.31E+03,1.
229.92067,1.75E+03,1.68E+03,2.07E+03,1.20E+03,1.70E+03,1.64E+03,7.60E+01,1.38E+01,1.25E+03,1.
230.0519,2.45E+03,2.52E+03,2.37E+03,1.69E+03,4.04E+03,4.18E+03,1.73E+03,4.08E+03,3.62E+03,2.4
230.90476,1.30E+03,1.91E+03,1.80E+03,9.33E+01,3.19E+03,2.95E+03,1.91E+03,4.74E+01,1.92E+03,5.
230.93364,5.25E+02,4.41E+01,2.41E+02,9.33E+01,3.11E+03,9.50E+01,3.37E+02,4.74E+01,7.23E+01,2.
230.99981,2.02E+03,2.92E+03,2.26E+03,1.06E+03,1.15E+03,1.43E+03,1.45E+03,2.31E+03,1.99E+03,1.
231.01139,5.34E+03,2.09E+03,5.27E+03,2.33E+03,1.26E+03,1.63E+03,4.60E+03,2.24E+03,1.67E+03,9.
231.04563,1.06E+04,1.01E+04,5.56E+03,7.79E+03,1.95E+04,1.47E+04,8.74E+03,1.52E+04,1.23E+04,9.
231.05268,1.11E+03,1.18E+03,6.39E+01,6.50E+00,1.81E+02,1.01E+03,1.17E+03,1.75E+02,1.64E+03,5.
231.08754,2.04E+03,1.51E+03,6.39E+01,1.84E+03,3.25E+03,2.31E+03,1.35E+03,1.78E+03,1.26E+03,2.
231.12394,1.31E+03,1.45E+03,6.39E+01,1.60E+03,1.38E+03,1.31E+03,2.36E+03,1.56E+03,1.27E+03,1.
231.91766,1.69E+03,1.66E+03,3.79E+02,1.15E+03,1.59E+03,1.75E+03,1.57E+02,1.90E+02,1.77E+03,1.
231.99543,1.53E+03,2.16E+03,1.83E+03,1.58E+03,1.75E+03,2.55E+02,1.27E+03,3.14E+03,2.58E+03,1.
232.04871,1.34E+03,1.30E+03,4.23E+02,1.40E+03,2.03E+03,3.17E+03,1.77E+03,1.32E+03,2.00E+03,1.
232.09549,1.94E+03,3.54E+03,2.49E+03,1.71E+03,1.43E+03,1.84E+03,1.43E+03,1.55E+03,2.17E+03,6.
232.90168,1.18E+02,1.93E+03,2.15E+03,2.30E+03,2.63E+03,1.93E+03,2.83E+03,2.08E+03,1.30E+03,4.
232.96126,2.79E+03,2.65E+03,1.96E+03,1.14E+03,8.58E+01,1.66E+03,9.78E+02,1.70E+03,1.98E+03,2.
233.10317,3.27E+03,4.40E+03,7.67E+03,3.02E+03,2.09E+03,6.35E+03,1.19E+04,5.94E+03,6.37E+03,2.
233.15498,9.72E+02,4.96E+01,6.49E+01,6.51E+01,9.40E+01,1.16E+02,1.41E+02,9.00E+02,1.25E+02,1.
233.60477,9.72E+02,1.55E+03,1.83E+03,1.62E+03,2.30E+01,1.49E+03,2.37E+03,1.20E+03,2.70E+03,1.
233.95588,8.43E+02,8.33E+02,1.20E+02,9.08E+01,5.10E+01,9.09E+02,5.99E+01,4.74E+02,1.36E+03,1.
233.95994,1.60E+03,1.39E+03,1.64E+03,1.03E+03,1.48E+03,1.52E+03,5.99E+01,9.88E+02,1.59E+03,1.
234.46064,1.51E+03,1.69E+02,1.60E+03,4.00E+02,8.80E+01,3.83E+02,1.84E+02,6.22E+02,1.05E+03,2.
234.90021,8.79E+02,1.56E+03,7.28E+02,4.00E+02,1.11E+03,1.87E+03,1.84E+02,1.12E+02,1.29E+03,1.
234.95932,1.61E+03,2.01E+03,1.16E+03,1.54E+03,1.09E+02,4.57E+02,9.72E+02,1.85E+03,1.08E+03,1.
235.04631,2.53E+03,2.70E+03,2.60E+03,1.98E+03,1.97E+03,2.60E+03,2.46E+03,1.64E+03,1.98E+03,1.
235.11898,2.03E+03,3.14E+03,3.62E+03,1.53E+03,1.85E+03,3.35E+03,2.49E+03,2.43E+03,3.16E+03,2.
235.86772,3.05E+03,2.10E+03,2.73E+03,2.04E+01,1.51E+02,1.78E+00,5.85E+03,3.50E+01,6.00E+01,2.
236.00506,2.30E+04,1.93E+04,2.59E+04,1.33E+04,4.15E+03,4.08E+03,3.03E+04,2.03E+04,2.26E+04,1.
236.05685,3.28E+01,5.07E+02,5.58E+02,9.60E+02,6.85E+03,1.56E+03,1.42E+03,1.43E+03,5.84E+02,1.
236.87091,2.94E+03,9.72E+02,2.33E+03,5.80E+01,4.30E+02,2.28E+03,2.87E+03,7.95E+01,1.35E+03,2.
237.00841,2.56E+03,1.97E+03,1.93E+03,5.80E+01,2.48E+02,1.97E+02,2.61E+03,1.17E+03,2.03E+03,1.
237.06179,2.60E+03,2.72E+03,3.85E+03,1.86E+03,3.76E+03,3.48E+03,1.64E+03,3.08E+03,2.60E+03,3.
238.09553,2.78E+03,2.79E+03,2.70E+03,2.86E+03,3.20E+03,1.65E+03,1.81E+02,5.76E+03,3.37E+01,5.

238.59685,5.91E+02,3.87E+02,3.87E+02,5.46E+02,9.20E+00,9.75E+00,1.81E+02,1.53E+03,3.37E+01,2.
238.8676,1.16E+03,1.54E+03,3.87E+02,1.53E+03,1.33E+03,2.40E+03,1.81E+02,2.74E+03,1.72E+03,3.4
239.05604,7.02E+05,6.05E+05,5.34E+05,7.94E+05,1.66E+06,1.23E+06,3.41E+05,6.21E+05,6.79E+05,5.
239.12901,1.00E+01,5.28E+02,8.85E+02,1.29E+03,1.53E+02,9.25E+00,1.40E+03,1.72E+03,1.18E+03,5.
240.05983,4.68E+04,3.69E+04,3.18E+04,5.51E+04,1.43E+05,9.96E+04,1.92E+04,4.20E+04,4.71E+04,3.
240.1111,2.28E+03,4.27E+03,4.32E+03,4.85E+03,2.61E+03,4.39E+03,4.98E+03,3.83E+03,5.39E+03,2.8
240.61285,1.77E+03,2.31E+03,1.40E+03,1.02E+03,2.06E+03,1.64E+03,1.74E+03,9.14E+02,1.44E+03,2.
240.95486,2.26E+03,2.62E+03,1.04E+02,1.02E+02,2.20E+01,1.80E+01,9.78E+02,1.54E+03,9.31E+02,2.
241.01211,2.86E+04,2.35E+04,1.29E+04,2.55E+04,1.84E+04,1.12E+04,2.37E+04,4.66E+04,3.46E+04,4.
241.06088,2.97E+03,3.75E+03,4.42E+03,4.29E+03,7.70E+03,5.46E+03,1.25E+03,3.28E+03,3.60E+03,2.
241.07219,1.93E+03,3.46E+01,2.07E+01,8.77E+02,1.42E+03,1.40E+03,5.50E+01,1.37E+03,1.26E+03,1.
241.10857,1.81E+03,2.25E+03,2.07E+01,3.04E+03,3.38E+03,2.56E+03,1.50E+03,3.34E+03,2.92E+03,3.
241.14494,1.33E+03,1.57E+03,2.07E+01,1.11E+03,1.99E+03,1.84E+03,1.95E+03,2.03E+03,1.96E+03,1.
241.21779,1.52E+03,1.71E+03,2.51E+03,1.42E+03,1.69E+03,8.40E+02,9.62E+02,2.10E+03,1.58E+03,2.
242.01576,1.64E+02,1.84E+03,2.52E+03,2.13E+03,1.87E+03,2.20E+03,1.30E+03,3.48E+03,2.80E+03,2.
242.05189,2.73E+04,2.91E+04,1.50E+04,2.14E+04,1.60E+04,2.33E+04,2.47E+04,2.14E+04,2.03E+04,1.
242.96101,1.98E+03,8.33E+02,1.94E+02,1.78E+03,1.55E+03,1.35E+03,5.30E+01,2.16E+03,2.08E+02,1.
243.00937,7.09E+03,6.51E+03,5.15E+03,7.87E+03,5.48E+03,2.04E+03,5.93E+03,1.30E+04,8.98E+03,1.
243.05523,2.56E+03,2.94E+03,1.85E+03,1.31E+03,2.87E+02,1.38E+03,2.31E+03,1.43E+03,2.13E+03,1.
243.06275,1.63E+03,1.38E+03,1.47E+03,8.43E+02,2.49E+03,1.70E+03,2.36E+03,1.69E+03,9.63E+02,1.
243.0876,2.29E+03,1.84E+03,5.43E+02,1.57E+03,1.65E+03,1.84E+03,1.51E+03,1.80E+03,1.69E+03,1.9
243.12388,2.27E+03,2.24E+03,5.43E+02,2.82E+03,3.75E+03,3.41E+03,1.23E+03,4.23E+03,2.18E+03,3.
243.16049,1.69E+03,1.56E+03,5.43E+02,2.28E+03,2.28E+03,2.45E+03,1.15E+03,2.39E+03,1.60E+03,2.
243.93623,2.70E+03,2.14E+03,2.27E+03,2.26E+03,3.38E+03,1.83E+03,2.02E+03,2.39E+03,2.44E+03,2.
244.01276,1.50E+03,1.02E+02,1.85E+03,1.64E+03,1.55E+03,1.56E+02,1.23E+02,1.13E+03,1.60E+03,1.
244.023,1.25E+03,1.02E+02,3.03E+03,1.29E+03,3.17E+03,1.56E+02,1.22E+03,1.32E+03,1.50E+00,2.02
244.92026,1.76E+03,1.94E+03,2.27E+03,2.03E+03,1.03E+02,3.93E+02,7.50E+01,3.94E+03,2.23E+03,1.
245.01555,2.73E+03,2.74E+03,1.90E+03,1.42E+03,2.45E+03,1.46E+03,1.71E+03,3.61E+03,1.92E+03,1.
245.04326,1.61E+04,7.66E+03,2.86E+04,1.41E+04,5.45E+04,2.90E+03,1.16E+04,1.78E+04,1.48E+04,2.
245.10312,1.78E+03,2.87E+03,2.46E+01,2.94E+03,3.59E+03,2.20E+03,1.86E+03,3.11E+03,2.14E+03,3.
245.13963,7.62E+01,3.36E+03,2.46E+01,1.61E+03,1.60E+03,1.29E+03,1.44E+03,7.32E+02,1.20E+03,1.
245.93317,1.89E+03,1.83E+03,2.10E+03,1.70E+03,2.78E+03,1.90E+03,1.71E+03,3.05E+03,2.83E+03,2.
246.04699,1.34E+03,5.73E+02,1.67E+03,2.07E+03,2.10E+03,9.13E+01,1.09E+03,1.58E+03,1.62E+03,1.
246.09304,1.27E+02,5.20E+01,2.56E+03,3.59E+02,6.98E+02,7.54E+02,6.77E+01,4.99E+02,6.43E+01,1.
246.09883,1.97E+03,5.20E+01,2.18E+03,6.90E+01,1.62E+03,1.76E+03,6.77E+01,5.90E+01,6.43E+01,1.
246.9172,2.03E+03,1.59E+03,1.90E+03,1.43E+03,1.28E+03,1.93E+03,1.68E+03,2.90E+03,2.00E+03,1.4
246.93608,2.43E+02,3.22E+03,4.74E+02,1.86E+03,3.19E+02,2.60E+03,1.52E+03,1.81E+02,3.05E+03,3.
246.98675,6.41E+03,8.85E+03,1.11E+04,1.12E+04,1.10E+04,1.11E+04,6.43E+03,6.85E+03,3.59E+03,4.
246.99781,6.95E+03,3.33E+03,5.75E+03,3.01E+03,4.25E+00,1.60E+03,4.17E+03,2.21E+03,1.49E+03,3.
247.11905,2.33E+03,2.04E+03,2.06E+03,1.78E+03,1.56E+03,1.47E+03,3.08E+03,1.69E+03,2.67E+03,1.
247.93207,1.72E+03,1.31E+03,6.30E+01,1.18E+03,1.43E+03,1.95E+03,2.21E+02,1.56E+03,1.71E+03,7.
248.08021,5.34E+02,2.75E+02,2.65E+01,1.46E+03,1.87E+03,1.89E+03,1.54E+02,1.95E+03,1.19E+03,1.
248.98004,2.49E+03,6.00E+00,2.65E+01,3.38E+02,2.10E+03,4.65E+02,7.20E+01,4.99E+02,2.66E+02,5.
249.13481,1.29E+02,1.30E+03,1.69E+03,8.00E+02,6.40E+02,1.89E+03,1.67E+03,1.91E+03,1.27E+03,1.

249.99712,2.17E+03,3.93E+03,7.00E+01,1.55E+03,7.27E+02,1.95E+03,4.40E+03,4.27E+02,3.75E+02,1.
251.11369,1.86E+03,1.79E+03,4.48E+03,1.48E+03,1.52E+03,1.21E+03,1.10E+03,2.10E+03,1.58E+03,1.
252.05176,3.20E+01,2.90E+02,2.45E+01,1.15E+03,4.83E+03,1.67E+03,5.72E+01,2.19E+03,3.03E+02,7.
252.93366,5.18E+04,4.62E+04,2.94E+04,2.82E+04,6.11E+04,3.18E+04,1.80E+04,3.88E+04,3.82E+04,6.
253.09343,1.63E+03,1.64E+03,1.98E+03,1.71E+03,3.94E+03,1.89E+03,1.67E+03,2.23E+03,1.85E+03,2.
253.10848,1.43E+03,7.45E+01,7.47E+02,1.18E+03,1.60E+03,1.77E+02,9.24E+02,2.36E+03,4.76E+02,1.
253.2178,1.41E+03,2.89E+02,1.16E+03,1.15E+03,2.12E+03,1.15E+03,8.18E+02,1.56E+03,1.39E+03,1.4
253.93207,7.17E+03,2.93E+03,2.90E+03,1.87E+03,5.17E+03,2.10E+03,1.90E+03,2.17E+03,3.49E+03,4.
253.93736,1.98E+03,2.41E+03,3.41E+03,1.68E+03,1.94E+03,1.90E+03,1.57E+03,2.21E+03,2.55E+03,2.
253.94204,1.54E+02,2.26E+03,3.81E+02,8.66E+01,4.66E+02,1.60E+03,1.15E+02,1.74E+02,5.84E+02,5.
254.01565,1.36E+04,1.08E+04,1.50E+04,5.72E+03,2.32E+03,2.99E+03,1.54E+04,9.43E+03,1.14E+04,6.
254.43304,1.27E+03,1.60E+03,2.63E+03,7.16E+02,2.14E+03,1.00E+01,1.76E+03,1.27E+03,1.52E+03,1.
254.56347,2.69E+03,2.07E+03,2.29E+03,1.48E+03,7.68E+01,4.96E+03,2.60E+02,1.53E+02,2.45E+01,2.
254.93046,2.19E+04,2.02E+04,1.77E+04,1.33E+04,2.69E+04,1.64E+04,9.03E+03,2.03E+04,2.03E+04,2.
254.94897,7.73E+02,1.78E+03,1.44E+01,2.15E+03,1.50E+03,2.98E+03,3.49E+01,8.44E+01,1.58E+03,1.
255.08796,1.88E+03,1.44E+03,8.23E+02,2.00E+03,1.62E+03,1.52E+03,1.49E+03,1.96E+03,1.96E+03,2.
255.12425,1.58E+03,1.42E+03,8.23E+02,1.48E+03,2.16E+03,1.41E+03,1.80E+03,1.60E+03,1.03E+03,2.
255.16052,1.35E+01,1.96E+03,8.23E+02,6.82E+01,1.53E+03,1.23E+03,1.07E+01,1.56E+03,1.39E+03,1.
255.19688,1.37E+03,2.07E+03,8.23E+02,1.86E+03,2.23E+03,1.97E+03,1.20E+03,1.97E+03,1.90E+03,2.
255.23316,4.75E+04,3.88E+04,3.44E+04,3.79E+04,5.86E+04,2.57E+04,4.36E+04,5.55E+04,2.67E+04,6.
255.51616,2.49E+03,1.35E+03,1.61E+03,2.03E+03,3.42E+03,1.60E+03,7.64E+02,2.32E+03,1.65E+01,1.
255.93121,2.22E+03,2.19E+03,2.85E+03,1.28E+03,3.04E+03,1.99E+03,1.44E+03,2.56E+03,1.90E+03,2.
255.93863,1.00E+02,1.91E+03,2.89E+02,1.20E+02,2.46E+02,2.26E+03,1.29E+02,1.34E+02,1.41E+03,2.
256.06776,1.22E+04,9.47E+03,1.12E+04,5.48E+03,4.20E+03,6.84E+03,1.01E+04,8.78E+03,8.13E+03,4.
256.23638,5.60E+03,5.74E+03,4.44E+03,5.08E+03,6.61E+03,3.68E+03,4.47E+03,6.30E+03,3.52E+03,6.
256.51437,2.08E+03,1.09E+03,3.14E+03,1.62E+03,1.43E+03,8.44E+02,1.38E+00,1.75E+03,1.51E+03,1.
256.52389,1.47E+03,1.64E+02,2.39E+03,1.56E+03,1.05E+02,9.90E+01,1.38E+00,8.84E+01,4.75E+02,2.
256.92898,1.16E+04,1.31E+04,8.72E+03,7.24E+03,1.44E+04,9.71E+03,5.15E+03,1.35E+04,1.11E+04,1.
257.02642,7.61E+02,6.25E+02,3.16E+02,1.73E+02,1.89E+03,1.92E+03,6.16E+01,1.87E+03,2.17E+03,5.
257.08002,2.23E+03,6.25E+02,3.16E+02,1.10E+03,5.72E+02,4.36E+02,4.02E+02,2.56E+02,1.02E+03,1.
257.10347,1.35E+03,1.73E+03,3.16E+02,1.68E+03,2.74E+03,1.89E+03,1.64E+03,2.33E+03,1.50E+03,2.
257.13964,1.64E+03,2.57E+03,3.16E+02,2.62E+03,3.50E+03,2.75E+03,1.35E+03,2.52E+03,3.04E+03,3.
257.23995,1.80E+02,1.24E+03,1.18E+02,7.95E+02,1.47E+03,4.20E+01,1.21E+03,1.37E+03,1.41E+03,1.
257.43787,3.91E+03,1.59E+03,2.67E+03,2.27E+03,2.93E+03,1.51E+03,1.96E+03,2.67E+03,1.30E+03,2.
257.9235,7.40E+02,6.72E+02,3.14E+03,3.00E+01,3.20E+03,1.86E+03,2.85E+02,5.51E+02,5.08E+02,2.0
257.93298,1.46E+03,1.08E+03,1.57E+03,1.37E+03,3.58E+03,1.41E+03,1.51E+03,1.80E+03,1.34E+03,1.
258.06445,1.59E+03,2.95E+03,7.78E+02,1.10E+03,2.20E+03,2.20E+03,1.62E+03,2.35E+03,1.68E+03,2.
258.09301,1.11E+04,2.74E+04,1.84E+04,1.05E+04,3.29E+03,1.97E+04,1.78E+04,8.27E+03,2.34E+04,1.
258.09913,1.95E+03,1.63E+03,3.02E+03,1.78E+03,3.04E+03,2.16E+03,4.30E+02,3.12E+03,1.00E+03,3.
258.43663,2.05E+03,1.92E+03,4.30E+03,1.25E+03,1.70E+03,1.49E+03,1.17E+03,5.43E+03,2.02E+03,2.
258.59489,2.47E+03,5.56E+03,6.58E+03,2.79E+03,1.79E+03,3.74E+03,4.30E+03,1.83E+03,5.95E+03,2.
258.99537,4.62E+02,1.84E+03,2.49E+02,2.23E+02,1.60E+03,1.97E+03,6.09E+02,1.22E+03,1.45E+03,4.
259.02294,1.96E+04,2.93E+04,3.56E+04,2.01E+04,9.72E+04,2.75E+04,1.01E+04,2.69E+04,1.61E+04,3.
259.08292,1.59E+03,1.24E+03,2.63E+02,1.03E+03,3.69E+01,1.70E+03,1.25E+03,1.32E+03,1.18E+03,1.

259.09575,1.16E+03,1.36E+03,3.52E+03,2.20E+01,3.69E+01,1.53E+03,1.57E+03,4.00E+00,1.36E+03,5.
259.11929,2.22E+03,2.59E+03,1.18E+02,2.34E+03,3.09E+03,2.35E+03,1.76E+03,2.47E+03,2.45E+03,3.
259.15565,2.25E+03,2.13E+03,1.18E+02,1.48E+03,2.19E+03,1.26E+03,1.77E+03,1.77E+03,1.61E+03,1.
259.43592,1.93E+02,1.07E+03,2.23E+03,1.60E+03,5.42E+01,1.92E+03,1.33E+03,3.33E+01,9.70E+01,1.
260.02617,1.49E+03,1.75E+03,3.11E+03,1.33E+03,3.38E+03,2.14E+03,1.39E+03,1.81E+03,1.91E+03,2.
260.08934,1.61E+03,1.79E+03,6.55E+02,1.56E+03,2.27E+03,2.10E+03,1.33E+03,7.43E+01,1.40E+00,2.
260.11458,8.59E+03,1.88E+03,2.58E+04,1.57E+03,2.68E+03,2.06E+03,1.31E+03,3.84E+03,1.40E+00,1.
260.99337,2.12E+03,2.22E+03,1.63E+03,1.39E+03,1.57E+03,1.40E+03,9.28E+02,1.22E+03,1.49E+03,1.
261.00233,1.80E+03,2.88E+03,1.20E+00,2.54E+03,2.64E+02,2.22E+03,1.72E+03,8.73E+01,8.63E+01,1.
261.02018,1.79E+03,1.46E+03,1.20E+00,1.41E+02,5.19E+02,7.82E+01,9.20E+01,1.67E+03,1.59E+03,1.
261.02719,6.48E+02,5.53E+02,1.20E+00,1.41E+02,1.53E+03,1.58E+03,9.20E+01,1.13E+02,4.30E+01,9.
261.07331,2.15E+03,9.00E+01,1.42E+03,1.36E+03,1.73E+03,1.74E+03,1.04E+03,1.06E+03,1.81E+03,1.
261.1171,2.31E+03,1.14E+03,2.94E+03,4.74E+02,8.97E+02,2.08E+03,2.08E+03,4.49E+02,1.75E+03,7.3
261.13484,1.69E+03,5.10E+02,1.91E+03,1.26E+03,1.83E+03,8.54E+02,1.52E+03,2.01E+03,1.44E+03,7.
264.91355,2.12E+02,1.14E+03,1.68E+03,2.17E+02,3.75E+03,5.44E+02,4.19E+02,4.88E+02,1.56E+03,3.
265.09355,1.25E+03,1.60E+03,1.89E+03,4.26E+02,3.73E+02,1.26E+03,7.76E+02,1.01E+03,1.30E+03,1.
265.14854,4.10E+03,2.74E+03,2.53E+03,6.16E+03,5.81E+03,4.58E+03,2.16E+03,5.30E+03,2.96E+03,3.
265.98317,1.21E+03,1.43E+03,1.59E+03,2.39E+03,5.10E+02,1.85E+01,1.66E+03,2.06E+03,1.89E+03,1.
266.15186,4.08E+02,7.50E+02,5.48E+02,1.20E+03,1.59E+03,1.65E+03,6.35E+02,9.90E+01,7.40E+01,5.
266.91062,4.08E+02,5.91E+02,2.25E+03,8.34E+02,4.35E+03,5.33E+02,2.73E+03,9.90E+01,7.40E+01,3.
266.97002,1.92E+03,1.87E+03,2.84E+03,8.34E+02,4.16E+03,4.67E+01,2.37E+03,9.90E+01,1.93E+03,3.
266.97947,2.43E+02,6.58E+01,3.03E+03,8.34E+02,2.72E+03,4.67E+01,7.16E+01,9.90E+01,2.18E+02,1.
267.07298,6.09E+03,4.84E+03,5.71E+03,3.64E+03,1.05E+04,9.90E+03,1.74E+03,2.98E+03,5.13E+03,7.
267.12442,8.37E+02,1.79E+03,4.53E+01,8.18E+02,5.42E+02,1.42E+03,1.34E+03,1.57E+03,1.02E+03,1.
268.03148,3.63E+04,2.64E+04,3.35E+04,1.74E+04,4.35E+03,4.37E+03,4.15E+04,2.43E+04,3.20E+04,1.
268.07581,1.40E+02,9.24E+01,1.66E+02,1.80E+01,1.45E+03,1.75E+03,3.67E+02,7.70E+01,5.84E+02,1.
268.10636,1.24E+03,3.22E+03,2.49E+03,1.42E+03,1.35E+03,1.93E+03,2.11E+03,1.27E+03,1.92E+03,3.
269.03486,2.22E+03,1.96E+03,3.79E+03,1.71E+03,3.98E+02,9.30E+01,3.15E+03,2.74E+03,2.88E+03,2.
269.10361,2.08E+03,2.63E+03,2.07E+02,1.03E+03,1.37E+03,1.56E+03,1.45E+03,1.79E+03,1.93E+03,1.
269.13994,2.09E+03,1.75E+03,2.07E+02,1.89E+03,1.16E+03,1.48E+03,1.53E+02,2.38E+03,8.87E+02,1.
269.17627,8.55E+01,1.62E+03,2.07E+02,1.50E+03,2.35E+03,5.76E+02,1.91E+03,1.97E+03,1.03E+03,1.
269.21263,2.83E+02,1.50E+03,4.17E+02,1.08E+03,1.07E+02,2.28E+03,5.22E+02,8.88E+02,5.60E+02,1.
269.24896,1.02E+03,1.33E+03,2.52E+02,7.18E+02,1.47E+03,1.02E+03,9.11E+02,7.85E+02,1.04E+03,1.
270.94452,3.28E+03,2.78E+03,2.35E+03,3.36E+03,3.48E+03,3.20E+03,1.93E+03,2.37E+03,1.86E+03,2.
270.98697,2.54E+03,2.43E+03,3.92E+03,2.11E+03,1.88E+03,6.65E+01,2.02E+03,2.05E+03,9.04E+02,3.
271.09702,2.14E+03,1.21E+03,1.61E+02,1.50E+03,1.77E+03,1.74E+03,2.28E+03,2.55E+03,2.05E+03,2.
271.11945,1.62E+03,1.36E+03,6.98E+02,1.82E+03,1.54E+03,1.92E+03,1.79E+03,1.88E+03,1.68E+03,2.
271.15556,1.26E+03,3.35E+01,6.98E+02,1.38E+03,1.95E+03,6.47E+01,8.90E+02,1.93E+03,5.29E+02,1.
271.1921,1.88E+02,1.51E+03,6.98E+02,1.24E+03,2.16E+03,1.18E+03,2.72E+02,1.44E+03,8.21E+02,2.0
271.22872,1.14E+03,2.00E+03,2.27E+02,9.57E+02,1.67E+03,3.03E+03,2.32E+02,1.04E+03,8.54E+01,7.
272.02683,1.29E+02,2.31E+03,1.25E+03,1.47E+03,4.89E+02,1.80E+03,4.94E+02,1.50E+03,1.68E+03,3.
272.06284,1.50E+03,2.34E+03,3.42E+03,2.24E+03,7.15E+02,1.43E+03,1.74E+03,1.36E+03,1.60E+03,1.
272.94153,2.03E+03,7.70E+02,1.14E+03,2.09E+03,2.96E+03,1.89E+03,1.36E+03,1.65E+03,1.93E+03,1.
273.01111,4.07E+01,1.45E+03,3.40E+01,3.83E+02,8.43E+02,6.78E+02,1.77E+02,1.29E+03,1.78E+03,1.

273.03905,1.35E+03,2.26E+03,2.58E+03,1.81E+03,5.52E+03,1.82E+03,1.33E+03,2.42E+03,2.13E+03,3.
273.07496,1.19E+02,1.46E+03,1.19E+02,2.09E+03,1.71E+03,1.75E+03,1.49E+03,1.59E+03,5.83E+02,6.
273.09884,1.59E+03,2.60E+03,1.19E+02,1.14E+03,2.08E+03,7.90E+02,1.38E+03,1.87E+03,1.52E+03,1.
273.11695,1.69E+03,1.98E+03,2.38E+03,2.47E+03,8.20E+00,1.33E+03,2.85E+03,8.52E+02,1.86E+03,5.
273.13519,2.16E+03,2.35E+03,1.03E+02,2.69E+03,3.50E+03,2.36E+03,1.73E+03,2.97E+03,2.25E+03,3.
274.94012,1.59E+03,1.25E+03,4.58E+02,1.87E+03,2.78E+03,2.06E+03,1.58E+03,2.05E+03,3.49E+02,2.
275.11402,1.37E+03,1.50E+03,6.11E+02,1.18E+03,1.51E+03,1.54E+03,1.21E+03,1.82E+03,1.37E+03,1.
275.99909,3.47E+03,1.69E+02,2.02E+03,5.38E+02,2.10E+03,8.08E+02,2.23E+03,3.66E+02,2.15E+02,2.
277.01215,1.95E+03,2.64E+03,2.93E+03,3.18E+03,2.71E+03,2.46E+03,1.45E+03,2.44E+03,2.57E+03,1.
277.02366,1.70E+03,1.41E+03,1.82E+02,1.56E+03,2.84E+03,2.02E+03,7.85E+02,1.64E+03,1.40E+03,1.
277.03307,4.45E+03,4.29E+03,6.34E+03,9.73E+03,1.30E+04,6.05E+03,1.12E+04,6.63E+03,5.18E+03,8.
277.04923,2.22E+02,2.39E+03,2.43E+01,6.15E+01,1.40E+00,1.55E+03,7.10E+01,1.85E+03,1.67E+03,1.
277.21766,2.73E+04,4.35E+03,2.05E+04,2.48E+04,5.73E+04,5.13E+03,2.88E+03,1.07E+05,1.33E+04,5.
278.06724,4.17E+01,1.96E+03,2.97E+03,1.70E+03,1.34E+03,1.78E+03,2.58E+03,2.14E+03,1.39E+03,1.
278.22063,5.87E+03,1.71E+03,2.63E+03,4.02E+03,7.23E+03,1.79E+03,8.20E+01,1.27E+04,3.16E+03,1.
279.03851,8.28E+01,6.75E+02,4.40E+01,1.83E+03,2.86E+03,1.57E+03,2.85E+01,3.40E+01,5.81E+02,2.
279.05122,2.26E+05,2.61E+05,4.19E+05,9.34E+04,3.07E+05,1.92E+05,1.40E+05,3.74E+05,2.50E+05,3.
279.10048,5.23E+02,1.51E+03,8.82E+01,1.57E+03,7.92E+02,1.73E+03,1.73E+01,2.06E+03,1.05E+03,5.
279.13474,4.12E+02,8.64E+02,2.84E+03,1.01E+03,1.90E+03,1.20E+02,6.65E+02,8.42E+01,6.60E+02,5.
279.23383,4.52E+02,2.16E+02,2.79E+03,2.50E+03,6.06E+03,2.27E+03,4.72E+02,5.40E+03,2.06E+03,2.
280.05497,1.29E+04,1.54E+04,2.45E+04,7.30E+03,1.69E+04,1.17E+04,9.65E+03,1.90E+04,1.54E+04,1.
280.23677,5.80E+01,6.17E+01,2.70E+03,6.84E+01,2.60E+03,1.23E+02,1.50E+01,1.69E+03,8.10E+01,8.
281.05569,1.52E+03,2.32E+03,2.05E+03,1.04E+03,2.39E+03,8.54E+02,1.09E+03,1.82E+03,1.58E+03,1.
281.06755,2.81E+03,2.32E+03,3.86E+03,1.42E+03,1.55E+03,2.18E+03,6.48E+01,6.01E+03,3.42E+03,3.
281.24881,3.61E+03,1.79E+03,2.66E+03,2.09E+03,2.87E+03,1.94E+03,1.82E+03,3.53E+03,1.74E+03,2.
282.02055,5.52E+03,1.94E+03,6.12E+03,7.65E+02,1.15E+03,1.40E+03,1.76E+03,6.96E+02,1.59E+03,1.
282.07587,2.83E+03,9.20E+01,3.41E+03,1.63E+03,2.61E+03,1.24E+03,1.25E+03,2.60E+03,8.77E+02,2.
282.12143,2.05E+03,9.20E+01,1.34E+03,1.67E+02,1.98E+03,1.51E+03,7.30E+02,8.60E+01,3.84E+02,4.
282.25254,9.00E+01,1.33E+02,1.89E+02,9.08E+02,2.34E+03,1.23E+02,2.77E+02,1.26E+03,7.09E+02,7.
283.02531,2.14E+02,1.19E+02,2.76E+02,1.10E+01,1.18E+03,5.67E+01,3.42E+02,6.60E+01,1.20E+03,1.
283.06761,1.90E+03,2.39E+03,2.21E+03,2.07E+03,2.33E+03,2.52E+03,1.63E+03,1.62E+03,2.09E+03,1.
283.07884,1.69E+03,8.06E+02,2.35E+02,5.16E+02,1.91E+03,9.80E+02,5.44E+02,1.07E+03,5.48E+02,1.
283.11936,1.83E+02,1.29E+03,8.70E+01,1.09E+03,2.21E+03,1.53E+03,1.08E+03,1.45E+03,1.48E+03,1.
283.15554,1.32E+03,2.07E+03,8.70E+01,1.50E+03,1.53E+03,1.78E+03,1.55E+03,1.91E+03,7.73E+01,1.
283.26439,1.37E+04,1.48E+04,1.50E+04,1.49E+04,2.20E+04,1.14E+04,1.53E+04,1.85E+04,1.35E+04,2.
284.07239,2.01E+03,1.32E+03,9.94E+02,1.29E+03,1.79E+03,1.26E+03,7.35E+01,2.07E+03,3.94E+02,1.
284.09904,4.01E+02,1.34E+03,9.94E+02,2.37E+03,6.92E+02,9.33E+02,7.35E+01,1.96E+02,3.97E+02,1.
284.26779,4.01E+02,3.68E+03,4.08E+03,4.02E+03,3.61E+03,3.02E+03,3.36E+03,4.14E+03,2.58E+03,4.
285.06184,3.70E+02,1.35E+02,6.67E-01,1.64E+03,1.78E+03,1.22E+03,3.00E+01,1.20E+03,1.37E+02,3.4
285.0982,8.10E+02,1.11E+03,6.67E-01,1.33E+03,3.01E+03,1.02E+02,3.00E+01,1.54E+03,2.05E+03,1.5
285.13453,1.69E+03,2.67E+03,6.29E+02,1.16E+03,1.84E+03,1.69E+03,1.09E+03,1.84E+03,1.19E+03,1.
285.17085,1.32E+03,3.12E+03,6.29E+02,9.13E+02,1.27E+03,1.38E+03,2.08E+03,1.52E+03,2.00E+03,1.
285.20724,1.68E+03,1.42E+03,6.29E+02,1.66E+03,1.83E+03,1.48E+03,1.77E+03,2.05E+03,1.56E+03,1.
285.27102,1.72E+02,5.00E+01,9.50E+01,1.14E+02,6.30E+01,5.01E+02,1.71E+02,7.09E+02,7.68E+01,1.

286.04173,2.12E+03,2.07E+03,1.80E+01,1.38E+03,1.80E+03,2.58E+02,1.55E+03,1.81E+03,1.69E+03,1.
287.09081,2.02E+03,1.57E+03,2.16E+03,1.16E+03,8.43E+02,2.21E+03,1.55E+03,1.57E+03,3.73E+02,2.
287.11446,1.56E+03,1.41E+03,1.60E+02,1.28E+03,1.33E+03,1.23E+03,1.13E+03,1.55E+03,2.06E+03,1.
287.13194,1.54E+03,1.84E+03,1.60E+02,1.17E+03,1.30E+03,1.77E+03,5.47E+01,1.76E+03,1.14E+03,1.
287.15047,5.52E+02,1.02E+03,1.60E+02,1.57E+03,2.70E+03,1.53E+03,1.57E+03,2.22E+03,1.19E+03,2.
288.91034,2.33E+03,9.20E+02,1.60E+02,1.75E+03,2.31E+03,1.84E+03,1.34E+02,2.10E+03,2.45E+03,2.
289.0332,2.72E+02,2.89E+03,3.29E+03,1.24E+03,5.30E+03,2.24E+03,6.44E+02,1.93E+03,1.89E+03,2.3
289.07007,1.73E+03,3.82E+01,1.26E+02,1.48E+03,2.84E+03,1.01E+03,1.80E+02,1.69E+03,1.36E+03,1.
289.12959,2.72E+03,1.67E+03,9.24E+02,2.85E+03,2.27E+03,1.91E+03,1.53E+03,2.84E+03,2.46E+03,2.
290.90689,1.85E+03,2.37E+03,2.10E+03,1.41E+03,3.12E+03,2.20E+03,1.68E+03,2.42E+03,2.50E+03,3.
291.04899,1.10E+02,2.04E+03,4.97E+03,4.24E+03,5.83E+03,3.97E+03,6.32E+03,4.85E+03,3.98E+03,2.
291.14552,1.76E+03,1.40E+03,3.55E+03,1.60E+03,2.18E+03,2.88E+03,1.68E+03,1.62E+03,1.80E+03,1.
291.18148,1.94E+03,1.29E+03,2.39E+03,1.83E+03,1.87E+03,1.87E+03,9.49E+02,1.31E+03,2.08E+03,1.
291.19658,3.46E+03,1.77E+03,3.08E+03,6.07E+03,1.18E+04,2.92E+03,1.21E+03,1.31E+04,3.32E+03,1.
292.19988,2.74E+02,1.44E+02,5.61E+02,2.06E+03,2.82E+03,2.34E+02,2.02E+02,2.51E+03,1.75E+03,7.
292.90532,2.23E+03,1.44E+02,2.26E+03,1.12E+03,1.46E+03,2.18E+03,1.10E+03,1.83E+03,1.07E+03,1.
293.17618,2.53E+03,1.49E+03,2.99E+03,1.67E+03,2.32E+03,2.17E+03,1.35E+03,1.36E+03,1.30E+03,2.
293.21298,2.87E+03,2.35E+03,2.11E+03,4.45E+03,1.29E+04,4.11E+03,1.67E+03,1.02E+04,3.13E+03,1.
294.07526,1.00E+05,1.94E+04,5.27E+04,7.01E+04,2.26E+05,8.33E+04,1.07E+04,2.06E+05,2.43E+04,3.
294.12124,4.10E+00,2.05E+02,1.81E+03,1.39E+03,4.75E+01,1.97E+03,1.10E+03,1.19E+03,1.62E+03,1.
295.08252,4.91E+05,5.32E+05,5.01E+05,2.60E+05,2.97E+05,6.72E+05,3.04E+05,2.40E+05,7.42E+05,3.
295.22812,8.61E+01,5.04E+02,2.65E+02,1.76E+03,2.62E+03,9.26E+02,9.10E+01,1.56E+03,2.39E+03,1.
296.07259,1.99E+04,5.46E+03,1.18E+04,1.55E+04,3.25E+04,1.93E+04,3.53E+03,3.46E+04,7.37E+03,8.
296.08642,3.87E+04,3.33E+04,4.25E+04,1.34E+04,1.66E+04,4.43E+04,1.79E+04,1.77E+04,5.31E+04,2.
296.09067,9.66E+04,2.02E+04,1.34E+05,1.11E+04,2.56E+04,2.90E+04,1.20E+04,2.55E+04,2.37E+04,1.
296.97096,1.34E+02,1.75E+03,1.82E+03,1.31E+02,3.43E+03,2.09E+03,1.66E+03,2.69E+02,1.69E+03,2.
297.0215,8.84E+02,1.21E+03,1.62E+03,8.55E+02,1.65E+03,1.84E+03,1.27E+03,1.82E+03,4.63E+02,1.5
297.07558,1.98E+03,8.29E+02,1.82E+03,2.03E+03,5.12E+03,3.28E+03,9.25E+01,3.21E+03,1.98E+03,1.
297.08596,1.58E+03,1.92E+03,2.25E+03,1.29E+03,1.80E+03,2.52E+03,1.36E+03,9.91E+02,3.51E+03,1.
297.09441,6.42E+03,1.00E+03,1.11E+04,9.94E+02,3.08E+03,1.64E+03,7.75E+02,3.23E+03,1.52E+03,9.
297.13476,1.99E+03,1.94E+02,6.63E+00,1.10E+03,1.41E+03,9.70E+01,1.20E+03,1.25E+03,1.48E+03,1.
297.15356,9.65E+02,1.42E+03,1.70E+03,1.29E+03,2.21E+03,1.33E+03,1.47E+03,4.27E+01,1.97E+03,2.
297.17104,1.43E+02,4.13E+02,1.28E+02,1.31E+03,2.30E+02,1.55E+03,9.56E+02,1.35E+03,1.98E+02,1.
297.24393,1.68E+03,2.19E+03,3.76E+02,1.24E+03,1.96E+03,1.55E+03,1.48E+02,1.78E+03,1.25E+03,2.
298.01975,8.69E+02,7.02E+02,2.41E+03,4.46E+02,2.37E+03,2.43E+03,7.14E+02,9.70E+02,9.89E+02,1.
298.05061,1.58E+03,1.72E+02,4.80E+02,4.46E+02,2.62E+03,1.54E+03,9.72E+02,1.50E+03,6.30E+01,1.
298.08789,2.06E+04,2.00E+03,2.76E+04,2.56E+03,7.67E+03,1.73E+03,2.30E+03,7.18E+03,1.56E+03,1.
298.11464,1.83E+03,1.28E+03,3.46E+02,1.33E+03,1.27E+02,1.65E+03,2.34E+03,1.32E+03,2.00E+03,6.
298.93892,2.18E+03,1.61E+03,2.23E+03,2.82E+02,1.80E+03,2.31E+03,2.49E+02,1.70E+03,2.02E+03,1.
298.96759,7.39E+02,5.02E+02,3.13E+01,2.82E+02,2.19E+03,1.40E+03,1.40E+03,5.41E+02,4.80E+02,2.
299.07758,9.88E+02,1.36E+02,1.64E+03,2.55E+03,2.06E+03,1.40E+03,1.26E+03,5.17E+02,5.60E+01,1.
299.09124,3.02E+03,2.78E+03,3.31E+03,2.18E+03,1.99E+03,2.50E+03,1.77E+03,2.64E+03,2.28E+03,1.
299.1139,1.16E+03,3.60E+01,2.50E+01,1.38E+03,1.70E+03,1.49E+03,1.46E+03,1.97E+03,1.42E+03,1.0
299.15034,2.02E+03,1.86E+03,3.43E+02,1.73E+03,2.14E+03,1.75E+03,1.93E+03,1.95E+03,1.61E+03,2.

299.18673,1.63E+03,2.08E+03,3.43E+02,1.42E+03,1.64E+03,1.25E+03,1.28E+03,1.45E+03,1.46E+03,1.
299.25906,1.26E+02,1.40E+02,1.66E+02,7.18E+01,1.99E+02,6.70E+02,2.98E+02,8.80E+01,6.71E+02,1.
300.9665,1.85E+03,8.81E+02,2.06E+03,5.78E+02,1.62E+03,2.24E+03,9.30E+01,5.47E+02,7.93E+02,2.7
301.12947,2.17E+03,1.44E+03,1.90E+02,1.69E+03,1.19E+03,2.04E+02,1.54E+03,1.58E+03,1.77E+03,1.
301.16558,8.93E+02,3.18E+03,1.90E+02,1.65E+03,2.71E+03,1.13E+03,1.65E+03,2.12E+03,1.87E+03,1.
301.2022,1.73E+01,6.52E+02,1.90E+02,1.88E+03,8.35E+02,7.75E+01,4.48E+02,1.49E+03,1.38E+03,1.5
302.06589,1.94E+03,1.56E+03,2.23E+03,1.57E+03,2.78E+03,1.71E+03,1.09E+03,1.41E+03,1.28E+03,2.
303.07257,1.79E+03,2.04E+03,1.70E+03,1.16E+03,1.94E+03,1.96E+03,1.15E+03,1.48E+03,1.85E+03,1.
303.1088,1.84E+03,1.67E+03,1.61E+03,9.63E+02,9.86E+02,1.24E+02,1.35E+03,1.68E+03,5.18E+01,2.0
303.14521,9.46E+02,1.46E+03,2.25E+03,1.15E+03,2.40E+03,1.82E+03,1.85E+03,1.80E+03,1.31E+03,2.
304.10421,2.06E+03,1.63E+03,1.91E+03,1.50E+03,2.45E+03,2.08E+03,1.52E+03,2.90E+03,1.45E+03,7.
305.01859,1.27E+02,3.54E+02,2.20E+00,1.55E+03,1.30E+03,1.23E+03,5.50E+01,4.27E+02,1.39E+03,6.
305.06403,1.51E+03,7.09E+02,2.20E+00,3.06E+02,1.86E+03,2.15E+03,5.50E+01,1.05E+03,1.81E+03,1.
305.16083,2.15E+02,3.97E+02,2.60E+03,1.18E+03,5.01E+02,1.07E+03,1.44E+03,1.09E+03,1.46E+03,1.
306.05832,2.06E+03,2.77E+03,2.15E+03,1.60E+03,3.14E+03,1.35E+03,1.65E+03,1.54E+03,1.48E+03,1.
306.11952,2.96E+03,1.86E+03,3.77E+03,3.43E+03,2.33E+03,1.53E+03,2.15E+03,3.85E+03,2.61E+03,4.
306.88431,2.40E+02,2.36E+03,2.08E+03,1.38E+03,2.41E+02,3.16E+03,1.29E+03,6.62E+02,1.94E+03,4.
307.19182,2.04E+03,5.25E+02,7.91E+02,1.61E+03,6.71E+03,2.21E+02,8.70E+01,4.33E+03,1.52E+03,1.
308.89984,3.70E+03,2.10E+03,2.06E+03,2.14E+03,3.84E+02,1.74E+03,1.28E+03,1.38E+03,1.72E+03,1.
309.04639,3.42E+03,3.54E+03,3.05E+03,2.77E+03,6.40E+02,1.73E+03,4.49E+03,2.32E+03,1.96E+03,1.
309.06094,5.12E+03,7.74E+03,1.07E+04,4.63E+03,6.40E+02,4.23E+03,1.49E+04,5.27E+03,7.83E+03,3.
309.06268,6.15E+03,8.34E+03,1.04E+04,4.05E+03,6.40E+02,4.91E+03,1.24E+04,5.74E+03,7.66E+03,4.
309.09377,3.06E+03,1.71E+03,6.15E+03,2.07E+03,2.32E+03,2.04E+03,1.63E+03,1.57E+03,2.16E+03,1.
309.17406,1.55E+03,1.23E+03,1.03E+02,2.11E+03,2.45E+03,1.60E+03,1.95E+02,1.44E+03,1.34E+03,2.
309.20687,6.41E+03,2.49E+03,3.01E+03,1.16E+04,3.86E+04,3.77E+03,2.63E+03,2.44E+04,7.56E+03,2.
310.06479,1.81E+03,1.54E+03,1.90E+03,1.96E+03,2.87E+03,9.47E+02,1.68E+03,2.19E+03,1.15E+03,2.
310.17798,4.74E+02,2.85E+02,4.93E+02,2.05E+02,1.23E+02,6.08E+02,1.61E+02,1.48E+03,2.56E+02,2.
310.21037,1.79E+03,1.20E+03,4.93E+02,1.99E+03,6.28E+03,1.60E+03,1.61E+02,4.11E+03,1.38E+03,2.
310.89732,2.20E+03,1.41E+03,4.93E+02,1.78E+00,9.79E+01,2.96E+02,1.14E+03,8.47E+02,1.74E+03,2.
310.9666,1.80E+03,2.18E+03,1.90E+02,1.65E+03,1.34E+03,2.96E+02,2.22E+03,2.52E+03,1.45E+03,1.9
311.07701,3.28E+03,5.95E+02,4.72E+03,1.45E+03,2.50E+03,2.06E+03,2.12E+03,6.05E+03,4.46E+03,3.
311.10997,2.37E+03,2.34E+03,2.18E+03,1.11E+03,1.53E+04,2.13E+03,1.49E+03,2.05E+03,1.91E+03,5.
311.15043,6.95E+02,9.64E+01,4.19E+02,8.91E+02,1.08E+01,8.36E+02,9.87E+02,3.01E+02,1.67E+02,1.
311.16886,3.34E+03,2.92E+03,3.92E+03,3.29E+03,8.69E+03,3.19E+03,1.69E+03,3.80E+03,2.32E+03,5.
311.22317,1.21E+03,1.51E+03,9.00E+01,1.19E+03,6.26E+03,1.61E+03,5.90E+01,3.03E+03,1.72E+03,1.
311.97035,1.71E+03,1.15E+03,9.08E+02,1.20E+01,1.86E+03,1.14E+03,7.36E+02,7.65E+02,1.12E+03,2.
312.08572,1.10E+03,4.04E+02,1.31E+03,1.10E+02,1.99E+03,1.02E+02,2.09E+02,1.33E+03,1.66E+03,6.
312.15062,9.71E+02,2.29E+03,2.48E+03,1.37E+02,1.44E+03,1.06E+03,7.97E+02,2.70E+02,9.60E+02,1.
312.95483,2.89E+02,1.50E+03,1.50E+03,1.64E+03,1.76E+03,1.27E+03,6.40E+01,1.66E+03,1.43E+03,1.
313.11441,7.77E+02,8.75E+02,3.55E+01,9.30E+02,2.39E+03,1.33E+03,6.62E+02,1.09E+03,2.45E+03,3.
313.12941,1.97E+03,1.38E+03,3.55E+01,1.24E+03,7.54E+02,2.17E+03,1.56E+03,1.47E+03,1.17E+03,3.
313.16625,1.85E+03,1.26E+02,3.55E+01,1.40E+03,1.58E+02,6.53E+02,1.35E+03,1.36E+03,1.13E+03,1.
313.23893,2.48E+02,8.64E+02,9.00E+01,1.93E+03,1.38E+03,1.65E+03,1.26E+03,1.40E+03,1.64E+03,1.
313.941,2.48E+02,1.84E+03,1.60E+03,1.37E+03,5.98E+01,2.40E+02,1.61E+03,1.67E+03,1.57E+03,9.70

314.10993,2.48E+02,1.50E+03,2.74E+03,3.14E+02,2.10E+02,9.34E+02,1.93E+03,7.13E+02,1.39E+03,1.
314.97982,2.49E+03,2.33E+03,2.47E+03,2.43E+03,4.23E+02,1.89E+03,2.21E+03,2.07E+03,1.86E+03,1.
315.08504,1.65E+03,1.35E+03,2.22E+03,1.20E+03,1.45E+03,2.05E+03,1.59E+03,2.93E+03,3.17E+03,1.
315.10852,1.79E+03,5.75E+01,1.12E+02,1.72E+01,1.52E+02,2.39E+03,7.65E+01,5.04E+01,1.29E+03,1.
315.14499,1.63E+03,1.55E+03,5.98E+02,4.80E+02,2.65E+03,1.59E+03,1.56E+03,1.98E+03,1.27E+03,2.
315.18153,1.72E+03,3.00E+03,5.98E+02,1.25E+03,1.19E+03,1.63E+03,1.54E+03,1.43E+03,1.88E+02,1.
316.06989,1.91E+02,2.16E+03,9.62E+02,1.36E+03,3.22E+03,1.96E+03,1.39E+03,1.22E+03,1.01E+03,2.
316.08886,3.59E+03,1.29E+02,2.06E+03,2.43E+02,1.23E+03,1.48E+03,2.14E+03,1.51E+03,1.92E+03,1.
317.0068,4.55E+03,4.54E+03,7.33E+03,2.96E+03,2.36E+03,2.36E+03,4.70E+03,3.54E+03,2.88E+03,3.0
317.05431,5.17E+03,1.21E+04,9.21E+03,4.45E+03,1.49E+04,2.70E+03,1.02E+04,6.53E+03,9.29E+03,1.
317.12429,9.03E+02,3.60E+01,5.30E+00,1.49E+03,2.09E+03,1.38E+03,1.07E+03,1.36E+03,1.48E+03,1.
317.1609,1.16E+03,1.25E+03,7.83E+02,1.27E+03,1.72E+03,1.65E+03,1.11E+03,1.51E+03,1.35E+03,1.2
318.05817,1.71E+03,1.44E+03,1.42E+03,1.21E+03,1.90E+03,3.60E+02,1.32E+03,1.09E+03,1.91E+03,1.
318.07681,6.43E+02,1.15E+03,2.63E+02,8.83E+02,1.51E+03,3.60E+02,3.80E+01,1.50E+03,3.50E+01,1.
318.11968,1.72E+03,4.60E+02,2.13E+03,2.03E+03,2.28E+03,1.33E+03,3.80E+01,2.10E+03,1.46E+03,1.
319.08396,1.24E+04,1.56E+04,1.10E+04,9.93E+03,2.33E+04,1.29E+04,8.80E+03,9.11E+03,1.00E+04,1.
319.09427,1.52E+02,3.15E+03,2.32E+03,1.66E+03,3.53E+03,2.89E+03,1.05E+03,2.28E+03,1.13E+03,3.
319.24081,1.48E+03,3.27E+00,1.78E+02,9.74E+02,2.31E+03,3.50E+01,1.48E+03,1.89E+03,1.64E+03,1.
319.58548,5.76E+03,2.80E+03,5.03E+03,3.84E+03,5.68E+03,3.40E+03,2.00E+03,3.05E+03,6.36E+03,4.
320.082,2.31E+03,1.85E+03,2.13E+03,1.92E+03,2.42E+03,2.07E+03,9.52E+02,5.13E+02,2.09E+03,1.80
320.09169,4.39E+03,1.01E+03,2.14E+04,3.99E+03,7.67E+00,1.74E+03,6.20E+03,1.67E+03,2.85E+03,1.
320.13492,1.09E+03,1.36E+02,2.08E+03,1.54E+03,1.73E+03,5.96E+02,2.60E+02,5.13E+02,1.59E+02,8.
320.58383,7.06E+02,1.68E+03,1.50E+03,4.21E+02,1.43E+03,1.81E+03,8.43E+01,5.13E+02,1.95E+03,2.
321.08203,3.76E+03,5.67E+03,4.34E+03,2.53E+03,1.66E+03,7.63E+03,2.73E+03,2.73E+03,5.63E+03,5.
321.09372,8.22E+04,1.59E+04,1.22E+05,2.34E+04,2.61E+04,2.19E+04,9.97E+03,5.62E+04,1.33E+04,9.
322.08654,8.15E+02,1.67E+03,2.00E+03,7.06E+02,3.72E+02,2.02E+03,6.37E+02,2.46E+02,1.30E+03,1.
322.09643,5.54E+03,2.00E+03,8.62E+03,3.83E+03,2.98E+03,2.68E+03,1.93E+03,5.71E+03,2.80E+02,1.
322.0983,1.79E+02,1.96E+03,8.59E+03,3.27E+03,2.99E+03,3.75E+03,1.93E+03,5.07E+03,2.80E+02,1.0
322.11468,2.55E+03,2.58E+02,1.91E+03,1.34E+03,2.36E+03,1.69E+03,1.79E+03,1.77E+03,1.28E+03,1.
323.09818,2.67E+02,1.79E+03,7.52E+02,1.13E+03,1.16E+03,1.56E+03,3.61E+02,1.93E+02,1.18E+03,2.
323.1097,1.68E+05,5.90E+03,5.38E+05,8.01E+03,1.19E+04,3.20E+03,6.90E+03,2.00E+04,2.81E+03,3.5
323.18678,6.16E+02,1.64E+03,1.71E+02,1.34E+03,3.15E+03,1.63E+03,8.48E+02,2.08E+03,1.10E+03,1.
324.02126,2.40E+03,1.35E+03,2.37E+03,1.84E+03,1.59E+03,3.36E+02,1.57E+03,1.33E+03,8.28E+01,7.
324.085,1.87E+03,2.80E+03,6.03E+02,1.91E+03,4.00E+03,4.48E+03,1.51E+03,2.82E+03,2.99E+03,4.22
324.11321,1.00E+04,2.25E+03,2.87E+04,2.17E+01,1.59E+03,1.82E+03,2.24E+03,2.58E+03,8.42E+01,8.
324.58709,1.77E+03,2.20E+03,8.98E+02,1.96E+03,1.59E+03,2.44E+03,2.10E+02,1.33E+03,1.65E+03,1.
325.09308,1.21E+05,1.06E+05,2.30E+05,6.42E+04,6.27E+04,1.41E+05,1.13E+05,9.35E+04,2.34E+05,5.
325.12541,1.36E+02,1.57E+03,6.90E+01,1.75E+02,2.89E+03,9.03E+02,4.28E+01,1.35E+03,1.16E+02,2.
325.18435,3.44E+03,3.27E+03,2.05E+03,3.22E+03,7.77E+03,3.07E+03,1.86E+03,3.55E+03,2.37E+03,5.
325.202,2.36E+03,1.43E+03,1.72E+03,4.16E+03,1.06E+04,2.61E+03,1.56E+03,9.87E+03,3.73E+03,1.37
325.59475,3.58E+02,1.69E+03,3.04E+03,1.47E+03,6.67E+02,1.65E+03,1.48E+03,1.72E+03,1.05E+03,6.
326.0959,1.21E+04,1.02E+04,1.84E+04,6.96E+03,7.74E+03,1.11E+04,1.08E+04,9.36E+03,1.84E+04,6.5
326.10957,1.48E+03,2.16E+03,1.82E+03,1.84E+03,3.74E+03,2.94E+03,2.65E+03,2.42E+03,2.20E+03,2.
326.18774,1.21E+03,1.55E+03,5.56E+01,1.45E+03,2.28E+03,1.29E+03,1.07E+03,1.11E+03,7.59E+01,1.

326.86616,1.89E+03,2.03E+02,5.56E+01,2.20E+03,2.34E+03,2.63E+03,2.28E+03,5.04E+02,1.99E+03,1.
326.98133,4.23E+02,6.39E+02,6.19E+02,1.26E+02,2.51E+03,2.59E+03,3.47E+02,5.73E+02,5.12E+02,2.
327.0982,1.69E+03,1.25E+03,1.55E+03,9.92E+02,7.82E+02,1.14E+03,1.88E+03,1.26E+03,1.30E+03,1.2
327.14505,7.07E+02,1.20E+03,5.29E+02,1.47E+03,1.05E+03,4.65E+01,7.29E+02,2.11E+03,6.81E+02,1.
327.18144,2.01E+02,1.56E+03,5.29E+02,1.07E+03,1.88E+03,1.44E+03,1.59E+02,1.67E+03,1.72E+03,2.
327.21738,1.97E+03,1.63E+03,2.17E+03,2.50E+03,9.10E+03,1.90E+03,1.65E+03,4.10E+03,1.98E+02,2.
327.25418,1.05E+02,1.03E+03,5.30E+02,1.25E+03,1.94E+03,1.79E+03,1.49E+03,1.94E+03,1.55E+03,1.
328.04564,2.98E+02,1.03E+02,5.30E+02,1.54E+03,2.23E+03,1.02E+03,1.87E+03,7.87E+01,2.01E+03,1.
328.22111,9.80E+02,1.03E+02,7.60E+02,1.97E+03,2.09E+03,4.42E+02,1.40E+03,6.69E+02,8.33E+02,1.
328.863,1.99E+03,1.32E+03,7.60E+02,1.35E+03,1.54E+03,1.31E+03,1.21E+02,2.69E+02,8.33E+02,2.22
328.99441,1.55E+02,2.28E+03,2.79E+03,1.38E+03,1.48E+03,1.92E+03,4.85E+03,6.54E+01,2.61E+03,1.
329.08603,1.55E+02,1.42E+03,3.69E+03,1.04E+03,1.51E+03,1.54E+03,1.39E+03,1.35E+03,1.26E+03,1.
329.12425,9.71E+02,1.38E+03,9.55E+02,8.10E+02,1.74E+03,1.45E+03,7.45E+01,2.23E+03,1.59E+03,8.
329.16044,1.66E+03,1.65E+03,1.65E+03,1.47E+03,6.53E+02,1.74E+03,1.57E+03,1.92E+03,1.55E+03,2.
329.23339,2.32E+02,1.34E+03,6.22E+02,2.25E+03,2.06E+03,2.57E+02,3.23E+02,2.07E+03,1.18E+02,1.
331.06792,1.57E+02,3.21E+03,9.71E+02,3.88E+02,7.83E+02,6.34E+02,5.79E+02,2.12E+02,3.50E+01,1.
331.14003,1.57E+02,2.24E+03,9.71E+02,1.40E+03,1.73E+03,2.09E+03,1.35E+03,1.67E+03,1.78E+03,1.
331.17637,1.91E+03,1.57E+03,1.41E+03,1.40E+03,2.51E+03,1.61E+03,1.99E+03,1.87E+03,2.05E+03,1.
333.05883,1.31E+04,7.51E+03,2.13E+04,1.35E+04,3.23E+04,3.85E+03,1.03E+04,1.51E+04,8.84E+03,1.
333.06081,1.20E+04,7.23E+03,2.13E+04,1.26E+04,3.21E+04,2.70E+00,1.03E+04,1.28E+04,8.94E+03,1.
333.10896,6.84E+01,2.94E+03,1.53E+03,1.71E+03,1.80E+03,2.02E+03,1.40E+03,1.01E+03,1.28E+03,2.
333.15571,2.00E+03,2.99E+03,3.75E+03,1.56E+03,2.58E+03,2.68E+03,1.87E+03,2.49E+03,2.79E+03,2.
333.19238,2.09E+03,2.23E+01,3.19E+03,2.11E+03,1.76E+03,2.08E+03,3.56E+03,3.47E+03,5.72E+03,2.
333.65773,2.75E+01,6.01E+02,4.13E+03,1.89E+01,2.80E+02,5.82E+02,1.46E+01,5.79E+02,9.52E+02,1.
334.06305,1.04E+03,1.79E+03,1.54E+03,1.52E+03,3.17E+03,5.14E+02,2.09E+02,2.08E+03,1.63E+03,1.
334.11432,2.61E+03,1.68E+03,3.46E+01,1.56E+03,3.50E+01,1.87E+03,2.08E+03,1.74E+03,6.25E+01,2.
335.06326,7.38E+02,3.06E+02,1.27E+03,1.62E+03,1.67E+03,8.07E+02,1.56E+03,1.75E+03,1.87E+03,1.
336.13034,1.74E+03,1.92E+03,2.74E+02,1.50E+03,1.30E+02,2.67E+03,1.28E+03,2.88E+03,2.19E+03,6.
336.8945,1.41E+02,2.17E+03,1.82E+02,5.30E+01,5.67E+02,2.22E+03,1.12E+02,2.27E+03,9.65E+02,1.4
336.94629,1.66E+03,5.41E+02,1.82E+02,1.91E+03,1.70E+03,1.73E+03,1.12E+02,2.06E+03,1.51E+03,1.
337.07792,1.76E+03,1.57E+03,1.82E+02,1.10E+03,1.16E+03,2.07E+03,3.06E+02,8.32E+02,1.57E+03,1.
337.20548,2.19E+02,1.62E+02,6.43E+02,1.80E+03,1.38E+03,4.44E+02,1.86E+02,1.90E+02,2.34E+02,9.
338.94318,2.79E+03,1.49E+03,6.02E+02,1.85E+03,1.52E+03,1.86E+03,3.40E+01,1.65E+03,1.81E+03,4.
339.10428,2.96E+03,1.68E+03,3.78E+03,1.68E+03,1.85E+03,2.05E+03,7.88E+02,1.25E+03,1.44E+03,1.
339.19972,2.21E+03,1.47E+03,2.73E+03,1.42E+03,3.46E+03,2.21E+03,2.57E+02,1.97E+03,1.77E+03,3.
339.29049,1.93E+03,3.13E+03,7.48E+01,1.06E+03,2.67E+03,1.06E+03,9.04E+02,1.43E+03,1.53E+03,1.
340.00843,6.76E+02,1.32E+03,1.84E+03,1.83E+03,2.63E+03,2.15E+03,1.62E+03,4.36E+01,1.05E+03,2.
340.96083,1.24E+03,6.83E+02,3.69E+02,5.37E+02,1.02E+02,3.03E+01,9.95E+01,3.46E+02,6.41E+02,5.
341.08767,2.00E+03,1.35E+03,2.18E+03,1.43E+03,2.30E+03,1.16E+03,1.06E+03,2.40E+03,1.41E+03,3.
341.10779,1.20E+04,4.22E+04,3.30E+04,1.98E+04,2.08E+04,3.58E+04,1.97E+04,2.75E+04,3.18E+04,2.
341.10979,9.68E+03,4.22E+04,3.61E+04,1.87E+04,1.74E+04,3.29E+04,2.16E+04,3.01E+04,3.22E+04,2.
341.12354,5.92E+01,2.32E+01,3.41E+01,1.07E+03,3.68E+01,7.38E+01,8.93E+01,1.88E+03,3.50E+00,1.
341.16064,1.34E+03,2.38E+03,2.04E+03,1.29E+03,1.25E+03,1.23E+03,5.91E+02,1.33E+03,1.79E+03,1.
341.19665,1.67E+03,2.89E+03,2.40E+03,1.95E+03,5.08E+03,1.67E+03,1.21E+03,3.51E+03,2.02E+03,1.

341.23341,1.36E+03,1.31E+03,8.01E+02,1.21E+03,2.04E+03,1.55E+03,2.86E+02,2.30E+03,2.22E+03,2.
342.10281,2.43E+03,8.84E+02,5.28E+03,1.48E+03,1.08E+02,1.30E+03,1.95E+03,1.88E+03,4.21E+03,1.
342.11342,2.40E+03,3.82E+03,2.97E+03,2.34E+03,3.09E+03,2.00E+02,1.99E+03,3.66E+03,3.88E+03,2.
342.9576,6.52E+02,6.43E+02,8.86E+01,4.45E+02,2.57E+01,5.43E+02,1.80E+01,9.04E+02,5.32E+02,6.0
342.96521,6.52E+02,6.87E+02,8.86E+01,9.66E+02,2.06E+03,1.68E+03,1.80E+01,1.81E+03,5.32E+02,1.
343.11403,9.27E+02,1.19E+03,8.86E+01,9.76E+02,1.06E+03,2.47E+03,7.54E+02,1.09E+03,4.72E+02,4.
343.17682,8.87E+02,1.77E+03,7.53E+02,1.64E+03,1.72E+02,1.51E+03,1.32E+03,1.36E+03,9.25E+02,1.
343.21318,1.82E+03,1.66E+03,7.53E+02,1.77E+03,2.96E+03,1.10E+03,1.30E+03,1.96E+03,1.43E+03,8.
344.14806,1.36E+03,3.16E+03,1.84E+03,1.52E+03,9.79E+02,2.19E+03,1.27E+03,2.72E+03,1.61E+03,1.
345.04272,9.15E+02,6.60E+01,5.14E+03,3.16E+02,2.98E+03,1.05E+02,2.71E+02,5.18E+02,2.13E+03,2.
345.15548,1.78E+03,2.51E+03,2.09E+03,2.07E+03,1.67E+03,2.52E+03,1.24E+03,2.34E+03,2.08E+03,2.
346.11515,1.71E+03,1.63E+02,4.66E+02,1.64E+03,1.15E+03,2.60E+02,1.33E+03,2.22E+02,1.86E+03,8.
347.17153,1.62E+03,1.56E+03,1.62E+03,1.79E+03,2.07E+03,2.07E+03,2.05E+03,1.35E+03,2.00E+03,1.
347.20755,6.68E+02,1.76E+03,1.72E+02,1.09E+03,2.99E+03,1.63E+03,1.69E+03,2.30E+01,1.64E+03,1.
348.1302,2.15E+03,1.85E+03,2.17E+03,1.98E+03,2.18E+03,1.97E+03,1.62E+03,1.72E+03,1.60E+03,2.1
350.04092,1.78E+03,1.82E+03,2.87E+02,1.39E+03,1.52E+03,1.55E+03,8.06E+02,2.32E+02,9.90E+02,1.
350.10899,2.31E+03,1.28E+03,2.87E+02,2.43E+03,1.26E+02,9.50E+01,1.32E+03,1.74E+03,1.55E+03,1.
350.14534,2.06E+03,1.75E+03,1.94E+03,1.30E+03,1.23E+03,1.28E+03,2.40E+03,1.69E+03,1.74E+03,1.
350.91022,1.60E+03,1.79E+03,2.50E+03,2.03E+03,2.19E+03,1.89E+03,1.40E+03,1.97E+03,1.95E+03,1.
351.03907,2.14E+03,2.01E+03,6.25E+02,2.00E+03,1.04E+03,1.08E+03,1.23E+03,1.68E+01,4.66E+02,1.
351.08745,2.20E+03,1.61E+03,6.25E+02,1.83E+03,3.93E+02,2.45E+03,1.71E+03,1.68E+01,1.97E+03,1.
351.11998,2.82E+02,1.18E+03,1.16E+02,7.48E+02,3.95E+02,7.08E+02,9.81E+02,1.74E+03,2.69E+02,5.
351.13053,1.60E+03,1.69E+03,1.59E+03,2.23E+03,1.92E+03,1.23E+02,1.82E+03,1.60E+03,2.13E+03,5.
352.1247,2.91E+03,2.07E+03,1.43E+02,1.13E+03,1.51E+03,1.35E+03,1.98E+03,2.40E+03,2.41E+03,1.6
353.07263,1.27E+03,4.18E+02,7.70E+02,1.12E+03,2.13E+03,9.56E+02,2.09E+02,2.10E+03,1.33E+03,8.
353.20043,1.05E+02,8.44E+02,1.35E+02,1.22E+03,8.17E+02,1.31E+03,4.53E+02,1.33E+03,2.67E+03,4.
355.08793,4.49E+02,3.15E+03,2.73E+03,2.25E+03,2.09E+03,3.08E+03,4.12E+03,1.94E+03,2.55E+03,1.
355.10375,2.93E+03,1.94E+03,3.59E+03,2.51E+03,1.67E+03,1.59E+03,1.65E+03,3.97E+03,3.32E+03,1.
355.17672,8.56E+00,1.18E+02,1.23E+03,1.68E+03,1.38E+03,5.15E+02,1.38E+03,4.35E+02,1.73E+03,1.
356.06492,1.41E+03,1.61E+03,1.82E+03,3.13E+02,2.50E+03,1.30E+03,4.89E+02,1.49E+03,1.13E+02,2.
356.07521,3.72E+03,3.21E+02,8.65E+03,6.29E+03,1.01E+04,4.11E+03,2.80E+03,7.22E+03,2.24E+03,3.
356.07725,5.63E+03,3.21E+02,7.74E+03,6.97E+03,1.14E+04,4.82E+03,2.45E+03,7.22E+03,2.59E+03,2.
357.07875,2.08E+02,6.60E+02,2.41E+03,1.04E+03,3.26E+03,8.20E+02,3.23E+02,1.16E+03,1.48E+02,8.
357.15576,2.08E+02,1.93E+03,7.03E+02,1.36E+03,1.94E+03,1.52E+03,6.74E+02,1.52E+03,1.57E+03,1.
357.19199,1.88E+03,1.20E+03,7.03E+02,1.26E+03,2.54E+03,1.59E+03,3.08E+03,2.40E+03,1.30E+03,1.
357.22833,1.45E+03,8.88E+01,7.03E+02,1.20E+01,1.41E+03,1.51E+02,4.86E+02,1.60E+03,3.08E+01,1.
358.07878,1.94E+03,2.45E+03,2.71E+03,2.97E+03,1.02E+02,2.85E+03,1.95E+03,2.32E+03,3.54E+03,1.
358.09218,4.38E+03,3.47E+01,1.30E+04,1.28E+03,4.15E+03,6.99E+02,2.32E+03,1.94E+03,9.66E+01,1.
358.99625,6.04E+02,2.45E+03,6.94E+02,4.89E+02,1.02E+02,2.41E+03,6.75E+02,2.09E+03,1.70E+03,6.
359.11951,6.04E+02,1.70E+03,1.78E+03,1.20E+03,8.07E+02,1.46E+03,7.40E+02,2.37E+03,1.00E+03,1.
359.17087,1.37E+03,2.45E+03,2.69E+03,1.56E+03,2.25E+03,2.05E+03,1.54E+03,1.97E+03,1.72E+03,1.
359.2074,2.43E+02,6.83E+02,3.59E+02,4.97E+02,2.36E+03,1.77E+03,1.95E+02,3.60E+01,2.14E+03,1.7
361.18695,1.82E+03,1.71E+03,1.76E+03,1.73E+03,4.46E+02,2.02E+03,1.83E+03,1.25E+03,1.44E+03,1.
362.10954,2.09E+03,2.40E+03,3.79E+03,1.62E+03,3.11E+03,2.26E+03,1.60E+03,2.45E+03,1.41E+03,1.

362.14543,2.78E+03,2.06E+03,6.96E+02,2.50E+01,2.92E+01,4.32E+01,2.69E+03,2.17E+03,2.77E+02,1.
363.04988,2.40E+03,1.10E+03,6.18E+02,1.49E+03,5.32E+02,1.26E+03,1.20E+02,2.65E+03,1.61E+03,1.
363.12961,1.18E+03,3.23E+02,2.63E+03,1.67E+03,2.27E+03,1.68E+03,7.82E+02,3.05E+03,1.72E+03,3.
363.63155,1.80E+03,3.23E+02,5.46E+02,1.63E+03,1.81E+03,1.49E+03,2.13E+02,1.13E+03,4.65E+02,1.
364.0491,2.29E+03,1.18E+03,4.26E+03,9.78E+02,6.73E+03,2.46E+03,1.32E+03,2.07E+03,9.88E+02,4.5
364.05145,2.20E+03,1.43E+03,3.08E+03,1.22E+03,7.27E+03,1.97E+03,1.30E+03,1.16E+03,9.88E+02,3.
364.12504,2.74E+03,1.78E+03,8.39E+03,1.57E+03,1.32E+03,2.18E+03,2.58E+02,2.07E+03,2.08E+03,6.
365.04931,2.13E+02,4.11E+02,4.33E+02,3.75E+02,1.03E+03,3.66E+03,3.10E+02,1.16E+03,1.78E+03,1.
365.13696,2.13E+02,1.93E+03,4.33E+02,8.61E+02,2.08E+03,2.17E+03,1.72E+03,2.48E+03,1.69E+03,1.
365.24589,3.40E+03,3.19E+03,3.37E+03,4.48E+03,4.06E+03,2.74E+03,1.98E+03,4.92E+03,4.35E+03,5.
365.24818,2.43E+03,2.35E+02,2.18E+03,1.19E+02,8.08E+01,3.98E+03,1.69E+03,5.97E+03,4.40E+03,6.
366.04571,5.76E+02,2.09E+03,2.28E+03,6.82E+02,4.54E+03,1.54E+03,1.62E+03,1.24E+03,1.75E+03,2.
366.14074,2.48E+03,1.69E+03,1.53E+01,2.17E+02,2.47E+03,3.09E+02,7.47E+02,2.13E+03,1.37E+03,1.
366.25003,1.75E+03,1.75E+03,1.85E+03,1.53E+03,1.97E+03,2.15E+03,3.68E+02,2.18E+03,2.33E+03,1.
366.93863,1.92E+02,2.17E+02,2.26E+03,7.46E+02,1.98E+02,5.60E+02,7.15E+02,1.50E+02,5.00E-01,4.
367.24298,2.20E+03,2.38E+03,3.54E+03,1.46E+03,1.49E+03,9.71E+02,4.17E+02,2.13E+03,2.19E+03,2.
367.35816,8.50E+01,1.05E+02,2.09E+02,2.03E+02,2.36E+02,8.87E+02,2.05E+02,1.89E+02,1.30E+02,8.
368.04522,4.87E+02,1.63E+03,1.53E+03,1.24E+03,2.31E+03,1.96E+02,9.00E+00,1.98E+03,4.48E+02,1.
368.9359,6.85E+01,6.54E+02,4.30E+02,4.47E+02,4.00E+01,1.70E+01,2.41E+02,6.66E+02,4.07E+02,3.3
368.93831,6.85E+01,6.54E+02,4.30E+02,4.47E+02,4.00E+01,1.70E+01,2.41E+02,6.66E+02,4.07E+02,3.
369.15574,5.44E+02,1.38E+03,9.55E+02,4.39E+02,6.40E+02,8.92E+02,1.59E+03,4.87E+02,1.93E+03,1.
370.93327,3.78E+02,2.80E+02,3.14E+02,3.25E+02,1.56E+02,7.30E+01,2.75E+02,3.06E+02,1.44E+02,2.
371.17069,7.62E+02,2.04E+03,8.81E+02,1.45E+03,1.81E+03,1.44E+03,5.48E+02,2.26E+03,1.32E+03,1.
371.20681,1.54E+02,2.78E+03,8.81E+02,1.93E+03,1.43E+03,2.48E+03,5.96E+02,1.40E+03,2.51E+02,1.
372.20257,4.29E+03,2.20E+02,3.85E+02,1.89E+03,2.11E+03,6.48E+02,2.52E+02,2.35E+03,1.91E+02,4.
373.13523,1.85E+03,1.43E+03,1.72E+03,1.34E+03,2.75E+02,7.83E+02,1.40E+03,2.13E+03,9.16E+02,1.
373.1869,2.65E+01,1.72E+03,6.88E+02,1.57E+03,2.75E+02,2.07E+03,1.62E+03,1.66E+03,1.65E+03,1.3
375.16636,7.27E+02,1.22E+03,9.78E+02,1.33E+03,3.10E+01,2.00E+03,9.37E+02,2.01E+02,4.49E+02,1.
375.20289,1.76E+03,6.46E+02,1.60E+03,9.59E+02,1.17E+03,8.92E+02,4.91E+02,1.59E+03,1.10E+03,2.
377.08471,1.14E+04,2.68E+04,1.31E+04,1.97E+04,2.40E+04,2.90E+04,1.18E+04,2.49E+04,2.25E+04,3.
378.08807,1.59E+03,3.88E+03,2.26E+03,3.24E+03,2.39E+03,5.20E+03,2.11E+03,3.77E+03,2.68E+03,4.
378.14011,2.23E+03,1.60E+03,2.08E+03,1.37E+02,1.59E+02,1.95E+03,2.79E+03,2.82E+03,1.79E+03,2.
379.08147,4.26E+03,9.34E+03,4.86E+03,8.60E+03,7.73E+03,1.17E+04,4.95E+03,8.46E+03,7.19E+03,9.
379.08501,3.81E+03,7.01E+03,3.71E+03,7.44E+03,5.90E+03,1.13E+04,1.89E+02,7.83E+03,5.90E+03,8.
379.15301,2.25E+03,2.33E+02,1.12E+02,3.33E+03,9.87E+02,6.13E+02,3.40E+02,8.38E+02,1.81E+03,1.
380.00255,3.21E+03,2.33E+02,3.36E+03,2.26E+03,2.69E+02,3.41E+02,6.68E+02,1.45E+03,1.81E+03,1.
380.08592,5.56E+02,1.99E+03,2.02E+03,2.41E+03,1.32E+03,1.76E+03,1.80E+03,2.81E+03,1.99E+03,1.
380.15562,2.70E+03,3.16E+03,6.71E+02,1.50E+03,1.54E+03,1.94E+03,2.66E+03,2.12E+03,1.81E+03,7.
381.23015,1.80E+02,2.77E+02,6.71E+02,9.31E+02,1.34E+03,6.22E+02,1.57E+02,2.43E+02,7.21E+02,7.
383.12681,1.69E+03,2.11E+03,2.78E+03,1.61E+03,8.52E+02,1.88E+03,1.74E+03,1.26E+03,2.11E+03,1.
384.986,2.73E+03,2.06E+03,2.29E+03,1.61E+03,1.33E+04,2.28E+03,1.32E+03,1.34E+03,2.25E+03,7.02
385.18716,1.20E+03,1.36E+03,1.09E+02,1.45E+03,7.49E+02,1.98E+03,7.12E+02,8.34E+02,2.07E+03,1.
386.12976,2.42E+03,2.11E+03,2.32E+03,6.05E+02,6.01E+02,1.32E+03,2.02E+03,1.21E+03,1.83E+03,1.
386.98335,1.07E+03,2.29E+03,3.29E+03,1.31E+03,7.22E+03,1.81E+03,1.49E+03,2.10E+03,4.65E+02,3.

386.98588,9.98E+02,9.35E+02,3.29E+03,6.71E+02,5.75E+03,1.81E+03,1.19E+03,1.45E+03,4.65E+02,3.
387.11476,8.42E+02,1.81E+03,1.41E+03,1.50E+03,6.79E+02,3.91E+02,7.09E+02,1.80E+03,1.68E+03,5.
387.15072,1.84E+03,1.73E+03,1.81E+03,1.12E+03,1.91E+03,8.34E+02,2.24E+03,1.08E+03,1.72E+03,1.
387.16627,1.25E+03,5.95E+01,8.63E+01,1.61E+03,2.29E+03,1.44E+03,1.54E+03,1.42E+03,3.26E+03,9.
387.20208,1.63E+03,1.61E+03,1.91E+03,1.55E+03,1.59E+03,1.29E+03,1.62E+03,1.69E+03,1.09E+03,1.
388.08874,1.99E+03,1.41E+03,2.65E+03,4.42E+02,1.03E+02,1.65E+03,2.52E+03,5.18E+02,1.76E+03,2.
388.19768,1.93E+03,2.08E+03,8.97E+02,2.30E+03,2.26E+03,2.03E+03,2.04E+03,3.45E+03,1.56E+03,8.
388.98247,1.41E+03,3.25E+03,2.77E+03,1.78E+02,5.50E+03,1.73E+03,1.01E+02,2.61E+02,9.99E+02,2.
389.1808,2.20E+01,1.20E+03,8.07E+02,1.36E+03,1.17E+03,1.25E+03,1.78E+03,6.91E+03,1.26E+03,1.1
389.21814,1.33E+03,2.40E+03,1.51E+03,1.33E+03,3.09E+03,1.94E+03,1.81E+03,1.40E+03,1.60E+03,1.
390.14005,1.70E+03,1.57E+03,4.61E+02,1.21E+03,3.21E+02,9.95E+02,2.83E+03,2.85E+02,1.58E+03,2.
390.15099,1.71E+03,7.39E+02,3.43E+03,6.65E+02,1.01E+04,3.30E+03,5.30E+02,1.42E+03,1.10E+03,1.
390.15435,9.84E+02,3.01E+02,3.43E+03,5.38E+02,7.84E+03,3.30E+03,5.30E+02,1.52E+03,4.26E+02,1.
390.21305,1.34E+03,7.24E+02,3.20E+01,1.97E+03,6.20E+00,1.48E+03,3.41E+03,2.37E+03,1.75E+03,1.
391.15502,1.80E+03,4.54E+02,7.29E+02,2.24E+03,1.60E+03,4.95E+02,1.91E+03,2.66E+03,7.27E+02,2.
392.15572,2.12E+03,2.17E+03,6.18E+02,2.66E+03,1.80E+03,2.03E+03,1.95E+03,2.29E+03,2.50E+03,2.
392.26517,1.82E+03,1.56E+03,2.06E+03,1.75E+03,1.09E+02,1.37E+02,5.02E+02,6.83E+00,1.75E+03,2.
393.0521,1.82E+03,1.59E+03,7.09E+02,1.18E+03,2.37E+03,1.65E+03,5.02E+02,2.30E+03,1.52E+03,2.2
393.16947,1.72E+03,1.42E+03,6.94E+02,1.72E+03,1.90E+03,1.79E+03,1.07E+03,1.44E+03,1.81E+03,1.
393.27592,2.70E+03,3.23E+03,3.29E+03,3.65E+03,8.67E+00,3.13E+03,5.80E+01,4.16E+03,3.70E+03,5.
394.02094,2.15E+03,1.76E+02,2.08E+03,1.32E+03,1.33E+03,2.48E+02,5.80E+01,4.59E+01,3.15E+02,5.
394.13491,1.66E+03,1.60E+03,2.00E+00,1.82E+03,2.81E+03,7.59E+02,5.80E+01,1.59E+03,2.85E+01,1.
394.17155,2.60E+03,2.51E+03,2.00E+00,1.91E+03,5.48E+02,1.85E+03,1.69E+03,2.05E+03,2.45E+03,1.
394.28058,1.94E+03,1.79E+03,2.00E+03,2.14E+02,2.74E+03,2.53E+02,1.89E+02,2.22E+03,1.73E+03,1.
395.27374,2.00E+03,2.05E+03,2.49E+03,2.77E+03,2.00E+03,2.24E+03,4.49E+02,3.16E+03,1.07E+03,4.
395.38981,6.37E+02,6.45E+02,5.15E+02,7.66E+02,2.68E+02,2.19E+02,4.26E+02,6.31E+02,4.93E+02,8.
396.03273,2.99E+03,6.69E+02,3.09E+03,1.61E+03,1.13E+02,3.70E+01,3.31E+03,2.96E+03,1.20E+01,4.
396.15019,1.71E+03,2.04E+03,2.73E+03,9.78E+02,1.13E+02,1.05E+03,1.58E+03,1.91E+03,2.87E+03,1.
396.39321,1.17E+02,2.04E+02,2.18E+02,1.95E+02,8.02E+02,4.22E+02,9.40E+01,2.15E+02,1.79E+02,1.
397.22451,9.13E+02,4.38E+02,3.99E+02,2.52E+03,2.50E+01,4.22E+02,1.24E+03,1.51E+03,1.10E+03,1.
399.20198,9.67E+02,1.68E+03,6.50E+02,4.41E+02,2.92E+03,1.75E+03,1.15E+02,4.34E+02,3.78E+02,4.
403.19704,1.75E+03,1.81E+03,6.89E+02,1.45E+03,2.96E+02,1.68E+03,8.62E+02,1.63E+03,9.97E+02,1.
404.10367,2.17E+04,3.71E+04,4.73E+04,2.20E+04,9.26E+03,2.59E+04,2.85E+04,2.28E+04,3.10E+04,1.
404.10652,1.61E+04,2.68E+04,3.62E+04,1.67E+04,7.45E+03,2.03E+04,2.40E+04,1.65E+04,2.41E+04,1.
404.15558,2.32E+03,4.08E+02,4.91E+02,2.25E+03,1.68E+03,1.04E+03,1.27E+03,1.59E+03,3.03E+03,1.
404.19233,1.92E+03,2.41E+03,4.91E+02,3.00E+02,1.75E+03,1.85E+03,2.98E+03,2.79E+03,1.73E+03,1.
405.10667,2.49E+03,2.64E+03,4.91E+02,3.55E+03,1.44E+03,3.31E+02,2.70E+03,2.88E+03,6.56E+02,1.
405.11025,1.50E+01,5.82E+03,4.91E+02,1.75E+03,1.01E+03,3.31E+02,3.70E+03,1.28E+03,7.76E+01,8.
405.15064,1.36E+03,1.65E+03,2.16E+03,1.58E+03,2.84E+03,1.73E+03,3.00E+01,2.57E+03,1.34E+03,1.
406.10928,2.60E+03,3.60E+01,1.57E+03,1.49E+03,6.63E+02,1.61E+03,1.65E+03,1.58E+03,1.27E+02,1.
406.13545,4.01E+02,3.60E+01,3.69E+02,1.42E+03,6.63E+02,8.35E+01,3.09E+03,2.55E+03,1.42E+03,1.
406.17127,4.01E+02,2.03E+03,3.69E+02,1.92E+03,4.18E+02,1.42E+03,1.63E+02,1.64E+03,2.95E+03,2.
407.06753,1.54E+03,2.32E+03,1.87E+03,9.23E+01,2.44E+03,1.52E+03,1.38E+03,1.26E+03,1.85E+03,1.
407.42699,1.36E+02,1.47E+02,2.09E+02,5.70E+02,1.16E+02,9.40E+01,1.33E+02,1.67E+02,8.05E+02,1.

408.14982,2.33E+03,2.12E+03,1.20E+01,1.84E+03,1.94E+03,2.55E+03,3.26E+03,1.64E+03,2.25E+03,1.
409.16229,1.22E+03,3.26E+03,1.20E+01,1.08E+03,2.89E+03,1.83E+03,2.74E+02,1.82E+03,2.55E+03,1.
410.16592,1.82E+03,3.30E+03,1.90E+03,1.46E+03,1.32E+03,2.38E+02,1.32E+03,1.82E+03,1.42E+03,1.
413.21775,1.70E+03,2.13E+03,8.28E+02,1.01E+03,1.35E+03,9.70E+02,1.65E+03,2.03E+03,9.88E+02,1.
415.2337,1.51E+03,3.30E+03,7.82E+02,1.83E+03,5.61E+02,1.94E+03,1.79E+03,1.52E+03,2.03E+03,1.4
417.21247,4.60E+01,1.46E+02,4.55E+02,7.82E+02,2.97E+03,1.08E+03,5.63E+02,1.34E+03,7.48E+02,2.
418.17078,4.62E+02,2.15E+03,9.65E+02,2.38E+03,1.75E+03,1.75E+03,1.39E+03,2.84E+03,1.82E+03,1.
420.00064,7.56E+02,4.70E+01,9.65E+02,4.86E+02,9.19E+02,8.41E+02,3.17E+02,3.50E+01,8.80E+01,2.
420.15033,1.89E+03,2.93E+03,9.65E+02,1.72E+03,9.19E+02,6.59E+02,1.95E+03,2.08E+03,1.90E+03,9.
420.1864,3.40E+03,2.45E+03,9.65E+02,2.63E+03,3.14E+03,1.74E+03,3.24E+03,2.44E+03,2.04E+03,2.6
420.29521,2.51E+03,4.84E+01,3.83E+03,3.51E+02,3.71E+02,2.86E+02,8.65E+01,2.59E+02,1.83E+03,1.
421.16189,2.42E+03,2.02E+03,1.64E+03,3.51E+02,6.40E+02,1.68E+03,5.49E+02,1.23E+03,1.72E+03,1.
421.99797,6.73E+02,2.12E+02,8.22E+02,6.75E+02,1.90E+01,4.77E+02,4.31E+02,6.93E+02,1.04E+03,9.
422.01699,6.73E+02,2.12E+02,8.22E+02,6.75E+02,1.90E+01,4.77E+02,4.31E+02,6.93E+02,1.04E+03,9.
422.16513,2.23E+03,1.71E+03,8.22E+02,1.68E+03,9.30E+02,1.45E+03,3.12E+03,2.39E+03,2.33E+03,1.
422.20157,2.15E+03,1.65E+02,8.22E+02,1.34E+03,1.07E+02,1.76E+03,2.45E+02,1.65E+03,2.45E+03,1.
423.17765,2.36E+03,2.33E+02,6.59E+02,2.45E+03,2.34E+03,1.28E+03,2.73E+03,1.96E+02,1.35E+03,1.
423.42145,1.32E+02,2.20E+02,2.18E+02,4.06E+02,9.70E+01,2.55E+01,2.93E+02,1.94E+02,8.90E+01,1.
424.02236,2.92E+03,2.30E+01,2.39E+03,1.70E+01,1.49E+03,2.55E+01,2.93E+02,6.08E+02,2.60E+01,1.
425.19338,2.49E+03,1.60E+03,1.63E+03,1.60E+03,1.31E+03,7.54E+02,1.92E+03,2.90E+03,1.32E+03,2.
429.17573,2.28E+03,1.38E+03,9.87E+02,1.20E+03,1.95E+03,7.10E+02,1.06E+03,4.95E+02,1.58E+03,8.
429.21181,3.59E+02,1.61E+03,2.28E+02,1.38E+03,3.55E+02,1.60E+03,2.45E+03,1.25E+03,1.83E+03,5.
431.11891,7.52E+02,1.69E+03,3.57E+02,1.60E+03,1.43E+03,1.91E+03,1.57E+03,1.26E+03,1.29E+03,7.
431.1922,1.99E+03,2.98E+02,3.57E+02,8.18E+02,1.60E+03,2.19E+03,1.38E+03,1.56E+03,2.14E+02,1.5
431.22717,8.10E+02,3.05E+03,1.57E+03,1.41E+03,8.67E+02,3.97E+03,3.51E+03,1.37E+03,3.60E+03,1.
432.11416,8.67E+02,6.07E+02,2.33E+03,1.12E+03,1.85E+03,1.77E+03,7.90E+02,2.11E+03,9.72E+02,1.
432.99533,1.70E+03,1.83E+03,2.43E+02,2.32E+03,3.61E+03,2.14E+03,1.15E+03,1.90E+03,2.07E+03,1.
434.16552,2.91E+03,1.64E+03,8.05E+01,1.56E+03,7.73E+01,1.19E+03,5.39E+02,1.80E+03,1.98E+03,1.
434.20176,1.50E+03,2.55E+03,8.05E+01,1.55E+01,1.33E+03,2.03E+03,2.45E+03,2.19E+03,2.26E+03,2.
434.99209,1.34E+03,1.08E+03,1.06E+03,1.77E+03,5.78E+02,1.64E+03,7.97E+02,1.46E+03,9.14E+02,1.
435.22258,2.87E+02,1.68E+03,3.88E+02,7.40E+01,5.21E+02,1.93E+03,1.89E+03,4.15E+02,1.81E+03,1.
436.1807,1.74E+02,3.51E+03,3.88E+02,1.99E+03,2.20E+03,1.43E+03,3.07E+03,2.29E+03,2.13E+03,1.6
436.99124,1.43E+03,8.31E+02,3.88E+02,6.41E+02,1.68E+03,1.11E+02,5.96E+02,7.08E+02,5.59E+02,1.
437.16459,1.57E+03,1.36E+03,9.15E+01,2.32E+03,1.11E+03,1.11E+02,5.96E+02,1.72E+03,1.74E+03,1.
437.18421,1.92E+03,1.91E+03,9.15E+01,1.44E+03,1.83E+03,1.79E+03,1.52E+03,2.52E+03,1.37E+03,6.
438.15973,2.32E+03,1.51E+03,2.32E+03,1.73E+03,2.13E+03,2.04E+03,1.87E+03,2.42E+03,1.54E+03,1.
438.19648,1.47E+03,1.33E+03,1.26E+02,2.08E+03,1.27E+03,1.10E+03,2.24E+03,1.82E+03,2.56E+03,1.
439.07447,1.61E+03,3.08E+03,3.97E+02,9.01E+02,4.66E+03,1.59E+03,1.80E+03,3.25E+03,3.62E+03,3.
439.08364,1.68E+03,8.34E+01,3.97E+02,3.79E+03,7.56E+03,3.67E+03,3.69E+03,2.90E+03,3.20E+03,5.
440.08942,6.13E+02,6.00E+02,2.52E+03,1.88E+03,9.10E+00,2.39E+03,6.71E+02,7.84E+02,1.42E+03,1.
440.17757,1.66E+03,1.55E+03,2.77E+03,1.80E+03,2.14E+03,1.67E+03,2.08E+03,1.78E+03,1.10E+03,1.
441.18728,5.15E+02,5.05E+02,1.88E+03,3.72E+02,8.62E+02,1.12E+03,3.67E+02,4.67E+02,1.44E+02,1.
441.25147,7.99E+02,5.05E+02,7.20E+01,1.05E+03,2.16E+03,1.29E+03,1.50E+03,1.63E+03,1.92E+03,1.
445.11843,3.49E+02,4.88E+03,3.51E+03,2.49E+03,5.22E+03,1.09E+04,2.00E+03,2.00E+03,2.75E+03,2.

446.12217,4.70E+02,9.32E+02,2.76E+03,4.25E+02,2.18E+03,2.79E+03,7.57E+02,6.81E+02,3.54E+02,1.
447.01113,1.75E+03,1.78E+03,1.35E+03,1.20E+03,2.01E+03,2.06E+03,1.12E+03,1.55E+03,1.46E+03,1.
447.22223,1.95E+03,1.35E+03,2.15E+01,2.11E+03,1.41E+03,1.37E+03,1.81E+03,1.43E+03,1.66E+01,1.
448.181,3.38E+03,2.53E+03,2.24E+03,4.11E+02,2.62E+02,2.06E+03,4.19E+03,2.36E+03,2.92E+03,1.39
449.00812,1.46E+03,1.36E+03,6.66E+02,2.50E+03,2.21E+03,1.87E+03,1.58E+03,1.97E+03,2.23E+03,1.
449.18403,1.74E+03,7.38E+02,2.16E+03,1.45E+03,4.90E+02,1.26E+03,7.64E+02,1.89E+03,1.89E+03,9.
449.20132,2.94E+02,1.71E+03,2.13E+03,1.72E+03,4.90E+02,6.08E+02,1.05E+03,1.47E+03,1.08E+03,1.
450.16032,1.64E+03,2.23E+03,8.54E+02,1.25E+03,1.17E+03,3.15E+02,1.98E+03,2.08E+03,1.79E+03,2.
450.19582,4.77E+03,2.58E+03,2.41E+03,2.33E+03,1.28E+03,2.89E+03,4.87E+03,4.06E+02,2.95E+03,1.
451.00611,2.49E+03,6.46E+02,4.50E+00,7.32E+02,1.03E+03,1.81E+03,8.07E+02,1.14E+03,2.59E+03,1.
451.01719,1.12E+02,2.30E+03,4.50E+00,1.97E+03,9.91E+02,1.42E+02,3.94E+02,1.30E+03,7.40E+02,1.
451.20028,1.38E+03,1.52E+03,8.88E+01,9.68E+02,3.16E+02,2.44E+03,2.20E+03,1.57E+03,1.05E+03,1.
452.17544,3.70E+02,2.17E+03,2.83E+03,2.48E+03,3.31E+03,4.24E+03,2.34E+03,5.64E+03,3.16E+03,2.
452.21141,4.01E+03,2.80E+03,4.41E+03,2.11E+03,2.51E+03,1.29E+03,3.34E+03,1.80E+03,2.49E+03,2.
453.18025,1.13E+03,5.02E+02,1.80E+03,1.99E+03,1.39E+03,1.42E+03,4.16E+01,1.61E+03,8.22E+02,1.
454.1914,2.25E+03,1.38E+03,3.13E+03,1.64E+03,1.81E+03,1.05E+03,1.91E+03,2.58E+03,1.09E+03,2.0
457.13495,2.65E+01,1.82E+03,2.84E+03,1.17E+03,1.96E+03,2.22E+03,1.09E+03,8.00E+02,2.00E+03,6.
461.1837,2.88E+03,3.33E+03,1.96E+03,1.94E+03,1.29E+03,2.07E+03,8.20E+02,1.07E+02,2.52E+03,2.2
462.19309,9.83E+02,1.75E+03,1.80E+03,1.29E+03,9.54E+02,1.20E+03,1.93E+03,1.51E+03,1.41E+03,1.
462.2334,2.10E+03,2.08E+03,4.30E+01,9.46E+01,2.02E+03,2.90E+02,1.54E+03,1.71E+03,1.81E+03,1.2
463.21655,1.98E+03,1.16E+03,2.27E+03,1.27E+03,1.58E+03,1.46E+03,1.23E+03,3.20E+03,1.81E+03,1.
464.17572,2.51E+03,1.88E+03,3.18E+01,2.18E+03,1.12E+02,2.09E+03,3.39E+03,1.32E+03,1.92E+03,1.
464.2123,2.00E+03,1.38E+03,3.18E+01,1.25E+02,1.52E+03,1.74E+03,1.40E+03,1.17E+03,2.04E+03,1.5
465.19545,1.12E+03,1.07E+03,1.81E+03,2.82E+03,2.63E+03,2.81E+03,2.05E+03,1.25E+03,2.16E+03,1.
466.19107,1.40E+03,2.85E+03,9.76E+02,3.00E+03,1.67E+03,1.40E+03,2.63E+03,2.20E+03,1.84E+03,1.
467.21049,5.05E+03,6.45E+03,9.63E+03,5.40E+03,1.58E+03,8.31E+03,1.31E+04,5.67E+03,1.41E+04,4.
468.21453,1.90E+03,1.46E+03,2.51E+03,1.60E+03,7.75E+01,1.53E+03,3.00E+03,9.89E+02,2.51E+03,7.
470.25115,3.68E+02,1.89E+03,8.00E+02,1.28E+03,1.52E+03,2.36E+02,1.52E+03,2.08E+03,8.84E+02,5.
472.0944,1.90E+03,7.09E+02,2.61E+03,1.46E+02,1.12E+02,1.69E+03,4.10E+02,1.70E+03,7.34E+02,1.6
473.16097,1.51E+03,3.00E+03,3.07E+03,1.68E+03,1.05E+04,2.76E+03,1.85E+03,4.78E+01,2.43E+03,6.
474.14506,2.85E+03,1.95E+03,2.39E+03,3.13E+02,1.69E+03,1.59E+03,1.18E+02,3.22E+02,2.69E+03,1.
474.16515,7.51E+02,6.18E+01,9.00E+02,1.35E+03,1.98E+02,1.23E+02,7.91E+02,1.16E+03,1.72E+03,1.
475.12955,7.51E+02,2.16E+02,2.85E+03,2.30E+01,1.13E+03,1.62E+03,6.40E+02,4.00E+02,3.83E+02,1.
476.2129,2.22E+03,5.46E+02,3.04E+02,2.93E+03,3.46E+02,1.60E+03,2.19E+03,1.57E+03,1.47E+02,1.5
477.0311,4.02E+02,1.94E+03,2.15E+03,4.04E+02,3.46E+02,3.55E+02,2.69E+03,1.10E+02,1.47E+02,1.6
477.19592,1.64E+03,3.60E+02,3.30E+02,1.66E+03,1.71E+03,9.53E+02,2.27E+03,2.34E+03,1.47E+02,1.
478.03585,1.16E+02,2.31E+03,3.98E+03,2.28E+03,5.71E+02,1.53E+03,2.06E+03,2.08E+03,2.64E+03,1.
478.22746,2.83E+03,1.80E+03,2.02E+02,2.54E+03,1.56E+03,1.67E+03,2.36E+03,1.59E+03,1.82E+03,1.
478.24802,3.08E+03,2.05E+03,3.01E+03,2.92E+03,3.44E+03,1.69E+03,2.35E+03,3.15E+03,1.83E+03,2.
478.74991,1.82E+03,1.56E+03,1.91E+03,2.22E+03,2.06E+03,2.00E+03,2.06E+03,3.36E+03,1.26E+02,7.
480.20729,2.37E+03,2.56E+03,8.89E+02,1.82E+03,1.23E+02,7.05E+02,1.73E+03,1.95E+03,1.95E+03,1.
481.22726,1.92E+03,3.16E+03,2.97E+03,2.17E+03,1.89E+03,1.40E+03,3.25E+03,1.99E+03,2.89E+03,2.
481.2554,2.01E+03,1.70E+02,6.10E+01,1.65E+03,5.17E+03,2.66E+03,2.71E+01,5.08E+03,1.22E+03,1.1
482.22219,3.40E+03,3.92E+02,2.76E+03,1.75E+03,3.97E+02,6.55E+02,1.63E+03,1.68E+03,4.37E+02,1.

488.16033,2.12E+03,2.66E+03,2.94E+03,1.73E+03,3.31E+03,3.87E+03,1.76E+03,3.55E+03,2.04E+03,2.
489.16431,9.74E+02,5.07E+02,1.03E+02,2.16E+02,3.70E+01,1.83E+03,8.75E+02,1.92E+03,3.59E+02,1.
491.16042,3.72E+02,5.26E+02,1.03E+02,1.70E+03,1.49E+03,2.72E+03,7.19E+02,7.75E+02,2.76E+03,1.
491.25661,1.41E+03,4.80E+02,1.12E+02,1.64E+03,3.45E+03,7.85E+01,1.36E+03,2.08E+03,4.86E+02,4.
492.20732,1.99E+03,3.37E+03,1.12E+02,1.66E+03,2.12E+03,1.51E+03,3.58E+03,3.12E+03,2.52E+03,1.
494.22256,2.03E+03,1.40E+03,2.75E+02,7.45E+02,4.65E+02,2.27E+03,2.02E+03,1.65E+03,1.64E+03,2.
499.25287,2.60E+03,1.22E+03,1.40E+03,2.89E+03,2.37E+03,2.01E+03,2.23E+03,4.49E+03,1.49E+03,1.
499.75461,2.06E+03,2.07E+02,2.59E+03,2.53E+03,2.39E+03,1.67E+03,1.49E+03,2.91E+03,1.85E+03,4.
509.22208,1.86E+03,2.11E+03,2.62E+03,1.76E+03,1.31E+03,1.73E+03,2.98E+03,1.53E+03,2.56E+03,1.
510.2177,4.79E+03,2.74E+03,1.15E+02,8.95E+02,8.64E+02,5.37E+02,2.40E+03,2.34E+03,1.52E+03,1.3
512.03702,3.37E+03,7.74E+02,1.50E+03,1.23E+03,2.63E+03,1.58E+03,8.30E+01,2.41E+03,6.03E+02,1.
513.30451,3.02E+02,1.91E+02,1.04E+03,2.07E+03,1.63E+03,4.39E+02,2.84E+02,4.83E+03,1.28E+03,1.
514.03411,1.71E+03,1.91E+02,1.80E+03,1.93E+03,2.16E+03,1.40E+03,2.84E+02,6.53E+02,1.02E+02,1.
517.161,1.55E+03,1.39E+03,1.88E+03,4.37E+02,5.06E+03,1.65E+03,3.84E+02,3.01E+02,2.48E+02,4.35
517.1896,1.81E+03,3.18E+03,2.67E+03,2.20E+03,7.04E+02,2.15E+03,3.84E+02,4.68E+02,2.93E+03,1.2
517.20043,4.10E+02,1.38E+03,1.31E+03,5.44E+02,2.47E+03,1.18E+03,3.84E+02,2.60E+03,1.14E+03,2.
519.21636,1.81E+03,4.31E+02,5.11E+03,3.04E+02,2.25E+03,1.32E+03,3.84E+02,1.74E+03,1.01E+02,2.
519.24878,8.84E+03,6.31E+03,5.32E+03,1.91E+04,3.48E+04,5.66E+03,4.33E+03,2.22E+04,5.38E+03,2.
519.75072,4.75E+03,2.93E+03,3.03E+03,1.10E+04,2.24E+04,2.89E+03,3.73E+03,1.44E+04,3.00E+03,2.
520.23605,3.65E+02,6.67E+02,2.30E+02,8.51E+02,1.11E+02,3.65E+02,2.78E+02,1.78E+03,1.15E+03,2.
520.25318,2.44E+03,6.67E+02,4.15E+01,2.87E+03,5.39E+03,1.89E+03,1.78E+03,3.59E+03,1.74E+03,2.
521.16997,1.26E+03,1.46E+03,9.73E+02,1.48E+03,5.08E+01,2.59E+03,1.64E+02,4.00E+01,9.88E+02,1.
521.26523,1.46E+04,1.08E+04,1.38E+04,2.31E+04,3.52E+04,9.59E+03,7.73E+03,3.15E+04,1.08E+04,7.
521.76693,8.31E+03,6.43E+03,9.71E+03,1.21E+04,1.94E+04,6.39E+03,6.63E+03,1.65E+04,6.70E+03,5.
522.26908,3.21E+03,3.67E+01,3.19E+03,3.50E+03,6.22E+03,3.26E+03,2.33E+03,5.55E+03,2.98E+03,1.
522.77155,1.23E+02,3.67E+01,1.43E+03,1.02E+03,3.20E+03,1.67E+03,2.46E+02,1.65E+03,1.29E+03,1.
523.23736,1.23E+02,1.89E+03,1.89E+03,9.90E+01,1.75E+03,4.10E+01,1.67E+03,6.74E+01,1.79E+03,1.
535.26287,3.22E+03,1.66E+03,2.85E+03,4.82E+03,4.10E+03,1.28E+03,2.91E+03,4.46E+03,1.60E+03,3.
535.76438,1.56E+03,1.56E+03,1.00E+03,2.83E+03,3.56E+03,9.13E+01,1.56E+03,3.98E+03,1.10E+03,3.
536.26779,1.82E+03,1.80E+03,2.00E+03,1.66E+03,8.01E+01,9.13E+01,1.44E+03,9.19E+02,2.14E+03,1.
549.28081,2.75E+03,5.58E+02,8.78E+02,3.69E+03,2.17E+03,2.37E+02,6.29E+02,5.93E+03,1.92E+03,4.
554.18533,2.15E+03,4.98E+02,3.55E+03,5.53E+02,1.89E+03,2.65E+03,7.37E+02,5.07E+02,3.53E+02,1.
576.29854,6.08E+03,1.89E+03,4.64E+03,4.74E+03,2.00E+03,7.48E+02,6.89E+02,8.15E+03,1.75E+03,4.
577.15239,1.03E+04,1.35E+04,1.91E+04,1.22E+04,1.56E+04,1.32E+04,5.64E+03,1.59E+04,1.30E+04,9.
578.15606,2.86E+03,2.84E+03,5.12E+03,3.63E+03,3.33E+03,3.75E+03,2.64E+03,4.38E+03,3.73E+03,3.
593.1483,2.45E+03,3.47E+03,3.14E+03,2.66E+03,1.68E+03,2.38E+03,1.78E+03,2.87E+03,2.78E+03,1.7
613.12919,5.59E+03,5.98E+03,5.96E+03,9.10E+03,8.42E+03,7.68E+03,2.78E+03,9.74E+03,7.34E+03,6.
614.13296,2.15E+03,2.13E+03,2.50E+03,4.29E+03,2.63E+03,2.61E+03,3.65E+03,2.60E+03,2.98E+03,2.
615.12667,1.88E+03,1.50E+03,3.14E+03,3.89E+03,3.87E+03,2.85E+03,1.76E+02,3.29E+03,1.78E+03,1.
639.17277,1.10E+03,7.53E+02,8.61E+02,2.10E+03,3.52E+03,1.63E+03,1.00E+00,1.34E+03,1.95E+03,2.
640.14744,2.41E+04,2.50E+04,3.56E+04,2.22E+04,9.57E+03,1.48E+04,1.89E+04,1.89E+04,1.61E+04,9.
641.15078,8.85E+03,6.94E+03,1.09E+04,6.35E+03,4.03E+03,4.13E+03,5.19E+03,6.34E+03,5.20E+03,2.
642.15382,7.04E+01,2.38E+03,2.79E+03,3.30E+03,7.54E+02,2.19E+03,2.03E+03,1.99E+03,2.06E+03,1.
675.12923,7.74E+02,6.89E+02,2.55E+03,1.62E+03,2.00E+03,1.68E+03,1.82E+03,1.36E+03,1.57E+03,1.

681.16385,2.42E+03,3.40E+03,2.93E+03,4.28E+03,4.02E+03,5.14E+03,2.69E+03,3.36E+03,2.74E+03,1.
741.46798,3.48E+03,4.00E+03,4.94E+03,1.69E+03,2.01E+03,1.62E+03,1.14E+03,2.40E+03,2.95E+03,2.
742.47137,2.94E+03,1.88E+03,3.19E+03,1.38E+03,1.81E+03,1.01E+03,1.29E+03,3.00E+03,2.18E+03,1.
809.49204,3.94E+03,8.19E+02,3.18E+03,2.26E+03,2.55E+03,2.27E+03,9.31E+02,2.15E+03,3.13E+03,1.
815.49342,9.62E+02,3.33E+03,3.24E+03,1.59E+03,2.47E+03,2.39E+03,1.69E+03,2.05E+03,4.14E+03,1.
836.51232,4.26E+03,4.59E+03,9.91E+03,2.49E+03,7.20E+02,3.88E+03,3.20E+03,2.32E+03,6.11E+03,2.
837.51566,3.69E+03,2.23E+03,6.72E+03,2.30E+03,5.80E+02,1.78E+03,2.39E+03,1.63E+03,2.18E+03,1.
923.55309,1.06E+02,1.75E+02,1.57E+02,2.09E+02,7.58E+02,4.74E+02,2.05E+02,2.79E+02,1.51E+02,9.

3E+03,1.12E+03,7.64E+02
6E+02,1.09E+03,8.00E+02
3E+04,2.48E+04,1.52E+04
2E+03,7.23E+03,2.85E+03
4E+02,2.57E+02,7.63E+02
1E+02,9.25E+02,7.06E+02
4E+02,1.09E+03,7.17E+02
7E+02,5.00E+02,3.50E+02
8E+02,5.26E+02,5.47E+02
8E+02,8.83E+02,7.59E+02
8E+02,7.28E+02,4.00E+02
8E+02,9.79E+02,6.94E+02
1E+02,5.40E+02,3.09E+02
8E+02,8.84E+02,6.40E+02
4E+02,7.38E+02,7.21E+02
8E+02,5.74E+02,5.74E+02
0E+02,7.80E+02,5.94E+02
7E+02,9.46E+02,6.33E+02
7E+02,2.93E+02,6.40E+02
7E+02,2.93E+02,6.40E+02
7E+02,2.93E+02,6.40E+02
2E+02,2.93E+02,6.40E+02
2E+03,9.52E+02,1.41E+02
8E+02,4.71E+02,6.38E+02
8E+02,1.15E+03,5.29E+02
3E+02,5.65E+02,6.30E+02
8E+02,5.65E+02,6.02E+02
8E+02,9.13E+02,5.16E+02
8E+02,3.17E+02,2.38E+02
0E+02,9.91E+02,6.26E+02
0E+02,9.38E+02,3.32E+02
9E+03,7.11E+02,6.94E+02
5E+02,7.11E+02,7.93E+01
5E+02,5.18E+02,8.38E+02
2E+02,7.99E+02,4.75E+02
2E+02,7.69E+02,5.71E+02
2E+02,8.78E+02,7.41E+02
2E+02,1.07E+03,3.25E+02
2E+02,9.63E+02,3.25E+02
2E+02,8.40E+02,3.25E+02
1E+02,3.46E+02,2.79E+02
1E+02,3.46E+02,5.74E+02
1E+02,8.24E+02,6.34E+02

4E+02,3.94E+02,7.30E+02
4E+02,8.32E+02,3.46E+02
6E+02,1.00E+03,3.46E+02
E+02,4.88E+02,5.82E+02
3E+02,1.12E+03,7.18E+02
3E+02,3.80E+02,1.34E+02
6E+02,9.41E+02,5.95E+02
6E+02,1.09E+03,6.53E+02
6E+02,1.01E+03,1.44E+02
6E+02,8.66E+02,1.44E+02
8E+02,3.20E+02,1.44E+02
4E+02,9.82E+02,5.74E+02
E+02,8.40E+02,5.60E+02
E+02,4.84E+02,3.63E+02
0E+02,5.75E+02,3.68E+02
E+02,2.46E+02,3.17E+02
E+02,2.85E+02,4.32E+02
2E+02,3.70E+02,3.93E+02
+04,4.93E+04,3.28E+04
4E+02,2.28E+03,1.13E+03
8E+02,9.40E+01,1.04E+02
8E+03,1.27E+03,8.87E+02
8E+03,1.27E+03,9.18E+02
E+05,1.53E+05,1.98E+05
3E+03,2.90E+03,1.36E+04
4E+03,2.07E+03,2.79E+03
6E+04,1.19E+04,1.30E+04
4E+04,9.99E+03,2.51E+04
3E+04,1.27E+04,3.61E+04
7E+05,1.40E+05,6.21E+04
9E+03,2.53E+03,7.64E+02
E+02,3.31E+02,3.64E+02
4E+03,8.00E+00,5.31E+02
7E+05,7.27E+04,1.35E+05
6E+04,2.06E+04,6.17E+03
2E+03,1.20E+03,1.17E+03
5E+03,1.31E+03,1.04E+03
2E+03,2.82E+03,4.33E+03
3E+03,1.73E+03,2.40E+03
1E+02,8.62E+02,6.76E+02
9E+02,4.45E+02,2.77E+02
4E+04,3.79E+04,4.14E+04
6E+02,1.03E+03,6.62E+02
9E+03,1.20E+03,8.70E+02

2E+02,1.47E+02,7.15E+02
E+05,6.94E+04,8.27E+04
4E+03,2.23E+03,2.65E+03
9E+03,1.19E+03,1.15E+03
8E+04,2.35E+04,2.51E+04
0E+01,1.23E+02,6.30E+01
9E+03,1.60E+03,7.51E+02
1E+02,1.50E+02,1.86E+02
6E+05,1.10E+05,9.39E+04
5E+04,1.03E+04,7.95E+04
1E+03,2.06E+03,1.92E+03
7E+02,1.12E+03,9.70E+01
5E+03,1.88E+03,7.81E+02
8E+03,5.20E+03,2.71E+03
8E+03,7.09E+02,6.41E+02
0E+03,3.76E+02,8.24E+02
4E+02,6.22E+02,5.29E+02
8E+03,1.23E+03,7.84E+02
5E+03,4.69E+03,5.86E+02
1E+03,1.34E+03,1.85E+03
0E+03,2.55E+03,1.18E+04
E+03,1.74E+03,1.89E+03
6E+01,2.75E+03,4.21E+02
0E+06,1.73E+06,6.69E+05
1E+05,2.73E+05,5.35E+05
1E+03,3.26E+03,3.14E+03
0E+04,8.42E+03,1.06E+04
7E+03,8.80E+03,3.60E+03
5E+03,1.92E+03,4.42E+02
E+02,2.07E+02,5.10E+01
4E+04,7.18E+04,2.46E+04
5E+03,8.09E+03,3.39E+03
1E+03,3.25E+03,3.64E+03
E+02,1.98E+02,1.62E+02
1E+04,1.54E+04,2.02E+04
2E+04,7.02E+03,1.16E+04
.56E+04,3.70E+04,4.94E+04
.80E+03,2.57E+03,2.10E+03
.52E+03,4.56E+01,2.80E+03
.84E+02,2.13E+03,1.59E+03
.48E+04,2.40E+04,2.39E+04
.69E+05,2.32E+06,1.07E+06
.96E+03,3.25E+03,3.91E+03
.66E+04,7.77E+04,3.25E+04

.62E+04,8.54E+04,3.62E+04
.3E+03,2.30E+04,9.54E+03
5E+04,9.45E+03,2.02E+03
.93E+03,1.45E+04,5.53E+03
.78E+03,4.66E+03,2.55E+01
.89E+04,5.27E+04,7.30E+04
.19E+03,3.17E+03,1.08E+03
.40E+03,2.45E+03,3.58E+03
.84E+03,3.23E+03,1.82E+03
.96E+03,6.82E+03,2.56E+03
.49E+03,3.11E+03,1.62E+03
.26E+03,1.27E+03,1.91E+03
.10E+03,2.08E+03,1.36E+03
.26E+03,4.64E+03,1.92E+03
3E+03,2.93E+03,2.75E+02
.67E+02,3.25E+02,6.71E+02
.35E+04,1.42E+04,1.82E+04
.07E+04,5.47E+04,9.02E+04
.07E+03,1.98E+04,4.67E+03
.71E+03,2.17E+03,2.31E+03
.45E+03,3.81E+03,4.84E+02
.88E+03,2.79E+03,2.99E+03
.80E+02,1.78E+03,3.92E+02
1E+02,6.04E+02,8.05E+01
.11E+03,2.14E+03,1.92E+03
.03E+04,7.28E+03,5.68E+03
E+04,1.91E+04,2.46E+04
.77E+04,1.39E+04,9.21E+04
.98E+04,3.03E+04,1.82E+04
.85E+01,3.51E+03,1.92E+03
.65E+04,6.54E+03,2.72E+04
.52E+02,1.32E+03,7.86E+02
0E+03,1.89E+03,2.79E+03
.74E+03,3.30E+03,5.03E+03
.83E+03,1.50E+03,1.18E+03
.56E+01,1.39E+03,1.60E+03
.96E+05,1.67E+05,1.25E+05
.47E+03,8.42E+02,5.18E+03
1E+03,3.79E+03,6.09E+03
.84E+03,3.18E+03,4.07E+03
.45E+03,6.09E+03,4.57E+03
.05E+03,2.53E+03,1.79E+03
.25E+05,1.73E+05,1.05E+05
.30E+01,9.52E+01,1.20E+02

.26E+05,8.18E+04,1.91E+05
.56E+03,1.40E+03,1.39E+02
.78E+02,1.65E+03,1.39E+02
.86E+02,1.79E+02,3.27E+02
.17E+03,1.60E+03,1.69E+03
9E+03,2.62E+03,9.56E+02
.97E+03,2.05E+03,4.87E+03
7E+04,1.37E+04,1.03E+04
9E+05,1.65E+05,9.63E+04
.93E+03,3.71E+03,1.18E+03
.28E+01,2.30E+02,2.41E+02
.22E+04,1.44E+04,1.23E+04
.02E+04,5.66E+04,4.69E+04
.53E+03,2.44E+03,1.32E+03
.82E+03,3.63E+03,1.65E+03
3E+02,5.32E+02,3.53E+02
.37E+03,2.87E+03,2.32E+03
.46E+04,4.85E+04,2.63E+04
.64E+03,2.50E+03,2.04E+03
.18E+03,3.11E+03,4.07E+03
.15E+03,2.22E+02,1.23E+03
.37E+03,2.03E+03,2.01E+03
.14E+03,5.44E+03,1.46E+03
.34E+03,2.23E+03,3.09E+02
.24E+03,4.98E+03,2.39E+03
.08E+03,3.84E+02,3.52E+02
.33E+03,2.08E+03,9.67E+02
9E+03,9.92E+02,6.78E+02
8E+03,1.05E+03,1.01E+03
.26E+03,2.37E+03,9.75E+01
.35E+03,1.37E+03,1.44E+03
.27E+02,2.15E+03,3.73E+02
.41E+02,1.50E+02,8.90E+01
.06E+03,2.52E+03,2.21E+03
.48E+03,1.68E+03,2.30E+03
.96E+03,1.90E+03,1.27E+03
.38E+03,1.89E+02,2.00E+01
.36E+03,2.33E+03,1.75E+03
.90E+02,3.46E+02,1.04E+03
.78E+03,3.48E+03,3.72E+03
.61E+03,3.80E+03,1.90E+03
.51E+02,1.60E+03,8.84E+01
.31E+04,7.09E+03,9.43E+03
8E+03,4.05E+03,7.92E+03

.79E+03,2.72E+03,7.25E+02
.4E+04,3.98E+04,5.02E+04
.12E+03,2.53E+03,1.96E+03
.30E+02,9.39E+02,5.12E+02
.70E+03,3.25E+03,7.99E+02
.08E+04,2.85E+04,3.04E+04
.76E+03,1.47E+03,1.70E+03
.77E+04,8.95E+03,2.38E+04
.16E+03,1.93E+03,3.02E+03
.64E+03,1.88E+03,1.47E+03
.88E+03,2.44E+03,3.46E+03
.10E+04,4.42E+03,1.71E+04
.07E+02,9.80E+01,3.33E+02
.61E+03,1.16E+04,1.35E+01
6E+02,1.32E+02,1.94E+02
.07E+04,1.75E+04,1.08E+04
5E+05,1.17E+05,5.06E+05
.50E+00,6.05E+01,1.75E+02
0E+05,2.05E+05,2.37E+05
.5E+04,3.76E+04,1.37E+05
.86E+04,4.46E+03,2.15E+04
.89E+03,1.56E+03,1.23E+03
E+02,1.44E+02,1.86E+03
.79E+06,1.10E+06,7.45E+05
.88E+01,5.57E+02,8.86E+03
.05E+03,5.49E+03,1.07E+04
.84E+03,3.45E+03,2.27E+03
.05E+04,4.74E+04,2.82E+04
.49E+03,3.15E+03,1.63E+03
.90E+03,3.29E+03,3.03E+03
.90E+03,5.03E+03,3.16E+03
.21E+02,3.34E+02,1.11E+02
.62E+04,3.33E+04,4.03E+03
.50E+01,3.70E+03,9.10E+02
.24E+04,2.14E+04,6.98E+03
.45E+04,8.49E+03,5.36E+03
.23E+04,3.38E+04,2.84E+04
.93E+03,7.79E+02,9.95E+02
.88E+03,2.27E+03,1.42E+03
.12E+04,2.31E+04,6.90E+03
.10E+01,2.27E+03,1.15E+02
.31E+03,1.25E+04,4.02E+03
.53E+03,1.57E+03,4.46E+01
.02E+03,1.64E+03,1.49E+03

.21E+03,1.74E+03,7.42E+02
.42E+03,1.81E+03,8.27E+02
4E+02,2.98E+02,1.87E+02
.01E+03,1.47E+03,4.00E+00
.29E+03,1.95E+03,4.00E+00
.57E+03,9.78E+03,3.66E+03
.3E+03,9.52E+02,1.20E+03
.77E+03,2.22E+03,3.50E+02
.07E+03,1.71E+03,1.31E+03
.47E+03,1.29E+03,9.60E+02
.11E+03,7.25E+03,2.66E+03
.01E+03,1.74E+03,1.97E+03
.32E+02,1.89E+02,6.50E+01
.29E+02,2.22E+03,1.90E+01
.62E+04,2.50E+04,1.64E+04
.73E+02,1.94E+01,1.24E+03
.3E+03,1.68E+03,1.96E+03
.26E+03,4.41E+03,2.60E+03
.23E+02,4.79E+02,1.45E+03
.97E+03,7.66E+03,1.66E+03
.15E+03,7.98E+03,3.33E+03
.89E+02,1.20E+03,1.23E+03
.87E+03,2.12E+03,1.59E+03
.19E+03,1.61E+03,8.93E+02
.88E+01,1.41E+03,7.16E+02
.15E+03,5.76E+03,2.77E+03
.50E+02,2.84E+03,6.82E+03
.23E+03,2.07E+03,2.33E+03
.62E+03,5.10E+03,1.18E+03
.86E+01,2.94E+03,1.00E+00
.97E+03,4.36E+03,9.51E+03
1E+03,1.22E+04,1.14E+04
1E+03,4.00E+03,4.72E+03
6E+03,2.92E+03,3.17E+03
.59E+03,4.52E+03,3.57E+03
.80E+03,2.36E+03,7.33E+02
.40E+03,1.42E+03,1.70E+03
.39E+02,5.95E+02,1.08E+02
.55E+03,5.87E+02,1.48E+03
.72E+03,4.19E+03,2.96E+03
.99E+04,1.21E+04,3.05E+04
.68E+03,2.25E+03,1.49E+03
.03E+04,5.79E+03,7.71E+03
.10E+04,6.18E+03,6.44E+03

.25E+03,1.41E+03,1.33E+03
.12E+03,1.89E+03,1.86E+03
.04E+03,1.13E+04,2.04E+03
.65E+05,5.13E+05,5.81E+05
.07E+03,1.56E+04,3.42E+03
.76E+05,4.57E+05,1.82E+05
.82E+04,1.98E+04,2.93E+04
02E+03,1.77E+03,5.10E+03
.93E+04,1.71E+04,7.12E+03
.26E+03,2.92E+03,2.96E+03
.04E+03,5.17E+03,1.57E+03
.93E+03,5.54E+01,2.70E+02
2E+04,5.60E+05,1.13E+05
.43E+03,1.23E+03,2.84E+03
.01E+03,3.12E+03,5.33E+02
.43E+03,1.98E+04,3.27E+03
.02E+02,1.32E+03,2.11E+03
.22E+03,2.95E+03,1.47E+02
.01E+02,2.04E+03,7.00E+01
.19E+03,6.59E+02,8.84E+02
.75E+02,3.70E+03,4.52E+02
.29E+03,1.47E+03,1.53E+02
.49E+03,1.30E+03,9.06E+02
.04E+03,2.57E+03,7.23E+02
.36E+03,1.29E+03,1.62E+03
.15E+03,1.98E+03,1.73E+03
.19E+03,1.90E+03,1.59E+03
.32E+02,1.69E+03,1.17E+03
.11E+02,1.37E+03,8.20E+01
.82E+02,2.50E+02,8.20E+01
.44E+03,2.05E+03,8.20E+01
.34E+03,2.77E+03,1.96E+03
.68E+03,1.81E+03,2.21E+02
.22E+03,8.84E+02,8.44E+02
.27E+03,2.45E+03,1.98E+03
.95E+03,1.34E+03,1.46E+03
.06E+03,2.39E+03,1.19E+03
.99E+03,2.31E+03,1.85E+03
.06E+03,2.26E+03,5.52E+02
.05E+03,1.78E+03,2.95E+03
.72E+03,2.13E+03,9.38E+02
3E+03,2.23E+03,1.00E+00
.19E+03,1.11E+02,3.41E+03
.35E+05,1.15E+05,4.14E+04

.60E+03,1.40E+03,1.15E+03
.32E+04,2.60E+04,3.21E+04
.31E+03,1.77E+03,6.64E+00
.45E+01,3.16E+03,6.64E+00
5E+01,8.86E+02,1.73E+03
5E+01,1.31E+03,5.46E+02
.26E+03,5.74E+03,2.39E+03
.06E+03,2.21E+03,1.68E+03
.12E+03,2.55E+03,1.01E+03
.24E+03,3.17E+03,2.98E+03
3E+02,1.40E+00,1.83E+03
.73E+03,5.42E+03,6.61E+03
.38E+03,7.10E+00,2.42E+03
.68E+02,3.57E+03,1.89E+01
.68E+02,1.91E+03,1.89E+01
.30E+04,1.60E+04,6.05E+04
.25E+03,1.11E+04,4.14E+03
.44E+01,1.95E+02,2.59E+01
.49E+03,3.60E+03,1.26E+03
.46E+04,6.65E+04,8.18E+04
8E+03,1.96E+01,2.20E+03
.45E+03,1.95E+03,1.21E+04
.78E+03,2.92E+03,2.99E+03
.06E+03,1.00E+04,3.97E+03
.18E+03,4.50E+04,6.18E+03
.76E+02,2.60E+03,1.52E+02
.22E+02,2.02E+03,5.96E+02
.21E+05,5.80E+05,4.67E+05
.36E+03,3.37E+03,9.32E+03
.44E+03,2.20E+03,1.90E+01
.07E+04,3.91E+04,3.12E+04
.04E+03,3.26E+03,5.04E+03
.31E+03,3.61E+03,1.52E+03
.26E+03,2.78E+03,4.67E+01
.16E+03,2.28E+03,1.38E+03
.54E+01,2.40E+03,1.99E+01
5E+04,4.22E+04,3.46E+04
.13E+03,1.95E+03,1.49E+03
.24E+03,8.26E+02,9.10E+02
.91E+03,4.50E+03,3.36E+03
.35E+02,2.90E+01,2.00E+01
.35E+02,2.90E+01,2.00E+01
.03E+03,1.61E+03,1.32E+03
.14E+03,6.17E+03,3.27E+03

78E+01,1.69E+03,9.80E+00
6E+03,1.40E+03,1.49E+03
02E+03,1.44E+03,5.79E+02
18E+03,4.92E+03,4.61E+03
95E+03,1.48E+02,2.08E+03
68E+03,1.11E+03,1.28E+03
46E+03,1.83E+03,1.44E+03
45E+03,2.57E+02,1.15E+03
99E+04,9.84E+04,7.58E+03
35E+03,2.91E+03,1.94E+03
95E+03,3.45E+03,2.16E+03
18E+03,3.37E+03,1.40E+03
4E+03,1.63E+03,1.39E+03
44E+03,1.91E+03,1.56E+03
47E+03,1.40E+03,1.53E+03
22E+05,1.59E+05,1.03E+04
40E+03,5.06E+03,1.23E+03
93E+03,2.18E+03,1.15E+03
82E+03,6.83E+03,2.23E+03
0E+03,2.09E+03,6.38E+01
43E+03,3.43E+03,4.58E+03
66E+04,2.71E+04,2.81E+04
09E+03,3.47E+03,5.04E+03
06E+03,2.13E+03,1.75E+03
41E+03,5.49E+03,1.29E+03
75E+03,1.83E+03,2.60E+03
19E+03,7.61E+02,7.76E+02
70E+04,8.22E+04,6.99E+03
60E+03,1.72E+03,3.52E+01
11E+03,1.97E+03,5.32E+02
61E+04,1.76E+04,2.38E+04
16E+03,3.95E+03,5.46E+03
19E+03,7.28E+02,2.07E+03
E+03,3.27E+01,3.93E+02
03E+03,5.44E+03,9.75E+02
98E+03,1.53E+03,1.30E+03
71E+04,2.13E+04,2.28E+03
13E+03,8.37E+03,2.95E+03
08E+03,2.88E+03,6.35E+02
37E+04,2.17E+04,1.39E+05
50E+04,1.57E+04,2.97E+04
34E+03,2.05E+03,2.85E+02
14E+03,3.27E+03,2.22E+03
59E+02,8.00E+00,1.58E+03

.48E+03,1.93E+03,3.22E+03
.35E+03,1.71E+03,1.39E+03
.24E+03,7.84E+03,1.10E+03
0E+03,1.48E+03,3.15E+03
.99E+03,4.97E+03,9.28E+02
.63E+03,1.83E+03,1.12E+03
.04E+03,2.96E+03,3.44E+03
.85E+03,1.84E+03,2.21E+03
.82E+01,2.96E+03,1.03E+03
.44E+02,2.74E+03,9.70E+01
.44E+02,1.22E+03,9.70E+01
.09E+03,3.68E+03,4.39E+03
.61E+04,1.00E+05,7.15E+04
.40E+03,3.66E+03,1.05E+03
.74E+03,1.57E+03,7.02E+02
.91E+03,2.72E+03,2.23E+03
0E+00,1.15E+03,5.67E+01
.17E+03,1.75E+03,6.65E+01
.20E+00,1.75E+03,1.12E+03
.36E+03,2.58E+03,1.29E+03
.06E+03,1.69E+03,2.35E+03
.78E+02,1.33E+03,9.30E+02
.91E+03,2.76E+03,7.70E+02
.04E+03,1.31E+03,9.46E+02
.55E+03,2.26E+03,1.08E+03
.18E+02,2.12E+03,6.07E+02
.54E+03,1.64E+03,1.32E+03
.38E+03,1.37E+03,8.75E+02
.41E+04,1.62E+04,2.22E+04
.89E+03,1.30E+03,8.75E+02
.00E+03,1.90E+03,1.78E+03
.55E+03,1.25E+03,1.31E+03
.07E+03,1.26E+02,1.46E+01
.17E+03,1.57E+03,1.20E+03
0E+03,1.51E+03,1.72E+03
.50E+03,1.47E+03,1.49E+03
.37E+03,5.10E+03,1.32E+03
.68E+02,1.42E+03,1.80E+01
5E+03,3.16E+03,1.13E+04
.44E+02,9.57E+02,6.27E+02
.35E+03,1.87E+03,1.55E+03
.58E+02,3.95E+03,6.54E+02
.58E+02,3.93E+02,1.27E+03
.96E+03,1.55E+03,3.18E+03

91E+03,1.57E+03,1.49E+02
93E+05,3.50E+05,4.01E+05
89E+02,1.12E+03,8.14E+02
20E+04,1.07E+04,1.25E+04
80E+03,3.41E+03,2.45E+03
53E+03,1.79E+03,1.22E+03
3E+04,7.50E+04,4.78E+04
91E+04,8.74E+04,2.30E+04
09E+03,3.69E+03,2.99E+03
18E+03,3.24E+03,1.60E+03
31E+03,1.95E+03,3.13E+02
00E+01,2.40E+03,1.13E+03
88E+03,2.62E+01,3.45E+03
2E+04,3.13E+04,1.96E+04
83E+03,1.82E+03,1.26E+03
0E+02,9.70E+01,2.46E+01
29E+04,4.97E+04,2.04E+03
35E+02,1.90E+03,1.19E+03
78E+03,1.05E+03,7.86E+02
36E+03,1.24E+03,1.07E+03
0E+04,6.89E+04,3.03E+03
4E+03,2.03E+03,1.31E+03
75E+03,2.67E+03,2.38E+02
70E+01,6.72E+01,1.40E+02
87E+03,1.83E+03,2.13E+03
29E+03,2.24E+03,5.74E+02
25E+03,1.04E+03,7.43E+02
89E+03,1.69E+04,1.33E+03
60E+03,1.17E+04,1.39E+03
1E+02,2.11E+03,8.10E+01
81E+03,7.42E+03,2.11E+03
56E+03,1.85E+03,1.96E+03
1E+03,1.83E+03,1.91E+03
50E+03,7.54E+03,5.46E+02
95E+03,1.13E+03,5.62E+02
92E+03,2.78E+03,1.94E+03
81E+03,5.77E+03,1.93E+03
50E+02,4.10E+01,1.96E+03
47E+03,1.05E+03,1.41E+03
09E+03,1.17E+02,4.16E+02
27E+03,1.84E+03,2.14E+03
02E+02,2.04E+03,2.77E+02
88E+02,2.00E+03,8.30E+02
72E+02,2.63E+03,4.20E+02

.68E+03,3.22E+03,1.33E+03
.05E+03,6.48E+01,7.04E+02
.56E+03,1.26E+03,1.30E+03
.11E+03,4.79E+03,3.78E+03
.83E+02,7.39E+02,7.96E+02
.12E+02,2.00E+01,1.52E+03
.12E+02,1.48E+03,1.54E+03
8E+03,2.49E+03,3.69E+03
1E+03,1.56E+03,2.42E+03
.18E+03,1.65E+03,1.17E+03
.35E+03,3.68E+03,6.86E+02
.78E+02,1.50E+03,7.40E+01
.35E+04,1.62E+04,7.67E+03
.31E+03,4.09E+03,4.13E+03
.53E+05,1.49E+05,1.03E+05
.74E+03,1.82E+03,2.10E+03
.49E+04,1.33E+04,1.42E+04
.49E+04,2.39E+04,4.48E+04
.60E+03,2.29E+03,2.70E+03
.25E+03,6.88E+03,4.70E+03
.53E+03,1.43E+03,1.39E+03
.32E+03,5.83E+03,4.62E+03
.25E+03,4.44E+03,5.56E-01
.69E+03,2.04E+03,1.37E+03
.73E+03,2.13E+02,5.50E+00
.91E+03,1.77E+03,1.58E+03
.26E+03,1.27E+03,8.63E+02
.84E+03,3.30E+03,3.67E+03
.48E+03,1.39E+03,1.10E+03
.06E+03,3.99E+03,9.83E+01
.12E+03,2.03E+03,1.27E+03
.93E+03,6.05E+03,3.08E+03
.00E-01,5.00E-01,3.16E+02
.33E+03,3.83E+03,2.74E+03
.76E+04,2.18E+04,9.45E+03
.78E+03,1.84E+03,7.80E+00
.75E+03,9.90E+02,1.39E+03
.98E+03,1.91E+03,1.73E+03
.52E+03,1.81E+03,2.52E+03
.25E+02,8.95E+02,2.30E+02
.69E+03,2.50E+03,1.11E+03
.74E+02,1.18E+03,2.30E+02
.52E+03,1.66E+03,4.86E+02
.26E+03,2.77E+03,7.98E+02

.23E+05,8.62E+04,9.03E+04
.34E+03,5.31E+03,5.71E+03
.76E+03,1.43E+03,1.24E+03
8E+03,1.59E+03,5.85E+01
.46E+03,2.34E+03,3.31E+03
.82E+03,7.65E+02,7.41E+02
.25E+04,1.60E+04,1.45E+04
.03E+01,1.62E+03,1.13E+03
.13E+03,1.71E+03,1.69E+03
.93E+03,2.02E+03,4.03E+02
7E+03,1.54E+03,3.14E+02
.26E+03,1.88E+03,1.27E+03
.22E+03,1.83E+03,1.59E+03
.35E+03,5.22E+02,1.22E+03
.92E+03,1.72E+03,1.43E+02
.56E+03,1.51E+03,1.70E+03
.51E+03,2.48E+03,1.64E+03
.64E+02,5.13E+03,2.20E+01
.51E+03,1.54E+03,1.11E+02
.09E+03,2.14E+03,1.81E+03
.27E+04,1.27E+04,1.02E+04
.22E+03,2.53E+01,2.95E+03
.18E+02,1.49E+03,1.29E+03
.61E+03,2.47E+03,6.00E+02
.07E+03,2.12E+03,2.41E+03
.38E+03,1.91E+03,9.78E+02
.39E+03,3.41E+03,5.86E+02
.48E+02,1.56E+03,3.15E+02
.38E+02,1.49E+03,5.32E+02
.48E+03,1.93E+03,5.34E+02
.79E+03,2.45E+03,2.77E+03
.34E+02,1.77E+03,6.09E+02
7E+03,2.01E+03,1.05E+03
.26E+03,1.52E+03,6.17E+02
.54E+03,2.07E+03,1.69E+03
.39E+03,4.82E+03,4.96E+03
.82E+03,2.35E+03,1.75E+03
.22E+03,5.53E+03,1.09E+04
.56E+03,2.46E+03,1.27E+03
.45E+01,1.98E+03,6.08E+02
.65E+03,2.45E+03,1.18E+02
.44E+03,1.22E+03,7.21E+02
.29E+03,1.46E+03,1.61E+03
.72E+03,3.32E+02,1.48E+03

.79E+03,1.56E+03,1.10E+03
.27E+03,1.29E+03,8.98E+02
.79E+03,6.74E+03,2.38E+03
.95E+03,1.97E+03,1.65E+03
1E+03,3.09E+03,1.87E+03
.17E+04,2.94E+03,1.60E+04
.04E+04,3.14E+04,1.40E+04
.34E+03,1.32E+03,1.47E+03
.73E+03,1.62E+03,2.15E+03
.13E+03,1.34E+03,1.53E+03
.49E+01,1.37E+03,8.08E+02
.96E+03,9.51E+02,8.30E+02
3E+03,1.74E+03,1.51E+03
.44E+03,2.34E+03,6.30E+01
.11E+03,1.07E+03,1.48E+03
.64E+03,2.03E+03,1.31E+03
.62E+02,1.85E+03,3.08E+02
.98E+03,7.71E+03,3.67E+03
.07E+01,9.58E+01,2.40E+01
.49E+03,1.73E+03,2.33E+03
.53E+03,1.77E+03,9.91E+02
.78E+03,1.62E+03,1.29E+03
.07E+03,1.64E+03,1.26E+03
.30E+03,1.37E+03,1.24E+03
.07E+02,1.82E+03,5.07E+02
.13E+03,2.27E+03,1.14E+03
.96E+03,2.90E+03,1.52E+03
.26E+03,7.47E+03,3.50E+02
.42E+02,4.52E+01,1.85E+02
.56E+03,1.69E+03,5.15E+02
.04E+03,9.28E+02,3.24E+02
.94E+03,2.14E+03,1.20E+03
.03E+03,4.33E+03,4.57E+02
.53E+03,1.29E+03,9.70E+01
.37E+03,1.69E+03,1.26E+03
.61E+03,2.28E+03,1.45E+03
.19E+03,2.75E+03,1.26E+03
.10E+01,2.24E+03,6.30E+01
.38E+04,1.25E+04,6.92E+03
.21E+03,5.71E+02,1.89E+03
.90E+02,1.25E+03,1.15E+03
.03E+03,1.47E+03,1.93E+03
.42E+03,2.57E+03,2.40E+03
.39E+03,6.50E+03,3.97E+03

.08E+03,2.85E+03,9.96E+02
5E+01,1.75E+03,1.60E+01
.30E+05,5.57E+05,6.59E+05
.78E+02,5.46E+02,3.61E+02
.20E+04,3.20E+04,4.54E+04
6E+03,5.08E+03,1.92E+03
.85E+01,1.70E+03,4.79E+02
.46E+03,2.30E+03,1.61E+03
.90E+04,2.12E+04,1.23E+04
.79E+03,2.32E+03,3.03E+03
.99E+01,1.54E+03,4.87E+02
.73E+03,1.94E+03,2.49E+03
.74E+03,1.14E+03,1.77E+03
.44E+03,2.39E+03,1.29E+03
.75E+03,1.62E+03,2.30E+03
.28E+04,2.47E+04,1.59E+04
.51E+02,1.56E+00,6.92E+02
.20E+04,5.66E+03,4.40E+03
.52E+03,1.93E+03,7.70E+02
.63E+03,1.54E+03,1.36E+03
0E+03,1.77E+03,1.29E+03
.01E+03,2.70E+03,2.60E+03
.36E+03,1.91E+03,2.10E+03
.97E+03,2.29E+03,1.67E+03
.78E+03,1.36E+03,5.83E+01
E+03,1.77E+02,2.31E+03
.52E+03,1.55E+03,5.40E+01
.75E+03,2.28E+03,1.63E+03
.43E+04,8.84E+03,1.94E+04
.29E+03,2.52E+03,2.57E+03
.25E+03,3.79E+01,1.71E+03
.58E+03,2.04E+03,1.36E+03
.42E+03,9.08E+02,2.27E+03
.61E+03,2.10E+03,1.17E+03
.18E+01,2.51E+02,5.04E+02
7E+03,2.31E+03,2.86E+02
.59E+02,2.20E+03,2.01E+02
.47E+03,6.61E+03,2.41E+03
.51E+03,2.44E+03,1.78E+03
.78E+03,1.62E+03,1.13E+03
.26E+02,2.54E+03,2.07E+03
.24E+03,5.79E+02,5.33E+02
.61E+02,1.89E+02,1.48E+03
.49E+03,1.07E+03,1.78E+03

14E+02,1.75E+03,1.22E+02
38E+03,1.25E+03,9.95E+02
40E+02,1.45E+03,1.64E+03
28E+04,6.84E+04,2.94E+04
47E+03,1.01E+03,1.18E+03
22E+03,1.45E+02,3.44E+02
7E+03,8.87E+02,8.78E+02
42E+03,4.03E+03,2.81E+03
70E+03,3.24E+03,1.54E+03
39E+02,4.13E+02,3.21E+02
44E+03,7.94E+03,4.67E+03
50E+03,2.09E+03,9.51E+02
40E+03,3.79E+03,4.50E+01
96E+04,2.86E+04,1.36E+04
20E+03,1.23E+03,1.35E+03
05E+03,1.26E+03,1.39E+03
20E+03,1.42E+03,1.36E+03
25E+03,1.29E+03,1.13E+03
76E+03,1.67E+03,2.20E+03
81E+04,2.62E+04,2.52E+04
32E+03,1.80E+03,1.75E+03
18E+03,3.38E+03,1.55E+03
44E+02,4.19E+02,1.39E+01
87E+03,5.26E+03,4.10E+03
48E+03,3.29E+03,3.21E+03
68E+03,1.54E+03,1.31E+03
20E+02,5.95E+02,9.50E+02
36E+04,1.70E+04,7.26E+03
92E+02,8.40E+01,1.13E+03
63E+03,1.79E+03,6.89E+02
23E+03,2.13E+03,1.71E+03
14E+03,1.92E+03,2.22E+03
55E+03,8.60E+01,5.24E+02
10E+03,1.87E+03,1.77E+03
2E+03,1.62E+03,2.03E+02
20E+03,1.96E+03,1.01E+03
92E+03,2.85E+03,1.06E+03
11E+04,1.79E+04,4.84E+03
00E+01,1.91E+03,2.48E+03
13E+03,7.41E+02,5.55E+01
39E+03,3.46E+03,1.63E+03
35E+02,1.45E+03,1.88E+03
83E+04,3.27E+04,3.98E+04
25E+03,1.71E+03,2.05E+03

.45E+02,1.26E+03,2.42E+02
.19E+03,2.58E+03,2.37E+03
.78E+03,1.96E+03,1.26E+03
.75E+03,2.30E+03,6.19E+01
.20E+03,2.12E+03,1.68E+03
.67E+03,2.54E+03,1.65E+03
.33E+03,1.87E+03,4.65E+03
.54E+03,1.71E+03,9.93E+01
.80E+01,1.58E+03,9.93E+01
.57E+03,1.46E+03,1.73E+03
.98E+02,2.06E+03,4.55E+01
.73E+02,4.65E+02,1.19E+03
5E+02,1.76E+03,1.11E+03
.84E+02,1.37E+03,9.56E+02
.67E+03,1.48E+03,2.12E+03
.21E+03,1.48E+03,1.02E+03
.36E+03,2.51E+03,3.77E+03
.77E+03,1.28E+03,2.81E+02
.91E+02,2.76E+02,7.60E+01
.15E+03,2.76E+02,7.60E+01
.50E+03,2.02E+03,3.38E+03
.71E+03,2.04E+02,1.39E+03
.42E+03,4.15E+03,3.86E+03
.21E+03,8.61E+02,9.42E+02
.45E+04,1.78E+04,7.72E+03
.19E+03,1.50E+03,3.32E+02
.99E+02,2.22E+03,1.68E+03
.09E+03,2.00E+03,7.85E+01
.54E+03,1.66E+03,1.08E+03
.94E+03,1.51E+03,8.24E+02
.52E+03,1.48E+03,1.14E+03
.54E+03,7.12E+02,1.00E+03
.99E+02,1.38E+03,5.14E+02
.92E+03,2.11E+03,2.25E+03
.04E+03,1.49E+03,1.66E+03
.27E+03,1.35E+03,2.23E+03
.71E+03,1.94E+03,1.28E+03
.58E+03,6.05E+02,9.63E+02
6E+03,1.58E+03,1.11E+03
.67E+02,1.83E+03,1.42E+03
.51E+02,1.67E+03,1.30E+03
.54E+03,1.97E+03,1.62E+03
.79E+03,2.16E+03,9.85E+02
.30E+02,1.63E+03,1.86E+02

29E+03,1.70E+03,2.06E+03
61E+01,1.78E+03,8.00E+00
57E+03,1.25E+03,1.17E+03
90E+01,1.33E+03,1.11E+03
75E+03,1.78E+03,2.21E+03
01E+03,2.78E+03,1.68E+03
60E+03,1.73E+03,1.12E+03
63E+02,5.50E+02,1.31E+03
50E+03,1.90E+03,2.12E+03
42E+03,1.40E+03,1.36E+03
31E+03,5.70E+03,1.51E+04
17E+02,1.07E+03,1.18E+02
34E+03,6.75E+03,3.12E+03
74E+03,1.32E+03,2.01E+03
81E+03,1.71E+03,7.64E+02
20E+01,1.37E+03,7.43E+02
59E+05,1.84E+05,2.04E+05
80E+01,1.92E+03,1.51E+02
80E+01,1.02E+02,1.51E+02
72E+03,2.80E+03,1.64E+03
80E+04,1.15E+04,1.14E+04
42E+02,1.07E+02,5.66E+02
15E+02,5.95E+02,1.60E+03
06E+03,1.57E+03,8.23E+01
01E+03,1.89E+03,1.44E+03
69E+03,2.25E+03,1.67E+03
23E+03,1.71E+03,2.47E+03
92E+02,1.08E+03,7.41E+01
73E+02,9.00E+01,3.90E+02
10E+01,6.15E+03,4.30E+01
10E+01,2.00E+03,1.72E+03
10E+03,1.74E+03,4.47E+02
36E+03,1.52E+03,9.11E+02
84E+03,1.69E+03,1.23E+03
52E+04,1.23E+04,1.40E+04
82E+03,1.91E+03,1.44E+03
44E+03,1.77E+03,2.01E+02
05E+03,3.31E+03,3.07E+03
42E+02,1.33E+02,1.17E+02
3E+03,1.08E+03,1.38E+03
47E+03,1.28E+03,1.26E+03
89E+03,6.36E+02,1.54E+03
96E+03,6.40E+01,1.56E+03
24E+01,1.68E+03,1.00E+02

.60E+03,1.80E+03,1.41E+03
.26E+03,2.19E+03,1.52E+03
.67E+03,1.74E+03,1.39E+03
.42E+02,2.50E+02,1.92E+01
.17E+03,7.00E+02,1.43E+03
.29E+03,1.75E+03,2.33E+03
6E+03,4.48E+03,1.82E+03
.31E+03,2.88E+02,6.68E+01
.08E+03,2.22E+03,1.49E+03
.14E+03,2.26E+03,1.31E+03
.78E+03,2.58E+03,2.50E+03
.03E+03,2.02E+03,7.15E+02
.04E+03,1.84E+03,1.22E+03
.06E+03,1.93E+03,1.84E+03
.10E+01,4.87E+02,7.26E+02
.74E+03,1.60E+02,8.62E+02
.83E+03,2.17E+03,2.73E+03
.41E+03,3.02E+03,1.90E+03
.02E+04,6.12E+04,1.33E+05
.02E+02,6.49E+02,4.75E+00
.65E+05,5.28E+05,1.17E+05
.65E+03,9.35E+02,1.08E+03
.76E+03,1.56E+04,1.77E+04
.01E+04,2.90E+04,1.27E+04
.49E+04,1.99E+04,2.91E+04
.42E+03,1.75E+03,1.55E+03
6E+03,1.67E+03,2.50E+03
.41E+03,2.19E+03,2.91E+03
.18E+03,1.67E+03,8.12E+02
.16E+02,1.26E+03,2.72E+03
.66E+03,9.94E+02,1.25E+03
.13E+03,1.97E+03,2.02E+03
.27E+03,1.15E+03,5.66E+02
.28E+03,1.98E+03,2.01E+03
.31E+03,1.22E+02,1.36E+02
.91E+03,1.22E+02,1.25E+03
.77E+03,2.27E+03,7.79E+03
.66E+01,1.25E+03,2.26E+03
.49E+03,1.95E+03,7.98E+02
.24E+03,1.73E+02,1.31E+03
.68E+03,1.95E+03,1.66E+03
.70E+03,1.81E+03,1.60E+03
2E+03,1.36E+03,8.90E+02
.02E+03,2.08E+03,1.62E+03

.51E+01,1.68E+03,1.27E+03
.20E+02,6.35E+02,1.17E+03
0E+02,2.24E+02,3.47E+02
.24E+03,1.59E+03,1.49E+03
.43E+03,1.77E+03,1.17E+03
1E+03,1.38E+03,6.92E+02
.31E+03,1.54E+03,2.08E+03
.55E+03,1.64E+03,1.07E+03
3E+03,1.90E+03,1.35E+03
.13E+03,6.43E+02,1.03E+03
.06E+02,2.06E+03,2.42E+03
.96E+02,1.46E+03,1.65E+03
.85E+03,3.26E+02,1.56E+03
.58E+03,1.87E+03,2.69E+02
.80E+03,2.27E+03,2.69E+03
.32E+02,1.59E+03,1.33E+03
.32E+02,1.95E+03,1.11E+03
.54E+03,9.19E+02,1.45E+03
.91E+03,2.28E+03,1.21E+02
.10E+03,1.25E+03,1.44E+03
.35E+03,6.72E+03,4.27E+03
.28E+03,7.27E+03,5.27E+03
.79E+03,1.91E+03,1.00E+02
.38E+03,1.70E+02,1.21E+03
.70E+03,2.77E+03,4.41E+03
.30E+01,1.11E+03,1.18E+03
.30E+01,4.40E+01,9.18E+02
.30E+01,4.40E+01,1.33E+03
.10E+03,2.48E+03,1.53E+01
4E+03,1.66E+03,1.01E+03
.41E+03,1.90E+03,1.63E+03
.73E+03,1.62E+03,1.09E+04
.87E+03,1.40E+03,8.23E+02
.94E+03,3.86E+03,6.87E+03
.32E+03,1.41E+03,1.38E+03
.08E+02,1.11E+03,1.22E+03
.90E+02,1.50E+03,1.32E+03
.75E+03,1.72E+02,2.07E+02
.66E+03,1.38E+03,1.12E+02
.93E+02,6.01E+02,7.47E+02
.93E+02,1.35E+03,6.87E+02
.34E+03,1.37E+03,1.04E+03
.41E+03,1.23E+03,1.23E+03
E+01,1.26E+03,9.00E+00

58E+02,1.68E+03,1.50E+02
65E+03,1.77E+03,5.70E+01
58E+03,1.52E+03,1.74E+03
91E+03,6.36E+02,7.40E+02
09E+03,3.01E+03,1.51E+03
81E+03,1.72E+03,1.07E+03
52E+02,1.83E+03,1.55E+03
44E+03,1.42E+02,8.28E+01
3E+03,3.46E+03,1.12E+03
39E+04,3.55E+03,1.33E+04
96E+03,1.84E+03,1.04E+03
4E+03,2.04E+03,1.25E+02
63E+03,1.17E+03,1.36E+03
87E+03,5.63E+02,4.90E+02
58E+03,2.05E+03,1.37E+02
59E+04,1.38E+04,1.62E+04
06E+01,3.20E+01,4.89E+03
90E+03,6.75E+01,8.75E+02
36E+03,5.27E+03,3.47E+03
E+03,2.04E+02,2.17E+03
10E+03,2.04E+02,1.21E+03
97E+02,1.61E+03,2.70E+01
04E+03,5.61E+02,2.47E+02
45E+03,5.47E+03,1.88E+03
29E+03,2.75E+04,3.80E+04
51E+03,1.25E+03,5.47E+02
51E+03,2.66E+03,5.09E+03
3E+03,3.18E+03,4.85E+03
58E+03,1.21E+03,5.91E+02
97E+02,1.07E+03,1.20E+03
0E+03,8.47E+03,2.10E+04
11E+03,1.05E+03,1.03E+03
95E+01,1.82E+03,1.44E+02
E+03,2.65E+03,2.90E+03
50E+01,1.22E+03,3.78E+03
38E+03,2.93E+02,1.35E+03
89E+04,1.20E+05,3.92E+04
05E+03,1.43E+03,1.05E+03
37E+03,3.47E+03,8.09E+03
E+03,1.87E+03,2.85E+03
51E+02,2.69E+03,8.80E+01
4E+03,1.04E+04,4.49E+03
25E+03,1.97E+03,2.65E+03
48E+03,9.53E+02,1.80E+03

41E+03,1.63E+03,1.23E+03
29E+03,1.95E+02,1.96E+02
0E+03,1.70E+03,2.55E+02
17E+03,1.44E+03,7.72E+02
14E+03,1.73E+03,7.87E+02
35E+03,2.13E+03,2.46E+03
32E+03,1.31E+03,1.91E+03
09E+03,5.22E+02,2.25E+03
24E+03,6.08E+02,9.78E+02
E+03,2.57E+03,6.70E+01
73E+03,2.49E+03,3.63E+02
00E+03,1.34E+03,1.13E+03
41E+02,8.31E+02,1.40E+01
47E+03,1.62E+03,1.38E+03
39E+03,1.26E+03,1.12E+03
69E+02,7.80E+01,1.33E+02
73E+03,1.32E+03,1.23E+03
64E+03,1.54E+03,9.93E+02
37E+04,7.39E+03,1.37E+04
33E+04,7.79E+03,1.40E+04
06E+03,2.21E+03,1.18E+03
22E+03,2.10E+03,1.07E+03
84E+03,7.94E+01,1.55E+03
73E+03,1.81E+03,3.95E+01
12E+03,2.03E+03,1.60E+03
34E+03,1.52E+03,9.63E+00
76E+03,8.68E+02,6.14E+02
81E+02,1.79E+03,1.92E+03
5E+03,2.35E+03,2.76E+01
24E+02,9.05E+02,1.26E+03
46E+03,1.34E+03,6.02E+02
40E+01,1.65E+03,6.10E+02
39E+02,2.06E+03,4.95E+01
25E+03,2.50E+03,1.11E+03
20E+03,1.83E+03,6.21E+03
46E+03,1.67E+03,1.39E+03
14E+03,1.81E+03,1.45E+03
95E+02,1.90E+02,6.70E+01
90E+02,9.14E+02,4.74E+02
35E+04,4.72E+04,2.11E+04
11E+04,4.29E+04,1.81E+04
36E+03,1.09E+03,1.31E+03
77E+03,1.19E+03,7.09E+02
60E+03,2.11E+03,1.64E+03

.00E+03,2.06E+02,1.88E+03
.04E+03,1.62E+02,5.64E+02
.52E+03,4.80E+03,2.51E+03
0E+01,7.90E+02,4.42E+02
.88E+03,7.90E+02,4.54E+02
.65E+02,1.16E+03,7.66E+02
.70E+03,1.11E+03,5.60E+01
.60E+01,1.59E+03,1.36E+03
.81E+03,9.00E+02,1.55E+03
.64E+03,7.00E+02,3.01E+03
.12E+03,2.17E+03,1.37E+03
.28E+02,1.71E+02,1.24E+02
.26E+02,1.87E+03,1.29E+03
.09E+03,2.05E+03,1.08E+03
.1E+03,1.83E+03,8.84E+02
.99E+03,1.86E+03,8.74E+02
.72E+03,1.79E+03,3.27E+01
.57E+03,1.72E+03,1.08E+03
.27E+03,1.33E+03,1.44E+03
.59E+03,3.39E+03,1.72E+03
.71E+02,2.91E+03,5.45E+02
.26E+02,9.81E+02,7.38E+02
.26E+02,3.21E+02,2.00E+02
4E+03,1.76E+03,1.61E+03
.25E+02,1.31E+03,1.40E+03
.80E+02,6.85E+02,1.06E+03
.11E+03,2.07E+03,1.23E+03
.27E+03,1.56E+03,5.68E+02
.71E+03,1.39E+03,8.49E+02
.03E+03,1.67E+03,1.06E+03
.15E+03,2.36E+03,1.03E+04
.03E+03,3.08E+03,1.20E+04
.03E+02,2.85E+02,1.54E+03
.89E+03,1.22E+03,3.66E+02
.46E+03,1.30E+03,1.26E+03
.32E+03,5.59E+01,1.04E+03
.26E+02,2.37E+03,1.06E+03
.69E+03,1.21E+02,4.60E+03
.57E+02,1.67E+03,6.98E+02
.35E+03,2.22E+03,1.38E+03
.52E+03,2.46E+03,1.58E+03
6E+03,1.23E+03,1.50E+03
.19E+03,1.75E+03,7.28E+02
.70E+03,1.96E+03,1.52E+03

.33E+03,1.70E+03,1.17E+03
.97E+03,1.48E+03,1.66E+03
.37E+03,2.85E+03,9.64E+02
.56E+03,1.51E+03,1.46E+03
7E+03,1.95E+03,4.50E+03
.38E+03,2.38E+03,5.09E+03
.71E+01,1.53E+03,2.06E+03
.35E+03,2.05E+03,1.00E+03
.13E+03,1.73E+03,1.43E+03
.83E+03,3.75E+03,3.75E+03
.66E+03,3.85E+03,3.96E+03
.42E+03,1.29E+03,2.87E+03
.97E+03,8.87E+02,6.77E+02
.51E+03,2.10E+03,1.78E+03
18E+01,2.51E+02,4.65E+01
.87E+03,1.45E+03,1.81E+03
.97E+02,2.31E+02,1.39E+03
.74E+03,1.20E+03,2.31E+03
0E+01,5.56E+02,5.96E+02
.30E+01,5.56E+02,5.96E+02
.62E+03,1.64E+03,5.57E+02
.26E+01,6.65E+01,1.63E+02
.76E+03,1.89E+03,9.30E+02
.85E+03,1.35E+03,1.12E+03
.20E+02,5.17E+02,7.49E+02
.96E+03,1.82E+03,9.30E+01
7E+03,2.08E+03,1.02E+03
.32E+03,1.43E+03,3.08E+02
.48E+02,1.85E+02,7.10E+01
.21E+04,2.49E+04,2.18E+04
.60E+03,3.82E+03,2.60E+03
.95E+02,1.64E+03,1.66E+03
.75E+03,1.09E+04,7.49E+03
.24E+03,6.58E+01,9.04E+03
.78E+03,2.26E+02,9.37E+02
.56E+03,1.73E+03,8.50E+01
.82E+03,1.81E+03,1.19E+03
.80E+01,2.48E+03,1.48E+03
.50E+01,6.27E+02,5.45E+02
.58E+03,1.48E+03,2.53E+02
E+03,2.12E+03,7.27E+03
.26E+03,7.61E+02,1.02E+03
.84E+02,1.29E+03,2.05E+02
.96E+03,2.74E+03,3.79E+03

10E+03,1.26E+03,3.02E+03
03E+02,1.46E+03,1.47E+03
69E+03,1.77E+03,3.05E+02
83E+02,7.06E+02,3.05E+02
58E+03,1.37E+03,8.50E+02
47E+02,1.29E+03,7.52E+02
81E+02,1.84E+03,1.06E+03
80E+03,1.66E+03,2.98E+03
1E+03,1.82E+03,6.67E+03
45E+03,1.43E+03,1.78E+01
05E+03,1.80E+02,3.35E+02
87E+03,1.64E+03,8.83E+03
27E+03,1.66E+02,3.35E+02
63E+03,1.94E+03,1.08E+03
00E+03,5.35E+02,2.09E+02
06E+03,1.40E+03,1.25E+03
05E+01,2.05E+03,9.52E+02
6E+03,1.89E+03,1.58E+03
70E+03,1.46E+03,8.84E+02
51E+03,4.22E+03,4.81E+03
48E+02,2.70E+02,1.74E+03
72E+03,1.12E+03,1.86E+03
80E+03,1.08E+03,1.26E+03
14E+03,2.53E+02,2.11E+03
44E+03,1.41E+03,1.63E+03
66E+02,6.30E+02,1.65E+03
28E+02,1.63E+03,2.57E+01
91E+03,1.44E+03,1.03E+03
76E+02,2.46E+02,5.31E+02
64E+02,5.72E+02,1.08E+03
40E+02,1.84E+03,6.03E+02
14E+03,8.51E+02,1.09E+03
76E+04,3.67E+04,2.01E+04
33E+04,2.19E+04,1.83E+04
84E+03,2.54E+03,1.18E+03
53E+03,1.83E+03,1.24E+03
60E+03,5.47E+03,2.69E+03
67E+01,3.09E+03,2.85E+03
85E+03,3.09E+02,2.92E+03
23E+03,9.60E+02,6.50E+01
48E+02,1.51E+03,6.50E+01
00E+03,1.91E+03,6.50E+01
04E+02,1.69E+03,3.60E+01
39E+02,1.28E+02,4.70E+01

.71E+03,2.67E+03,1.39E+03
.36E+03,1.18E+03,8.52E+02
.11E+03,2.64E+03,1.35E+03
.65E+03,2.10E+03,7.53E+02
1E+03,1.49E+03,4.74E+02
.56E+03,1.41E+03,3.23E+02
.26E+03,1.38E+03,3.96E+02
.45E+02,5.73E+02,1.29E+02
.27E+02,1.77E+03,1.16E+03
0E+03,2.28E+03,3.50E+01
.35E+03,9.08E+01,1.53E+03
.26E+03,2.13E+03,4.17E+02
.41E+02,6.44E+02,3.50E+01
.41E+02,6.44E+02,3.50E+01
.39E+03,1.99E+03,7.27E+02
.71E+02,3.12E+03,4.76E+02
.43E+03,1.52E+03,4.38E+02
.22E+02,1.48E+02,5.82E+02
.70E+02,2.50E+01,6.75E+01
.33E+03,1.90E+03,2.66E+02
.30E+02,1.11E+03,4.26E+02
.60E+01,1.12E+02,8.63E+02
.79E+02,7.09E+02,1.69E+02
3E+02,9.68E+02,3.10E+02
.33E+03,3.07E+03,7.44E+02
.65E+03,2.31E+03,2.03E+02
.85E+03,1.83E+02,1.85E+03
.42E+03,2.29E+03,1.07E+03
.03E+03,1.76E+03,1.45E+03
.75E+03,2.30E+03,1.85E+03
.55E+03,1.49E+02,8.15E+02
0E+03,1.43E+03,2.11E+03
.14E+03,1.49E+03,2.33E+03
.35E+03,1.89E+03,9.23E+02
.42E+02,2.03E+03,1.03E+03
.36E+03,1.71E+03,1.72E+03
.71E+03,1.38E+03,1.10E+03
.45E+03,4.79E+03,3.98E+03
.24E+03,2.97E+03,1.20E+04
.51E+03,7.54E+02,1.14E+03
.81E+03,1.44E+03,1.17E+03
.12E+03,1.11E+02,1.34E+03
.85E+03,1.58E+03,8.62E+02
.15E+03,4.26E+03,5.87E+03

.40E+03,1.13E+03,9.11E+02
.84E+03,1.98E+03,5.08E+02
.35E+03,6.51E+02,3.14E+02
E+03,2.25E+03,1.76E+03
.56E+03,7.77E+02,2.25E+02
.76E+02,2.08E+02,8.18E+02
.89E+02,1.43E+03,1.61E+02
.75E+03,1.56E+03,1.21E+03
.80E+03,2.66E+03,2.56E+03
.30E+03,1.04E+03,7.97E+02
.29E+03,1.17E+03,1.34E+03
.06E+03,1.81E+03,6.27E+02
.84E+03,2.83E+03,2.49E+03
.08E+03,2.65E+03,1.31E+03
.45E+03,8.66E+02,4.50E+02
0E+03,2.20E+03,1.28E+03
.69E+02,2.19E+03,8.45E+02
0E+03,1.69E+03,6.74E+02
.06E+03,8.13E+02,9.67E+02
1E+03,2.58E+03,8.93E+02
.84E+03,1.46E+03,1.84E+03
.94E+03,2.03E+03,1.06E+03
1E+02,1.53E+03,6.70E+01
.05E+03,1.43E+03,1.04E+03
.53E+03,9.85E+02,1.57E+03
.02E+03,5.94E+03,2.29E+03
.53E+02,1.91E+03,6.45E+02
.73E+02,1.18E+02,5.20E+01
9E+02,1.59E+03,1.18E+03
.92E+03,3.31E+03,1.14E+04
.42E+03,8.64E+02,2.62E+03
.14E+03,8.64E+02,2.61E+03
.76E+03,1.85E+03,8.95E+02
8E+03,2.07E+03,6.10E+01
2E+03,2.82E+03,8.00E+01
.85E+03,2.99E+03,1.54E+03
.79E+03,1.52E+03,1.27E+03
.78E+03,2.25E+03,1.97E+03
.55E+02,1.78E+03,1.52E+03
.23E+02,9.65E+02,1.09E+03
.61E+03,1.71E+03,5.69E+02
.66E+03,2.08E+03,1.91E+03
4E+03,1.82E+03,2.55E+02
.16E+03,1.50E+03,7.24E+02

```
.42E+03,3.37E+03,5.10E+03
.31E+03,6.49E+02,1.22E+03
.10E+02,1.19E+03,4.30E+01
.97E+02,9.40E+01,8.74E+02
.71E+03,2.58E+03,8.61E+02
.38E+03,1.22E+03,1.49E+03
.34E+03,1.95E+03,1.49E+03
.27E+01,2.54E+03,2.13E+03
.61E+03,2.32E+03,1.16E+03
2E+03,9.48E+01,1.15E+03
.06E+03,1.74E+03,1.80E+03
.26E+02,2.21E+03,6.14E+02
.19E+03,6.87E+02,1.25E+03
E+02,1.78E+03,3.94E+03
8E+03,8.00E+02,3.74E+02
.45E+02,9.26E+02,2.30E+03
.79E+02,4.43E+02,1.96E+03
.33E+03,6.89E+03,6.24E+03
.01E+03,3.87E+03,4.00E+03
.33E+03,3.27E+03,4.37E+02
.10E+03,3.09E+03,1.10E+03
.22E+03,1.33E+03,1.14E+03
.15E+03,1.29E+04,1.43E+04
.02E+03,8.27E+03,8.20E+03
.33E+03,2.99E+03,2.42E+03
.01E+03,8.44E+02,9.34E+02
.23E+03,1.65E+03,1.39E+03
.27E+02,2.25E+03,1.30E+03
.27E+02,1.42E+03,1.96E+03
.81E+03,2.03E+03,7.93E+02
.36E+02,1.92E+03,5.57E+02
.24E+02,8.77E+02,1.86E+03
.00E+02,2.28E+03,1.52E+02
.87E+03,1.12E+04,1.17E+04
.00E+03,3.15E+03,4.01E+03
6E+03,1.99E+03,2.18E+03
.32E+03,4.70E+03,8.65E+03
.53E+03,3.20E+03,2.38E+03
.39E+03,2.44E+03,1.86E+03
.65E+03,3.14E+03,1.51E+03
.49E+03,1.59E+04,1.32E+04
.68E+03,4.39E+03,4.80E+03
.42E+03,8.54E+01,1.04E+03
.00E+03,2.30E+01,1.84E+03
```

93E+03,2.28E+03,2.67E+03
06E+03,3.36E+03,1.18E+04
74E+03,2.54E+03,7.50E+03
41E+03,3.19E+03,1.10E+03
14E+03,2.16E+03,7.30E+03
10E+03,2.99E+03,1.24E+03
66E+03,2.79E+03,5.65E+02
32E+02,1.60E+02,5.41E+02
